# A Likelihood Approach for Real-Time Calibration of Stochastic Compartmental Epidemic Models

**DOI:** 10.1371/journal.pcbi.1005257

**Published:** 2017-01-17

**Authors:** Christoph Zimmer, Reza Yaesoubi, Ted Cohen

**Affiliations:** 1 Epidemiology of Microbial Diseases, Yale School of Public Health, New Haven, Connecticut, United States of America; 2 Health Policy and Management, Yale School of Public Health, New Haven, Connecticut, United States of America; Johns Hopkins Bloomberg School of Public Health, UNITED STATES

## Abstract

Stochastic transmission dynamic models are especially useful for studying the early emergence of novel pathogens given the importance of chance events when the number of infectious individuals is small. However, methods for parameter estimation and prediction for these types of stochastic models remain limited. In this manuscript, we describe a calibration and prediction framework for stochastic compartmental transmission models of epidemics. The proposed method, Multiple Shooting for Stochastic systems (MSS), applies a linear noise approximation to describe the size of the fluctuations, and uses each new surveillance observation to update the belief about the true epidemic state. Using simulated outbreaks of a novel viral pathogen, we evaluate the accuracy of MSS for real-time parameter estimation and prediction during epidemics. We assume that weekly counts for the number of new diagnosed cases are available and serve as an imperfect proxy of incidence. We show that MSS produces accurate estimates of key epidemic parameters (i.e. mean duration of infectiousness, *R*_0_, and *R*_eff_) and can provide an accurate estimate of the unobserved number of infectious individuals during the course of an epidemic. MSS also allows for accurate prediction of the number and timing of future hospitalizations and the overall attack rate. We compare the performance of MSS to three state-of-the-art benchmark methods: 1) a likelihood approximation with an assumption of independent Poisson observations; 2) a particle filtering method; and 3) an ensemble Kalman filter method. We find that MSS significantly outperforms each of these three benchmark methods in the majority of epidemic scenarios tested. In summary, MSS is a promising method that may improve on current approaches for calibration and prediction using stochastic models of epidemics.

## Author Summary

The sporadic emergence and spread of novel human pathogens poses a continuing threat to global public health. Early and accurate prediction of epidemic behavior is needed to inform effective public health policy decisions which much balance the risk of major outbreaks with the substantial costs of interventions. Key parameters governing the behavior of epidemics, however, cannot be directly observed and hence computational techniques are required for parameter estimation and prediction on the basis of imperfect surveillance data. In this paper, we develop a method (Multiple Shooting for Stochastic Systems, MSS) that utilizes accumulating epidemic data to estimate in real-time (1) key epidemic parameters including the average number of secondary cases and the mean duration of infectiousness, (2) the future number of cases, and (3) the unobserved number of infected individuals in the population. Employing comprehensive simulation experiments, we demonstrate that MSS outperforms the existing state-of-the-art calibration and prediction techniques in the majority of simulated scenarios. MSS may thus allow policy makers to respond more effectively and use resources more efficiently in the face of emerging epidemic threats.

## Introduction

The sporadic emergence of novel human pathogens (e.g. SARS, MERS, new strains of influenza) serves as a reminder of the importance of monitoring the spillover into and spread of pathogens in human populations. Accurate estimates of the fundamental epidemic parameters during the earliest phase of disease emergence can facilitate rational public health policy decisions [1, 2] and are thus of high priority [3, 4].

Estimating epidemic parameters is challenging during this initial period of disease emergence because the dynamics are stochastic and the processes governing disease spread are complex and usually only partially observable. Public health decision makers, therefore, require tools to make inference about the true state of the epidemic and the values of fundamental epidemic parameters using observations that can only imperfectly represent the true epidemic state (e.g. reported disease-related illnesses serve as an imperfect measure of incident cases).

There has been substantial recent progress in the development of methods to infer epidemic parameters from partial epidemic observations [5–11]. For example, infection network models are commonly used when symptom onset date for each reported case is available [12–17]. In these models, disease spread is described as a directed network in which the nodes represent cases and the directed edges between nodes represent transmission links. In the absence of the type of detailed individual-level data required by infection network methods, compartmental transmission dynamic models have been used for parameter estimation and for projecting epidemic trajectory. These models divide the population into disjoint subgroups (e.g. susceptible, infectious, and recovered) and transitions between the epidemic states are described using ordinary differential equations (for deterministic models) [18] or Markov chains (for stochastic models) [19].

A wide variety of methods have been described to calibrate these compartmental models which differ based how they estimate the likelihood of observations. These methods make use of deterministic [20–22] or stochastic [23] models to describe the underlying epidemic process, and may involve Markov Chain Monte Carlo [24] or filtering techniques [25–32] to sample parameter space and the unobserved states.

In this paper, we describe an alternative method for calibrating a general class of stochastic compartmental models using the types of data that would be available during the early period of epidemic spread. Unlike deterministic models, stochastic models capture chance events, a feature that proves essential for the accuracy of model-based parameter estimation and prediction during pathogen emergence.

Our calibration method, Multiple Shooting for Stochastic systems (MSS), utilizes a linear noise approximation (LNA) approach and a state-updating procedure to approximate the likelihood of observations while explicitly accounting for the interdependency between subsequent epidemic observations. Using simulation experiments, we compare the performance of MSS with several competing approaches in terms of accuracy in estimating parameters, predicting future behaviors, and inferring the current epidemic state. We test the sensitivity of the performance of these approaches to observational noise and model misspecification.

## Methods

### Problem formulation

**Fig 1:**
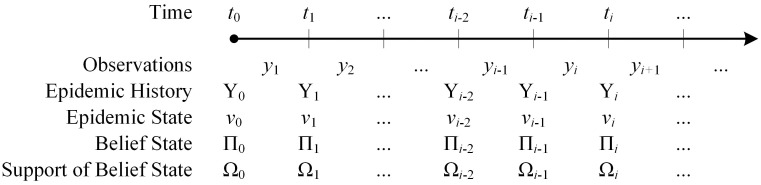
Sequence of observations during an epidemic.

During an epidemic, surveillance systems may be able to capture a variety of measures, such as the number of disease-related diagnoses, hospitalizations or deaths. We use *y*_*i*_ to denote the vector of epidemic measures that can be observed during the period [*t*_*i*−1_, *t*_*i*_] (see Fig 1). Let *Y*_*i*_ = (*y*_1_, *y*_2_, …, *y*_*i*_) be the epidemic history up to time *t*_*i*_. To measure the fit of an epidemic model to the observations accumulated up to time *t*_*i*_, we use the following likelihood function:

$$\begin{array}{c} L\left(Y_{i};\theta \right)\;=\text{Pr}\left(y_{1},y_{2},\ldots ,y_{i};\theta \right)=\;\prod_{i=1}^{n}\;P\left(y_{i}|y_{1},y_{2},\ldots ,y_{i-1};\theta \right), \end{array}$$ (1)

where $P(y_{i}|y_{1},y_{2},\ldots ,y_{i-1};\theta )$ is the probability of observing *y*_*i*_ given the previous observations (*y*_1_, *y*_2_, …, *y*_*i*−1_) and the model parameters *θ*.

For many epidemics, the probability function $P(\cdot |y_{1},y_{2},\ldots ,y_{i-1};\theta )$ may not be readily available and can be computationally expensive to evaluate. Therefore, the likelihood function (1) is often approximated by assuming that the epidemic observations in each period follow a discrete probability distribution (e.g. Poisson distribution) and are independently distributed across observation periods [23, 33]. For example, to approximate the likelihood function (1), Riley and colleagues assume that epidemic observations follow independent Poisson distributions where means are chosen to match the means generated from 250 model replications [23]. The method we develop here to approximate the likelihood function (1) does not retain the unrealistic assumption that these observations are independent.

Below, we first describe an algorithm to efficiently approximate the probability function $P(\cdot |y_{1},y_{2},\ldots ,y_{i-1};\theta )$ which can then be used to evaluate the likelihood function (1) for observations accumulated up to any point in the epidemic.

### Approximating the likelihood function

In a compartmental model, the state of the epidemic at a given time *t*_*i*_ is defined as the number of individuals in each compartment at time *t*_*i*_ [19, 34], which we will refer to as *ν*_*i*_. In most settings, particularly if the disease has a complicated natural history, the true distribution of individuals in each state over the course of epidemic progression, *ν*_*i*_, *i* ∈ {0, 1, 2, …}, is not fully observable. Hence, to form statistical inference about the true epidemic state, we utilize belief states which define a probability distribution over all possible epidemic states given past observations.

Let Π(⋅|*Y*_*i*_) denote the belief state at time *t*_*i*_ given the accumulated observations *Y*_*i*_. Now by conditioning on the epidemic state at time *t*_*i*−1_, i.e. *ν*_*i*−1_, the probability function $P(\cdot |y_{1},y_{2},\ldots ,y_{i-1};\theta )$ in Eq (1) can be calculated as:

$$P\left(y_{i}|y_{1},y_{2},\ldots ,y_{i-1};\theta \right)=\sum_{\nu_{i-1}\in \Omega_{i-1}}P\left(y_{i}|\nu_{i-1};\theta \right)\;\Pi \left(\nu_{i-1}|y_{1},y_{2},\ldots ,y_{i-1};\theta \right),$$ (2)

where *Ω*_*i*−1_ is the set of feasible epidemic states (i.e. the support of the belief state Π) at time *t*_*i*−1_. By conditioning on the state of the epidemic at time *t*_*i*_, the probability function $P(\cdot |y_{1},y_{2},\ldots ,y_{i-1};\theta )$ in Eq (2) can be calculated as:

$$\begin{array}{cc} & P(y_{i}|y_{1},y_{2},\ldots ,y_{i-1};\theta )\\ & \;=\sum_{\nu_{i}\in \Omega_{i}}\;\sum_{\nu_{i-1}\in \Omega_{i-1}}P\left(y_{i}|\nu_{i},\nu_{i-1};\theta \right)p\left(\nu_{i}|\nu_{i-1};\theta \right)\;\Pi \left(\nu_{i-1}|y_{1},y_{2},\ldots ,y_{i-1};\theta \right). \end{array}$$ (3)

Calculating the probability function (3) can be computationally difficult. First, it requires calculating or approximating the transition probability *p*(*ν*_*i*_|*ν*_*i*−1_; *θ*) for each pair (*ν*_*i*_, *ν*_*i*−1_) ∈ *Ω*_*i*_ × *Ω*_*i*−1_, and second, it involves enumeration over the set *Ω*_*i*_ × *Ω*_*i*−1_, which can be prohibitively large even for simple epidemic models. One way to simplify the computational complexity of Eq (3) is to represent the belief state Π(⋅|*Y*_*i*_) as a step function that takes 1 for the most probable state (denoted by $\hat{\nu }$) and 0 elsewhere. This allows us to approximate the function P(·) in Eq (3) with:

$$\begin{array}{c} \tilde{P}\left(y_{i}|y_{1},y_{2},\ldots ,y_{i-1};\theta \right)=\sum_{\nu_{i}\in \Omega_{i}}\;P\left(y_{i}|\nu_{i},{\hat{\nu }}_{i-1};\theta \right)p\left(\nu_{i}|{\hat{\nu }}_{i-1};\theta \right), \end{array}$$ (4)

where ${\hat{\nu }}_{i-1}$ represents the most likely epidemic state given observations *Y*_*i*−1_ = (*y*_1_, *y*_2_, …, *y*_*i*−1_). By the definition of epidemic states, the transition from state *ν*_*i*−1_ to *ν*_*i*_ generates a unique set of observations, and it is trivial to find whether the state transition ${\hat{\nu }}_{i-1}$ to *ν*_*i*_ can generate the observation *y*_*i*_ (see design of performance analysis for an illustrative example). Therefore, for a given observation *y*_*i*_, the probability $P(y_{i}|\nu_{i},{\hat{\nu }}_{i-1};\theta )$ in Eq (4) is equal to 1 if the transition from state ${\hat{\nu }}_{i-1}$ to *ν*_*i*_ results in observing *y*_*i*_, and is zero otherwise. To calculate $\tilde{P}\left(y_{i}|{\hat{\nu }}_{i-1};\theta \right)$ in Eq (4), it only remains to identify the state transition probability function $p(\nu_{i}|{\hat{\nu }}_{i-1};\theta )$ and a state estimation scheme. In the next subsections, we propose an algorithm to achieve this.

#### Approximating state transition probabilities

Finding the exact state transition probability function *p*(⋅) can be difficult, and in many cases impossible, as state spaces in epidemic models can be quite large or unbounded. To overcome this problem, we employ a linear noise approximation (LNA) method to approximate the probability distribution of the new epidemic state *ν*_*i*_ given the previous state *ν*_*i*−1_, i.e. *p*(*ν*_*i*_|*ν*_*i*−1_; *θ*). The LNA has been previously used to estimate parameters of stochastic biochemical reaction models [35, 36]. Here we extend Zimmer and Sahle’s method [36] to calibrate stochastic epidemic models where the true epidemic state is only partially observable.

To approximate the probability distribution of *ν*_*i*_ given the state *ν*_*i*−1_, the LNA method uses an ordinary differential equations (ODE) model to approximate the *expected* behavior of the epidemic over the period [*t*_*i*−1_, *t*_*i*_] and to identify a co-variance matrix to characterize the uncertainty around the epidemic behavior over this interval. We use the following notation to denote the ODE epidemic model used by the LNA method:

$$\begin{array}{cc} \frac{d}{dt}x\left(t,x_{0};\theta \right)= & \Gamma \Lambda (x\left(t,x_{0};\theta \right),\theta ),\\ x(0,x_{0};\theta )= & x_{0}. \end{array}$$ (5)

In the ODE system (5), the vector *x*(*t*, *x*_0_; *θ*) is the epidemic state of the ODE model at time *t* given the initial state *x*_0_, the vector Λ(*x*(*t*, *x*_0_; *θ*), *θ*) denotes the instantaneous changes in the epidemic when at state *x*(*t*, *x*_0_; *θ*), and the matrix Γ describes how the instantaneous changes at time *t* impact the epidemic state at time *t* + Δ*t* (see subsequent sections for an example).

The LNA assumes that the probability distribution of *ν*_*i*_|*ν*_*i*−1_ can be properly approximated by a normal distribution $N(\mu_{i},{\mathrm{cov}}_{i})$. The mean vector *μ*_*i*_ is the solution of ODE system (5) with *ν*_*i*−1_ as the initial condition (i.e. *μ*_*i*_ = *x*(*t*_*i*_ − *t*_*i*−1_, *ν*_*i*−1_; *θ*)) and the variance matrix cov_*i*_ = Σ(*t*_*i*_ − *t*_*i*−1_, *ν*_*i*−1_; *θ*) is the solution of the following ODE systems [37, 38]:

$$\begin{array}{cc} \frac{d}{dt}\Sigma \left(t,\nu_{i-1};\theta \right) & =J\left(x,\theta \right)\Sigma \left(t,\nu_{i-1};\theta \right)+\Sigma \left(t,\nu_{i-1};\theta \right)J{\left(x,\theta \right)}^{T}+D\left(x;\theta \right)\\ \Sigma (0,\nu_{i-1};\theta ) & =0_{K\times K}. \end{array}$$ (6)

In the ODE system (6), $J\left(x,\theta \right)=\Gamma \frac{d}{dx}\Lambda \left(x,\theta \right)$ and *D* is a *K* × *K* matrix with the (*i*, *j*) entity equal to $\sum_{k=1}^{K}\Gamma_{jk}\Gamma_{jk}\Lambda \left(x,\theta \right)$, where *K* is the number of compartments in the epidemic model.

An important question remains about how well this proposed LNA method approximates the probability distribution of epidemic states. Relying on an extensive numerical analysis, we will demonstrate in the Results section that for the epidemic scenarios considered, our method yields accurate parameter estimations and reliable predictions. We also note that our method does not rely on a single LNA model to approximate the entire epidemic trajectory. For each observation period [*t*_*i*−1_, *t*_*i*_], it generates a new LNA model to approximate the epidemic behavior only over this particular period.

#### Updating belief states

We now describe how to update our belief about the true state of the epidemic, denoted by Π(⋅), once new observations *y*_*i*_ are obtained. We first note that Π(⋅) is defined to return 1 for the most likely state $\hat{\nu }$, and zero elsewhere. Therefore, given the state ${\hat{\nu }}_{i-1}$ at time *t*_*i*−1_, the probability of observing *y*_*i*_ during the interval [*t*_*i*−1_, *t*_*i*_] is equal to $P\left(y_{i}|\nu_{i},{\hat{\nu }}_{i-1};\theta \right)p\left(\nu_{i}|{\hat{\nu }}_{i-1};\theta \right)$ (see the discussion prior to Eq (3)).

Now, the most probable state at time *t*_*i*_, ${\hat{\nu }}_{i}$, is the one that leads to the highest probability of observing *y*_*i*_:

$$\begin{array}{c} {\hat{\nu }}_{i}=\underset{\nu_{i}\in \Omega_{i}}{\arg\max}\;P\left(y_{i}|\nu_{i},{\hat{\nu }}_{i-1};\theta \right)p\left(\nu_{i}|{\hat{\nu }}_{i-1};\theta \right). \end{array}$$ (7)

As discussed above, $P(y_{i}|\nu_{i},{\hat{\nu }}_{i-1};\theta )$ is either 0 or 1, and the probability function $p(\cdot |{\hat{\nu }}_{i-1};\theta )$ is approximated with a Normal distribution. Therefore, it is straightforward to solve the optimization problem (7). Note that in compartmental models, the support *Ω*_*i*_ can be recursively updated as new observations become available (see [39] for details and the S1 Text subsection 8 “Detailed pseudo code for our MSS” for an illustration).

### Estimating model parameters

One of the main goals of model calibration is to leverage information from accumulating observations to characterize the uncertainty around model parameters. To this end, we employ a Bayesian approach that updates the probabilistic information on model parameters as new observations become available. This updating is performed according to the following Bayes rule:

$$\pi_{i}\left(\theta |Y_{i}\right)\propto L^{\left(\text{MSS}\right)}\left(y_{i}|\theta \right)\pi_{i-1}\left(\theta |Y_{i-1}\right)$$ (8)

where *π*_*i*−1_(⋅, *Y*_*i*−1_) is the prior distribution before obtaining the *i*^*th*^ observation and *π*_*i*_(⋅|*Y*_*i*_) is the posterior distribution after obtaining the *i*^*th*^ observation. In Eq (8), the likelihood function *L*^(MSS)^(⋅|*θ*) is estimated using the approach described in the preceding sections. Note that *π* is used for both prior and posterior as the posterior is recursively calculated from the prior upon obtaining subsequent observations.

**Fig 2:**
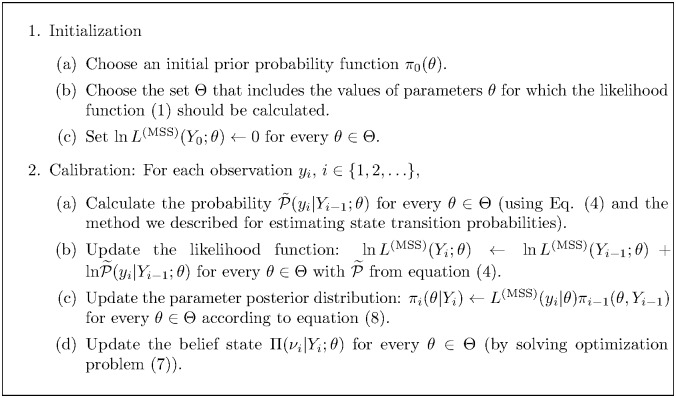
An algorithm for real-time calibration of stochastic compartmental epidemic models.

Fig 2 summarizes the steps of our proposed method for calibrating stochastic epidemic models. The set Θ in Step 1.b includes parameter values for which the likelihood function (1) should be calculated. This set can be built by sampling from the prior distribution *π*_0_(*θ*) or via other sampling methods including random, Latin hypercube, or orthogonal sampling. Obviously, the sample set Θ can be modified as new observations become available and the prior distribution *π*_0_(*θ*) is being updated. However, the main advantage of fixing the sample set Θ at the beginning of the algorithm is to allow the algorithm to update the likelihood function (1) in a recursive manner as new observations are accumulating over time. That is, to find the likelihood value *L*^(MSS)^(*Y*_*i*_; *θ*) at time *t*_*i*_, we only need to know the value of the likelihood function from the previous time step (i.e. *L*^(MSS)^(*Y*_*i*−1_; *θ*)) and the probability P(*y*_*i*_|*Y*_*i*−1_; *θ*) (see Step 2.c). The parameter posterior distribution obtained by using observations *Y*_*i*−1_ can be used as a prior distribution once a new observation *y*_*i*_ becomes available.

### Predicting epidemic behavior

A central motivation for developing better methods for calibration of stochastic epidemic models is to improve predictions about the future course of epidemics.

To demonstrate how our method can be used for prediction, let *Z* denote the quantity that we wish to predict (e.g. the number of diagnoses during the subsequent week) and $P(Z|\nu_{i};\theta )$ denote the probability density function of the random variable *Z* if the epidemic is at state *ν*_*i*_ ∈ *Ω*_*i*_ at time *t*_*i*_ and the epidemic parameters have value *θ* ∈ Θ. Now, the probability distribution of the random variable *Z* given the accumulated observations at time *t*_*i*_, *Y*_*i*_ = (*y*_1_, *y*_2_, …, *y*_*i*_), can be calculated as:

$$P\left(Z|Y_{i}\right)=\int_{\theta \in \Theta }\int_{\nu_{i}\in \Omega_{i}}P\left(Z|\nu_{i};\theta \right)\;\Pi \left(\nu_{i}|Y_{i};\theta \right)\;\pi_{i}\left(\theta |Y_{i}\right)\;d\nu_{i}\;d\theta .$$ (9)

In Eq (9), the belief state Π(⋅|*Y*_*i*_; *θ*) and the parameter posterior distribution *π*_*i*_(⋅|*Y*_*i*_) are both obtained from the algorithm described in Fig 2. To calculate the distribution of the random variable *Z*|*Y*_*i*_, we assume that we have access to a stochastic simulation model to sample from the random variable *Z*|*ν*_*i*_ for a given set of parameter values *θ*. For the numerical results presented here, we use the Gillespie algorithm [40] (see SI document) to generate realizations for *Z*|*Y*_*i*_ that can be used to estimate the probability function *P*(*Z*|*Y*_*i*_) in Eq (9), or to calculate mean or other moments of the random variable *Z*|*Y*_*i*_.

### Benchmark methods

We compare the performance of our method with three competing state-of-the-art approaches. The first benchmark method, which is based on an assumption of independent Poisson observations, is straightforward to understand and to implement and has been used to infer basic fundamental parameters during the early appearance of novel pathogens [23]. For the second and third approaches, we consider Particle Filter and Ensemble Kalman Filter [27] as these methods won the 2014 “Predict the Influenza Season Challenge” sponsored by the U.S. Center for Disease Control and Prevention [41] and also demonstrate competitive performance against four other methods in a recent comparison study [27].

#### Benchmark method A: Likelihood approximation with the assumption of independent Poisson observations (I.Poi)

We use the method from Riley and colleagues [23] as the first benchmark. This method assumes that the observations *y*_*i*_ are independent and factorizes the likelihood function *L*(*Y*_*i*_; *θ*) (Eq (1)) to

$$\begin{array}{c} L^{\left(\text{I}.\text{Poi}\right)}\left(Y_{i}|\theta \right)=\prod_{i=1}^{n}P^{\left(\text{I}.\text{Poi}\right)}\left(y_{i}|\theta \right), \end{array}$$ (10)

where $P^{\left(\text{I}.\text{Poi}\right)}\left(\cdot |\theta \right)∼Poisson\left(\mu_{i}\right)$. To calculate the mean *μ*_*i*_, we obtain 1000 stochastic trajectories using parameter values *θ* and shift them so that the first observation for each trajectory lies within the first observation period [*t*_0_, *t*_1_]. We then calculate the mean of observations over period [*t*_*i*−1_, *t*_*i*_] (i.e. *μ*_*i*_) using these simulated trajectories. This approach ignores the inter-dependencies between observations *y*_1_, *y*_2_, …, *y*_*i*_ and approximates $P(y_{i}|y_{1},\ldots ,y_{i-1};\theta )$ by $P^{\left(\text{I}.\text{Poi}\right)}\left(y_{i};\theta \right)$.

Without the knowledge of an updated belief state Π(⋅|*ν*_*i*_; *θ*), Eq (9) for prediction reduces to

$$P^{\left(\text{I}.\text{Poi}\right)}\left(Z|Y_{i}\right)=\sum_{\theta \in \Theta }P^{\left(\text{I}.\text{Poi}\right)}\left(Z|\theta \right)\pi_{i}\left(\theta |Y_{i}\right).$$ (11)

The mean and variance of random variable *Z* can be estimated using simulated trajectories with parameter values selected according to the probability function *π*_*i*_(*θ*|*Y*_*i*_). These simulated trajectories can also be used to make inference about the epidemic state at time *t*_*i*_. To make predictions at time *t*_*i*_, our implementation of the benchmark approach uses only simulated trajectories that have not been extinguished before *t*_*i*_.

#### Benchmark method B: Particle Filter

The Particle Filter (PF) approach described by [27] also seeks to approximate the likelihood function (1) to calculate an a-posteriori distribution *π*_*i*_(*θ*|*Y*_*i*_) using the epidemic history *Y*_*i*_. This method, however, uses a different approximation to calculate the probability $P(y_{i}|Y_{i-1};\theta )$:

$$\begin{array}{cc} & P^{\left(\text{PF}\right)}\left(y_{i}|Y_{i-1};\theta \right)=\\ & \sum_{\nu_{i}\in \Omega_{i}}\;\sum_{\nu_{i-1}\in \Omega_{i-1}}P^{\left(\text{PF}\right)}\left(y_{i}|\nu_{i},\nu_{i-1};\theta \right)p^{\left(\text{PF}\right)}\left(\nu_{i}|\nu_{i-1};\theta \right)\;\Pi^{\left(\text{PF}\right)}\left(\nu_{i-1}|y_{1},y_{2},\ldots ,y_{i-1};\theta \right). \end{array}$$ (12)

While the belief state is also a point distribution, the state update is performed based on the solution of ODE system (5):

$$\begin{array}{c} {\hat{\nu }}_{i}=x\left(t_{i}-t_{i-1},{\hat{\nu }}_{i-1};\theta \right). \end{array}$$

In Eq (12), the forward propagation *p*^(PF)^(*ν*_*i*_|*ν*_*i*−1_; *θ*) is a point distribution with mass 1 at ${\hat{\nu }}_{i}$, and *P*^(PF)^(*y*_*i*_|*ν*_*i*_, *ν*_*i*−1_; *θ*) is assumed to follow a normal distribution $N(h\left({\hat{\nu }}_{i}\right),\sigma_{obs,i}^{2})$ where the function *h* maps state to observations and the observational variance $\sigma_{obs,i}^{2}$ is assumed to be:

$$\begin{array}{c} \sigma_{obs,i}^{2}=10000+\frac{1}{50}{\left(\frac{1}{3}\sum_{j=i-3}^{i-1}y_{j}\right)}^{2}. \end{array}$$ (13)

This observational variance is identical to the one used in [27]. Our sensitivity analysis reveals that the choice of observational variance, Eq (13), does not have a significant effect on the comparative performance of PF compared to the methods studied here (see Discussion).

Since this approach calculates belief states and posterior distribution for *θ*, the prediction is performed in an identical fashion as for the MSS approach as described in Eq (9). The pseudo code and the full implementation of this approach for an epidemic model is described in the SI (S1 Text subsection 6 “Pseudo code for the PF” and S1 Text subsection 10 “Detailed pseudo code for PF”).

#### Benchmark method C: Ensemble Kalman Filter (EnKF)

We selected the ensemble Kalman Filter (EnKF) [27] for the third benchmark method. EnKF does not use a likelihood function *L*(*Y*_*i*_; *θ*) and instead it propagates an initial ensemble that contains both epidemic states and epidemic parameters. Each new observation is then used to update this ensemble. After such updating, the ensemble is considered as a sample from the posterior distribution for both the parameters and the states.

Similar to PF and MSS, this approach calculates belief states and posterior distribution for *θ*, and hence predictions can be performed using Eq (9). The pseudo code and the full implementation of this approach for an epidemic model is described in the SI (S1 Text subsection 7 “Pseudo code for the EnKF” and S1 Text subsection 11 “Detailed pseudo code for the EnKF”).

### Design of the performance analysis

**Fig 3:**
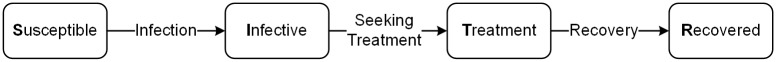
A model for the outbreak of a novel viral pathogen.

To compare the performance of our approach with that of the three competing benchmark methods (described above), we use several different simulated scenarios after the introduction of a novel viral pathogen epidemic. We develop a simple stochastic compartmental model where population members are grouped into four mutually exclusive compartments (see Fig 3). In this model, Infective individuals may infect Susceptibles with whom they come into contact. We assume that Infective individuals will eventually seek treatment due to worsening symptoms (and move to the Treatment compartment). Here, cases under treatment do not transmit infection and those who recover from the disease have full immunity against reinfection with the pathogen (and moved to the Recovered compartment).

Let *x*_*S*_(*t*), *x*_*I*_(*t*), *x*_*T*_(*t*) and *x*_*R*_(*t*), respectively, denote the number of individuals in compartments Susceptible, Infective, Treatment, and Recovered, and *N*(*t*) = *x*_*S*_(*t*) + *x*_*I*_(*t*) + *x*_*T*_(*t*) + *x*_*R*_(*t*) denote the population size at time *t*. Disease transmission can be modeled using the following ODE model:

$$\begin{array}{cc} \frac{d}{dt}x_{S}\left(t\right) & =-\theta_{1}x_{S}\left(t\right)\frac{x_{I}\left(t\right)}{N(t)},\\ \frac{d}{dt}x_{I}\left(t\right) & =\theta_{1}x_{S}\left(t\right)\frac{x_{I}\left(t\right)}{N(t)}-\theta_{2}x_{I}\left(t\right),\\ \frac{d}{dt}x_{T}\left(t\right) & =\theta_{2}x_{I}\left(t\right)-\theta_{3}x_{t}\left(t\right),\\ \frac{d}{dt}x_{R}\left(t\right) & =\theta_{3}x_{T}\left(t\right) \end{array}$$ (14)

where *θ*_1_ is the disease transmission rate, *θ*_2_ is the rate of seeking treatment while infectious, and *θ*_3_ is the rate of recovering. This model can be presented in the format of the ODE system (5) by choosing

$$\Lambda \left(x\left(t,x_{0};\theta \right),\theta \right)=\left[\begin{array}{c} \theta_{1}x_{S}\left(t\right)\frac{x_{I}\left(t\right)}{N(t)}\\ \theta_{2}x_{I}\left(t\right)\\ \theta_{3}x_{T}\left(t\right) \end{array}\right]\;\text{and}\;\Gamma =\left[\begin{array}{ccc} -1 & 0 & 0\\ 1 & -1 & 0\\ 0 & 1 & -1\\ 0 & 0 & 1 \end{array}\right].$$ (15)

In our analysis, we assume that at time *t* = 0 one population member becomes infected. The model initial condition can therefore be defined as *x*_0_ = (*x*_*S*_(0), *x*_*I*_(0), *x*_*T*_(0), *x*_*R*_(0)) = (*N* − 1, 1, 0, 0).

**Fig 4:**
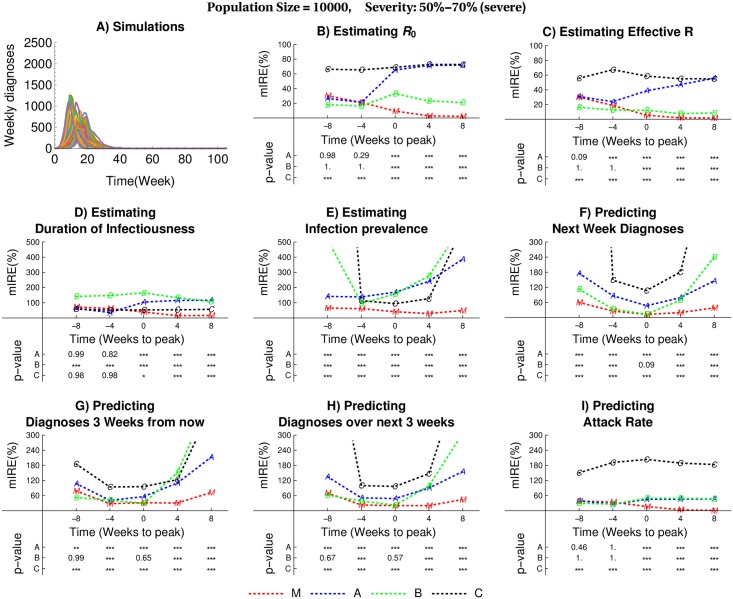
Median integrated relative errors (mIRE) in estimating model parameters and infection prevalence as well as prediction for simulated epidemics in a population of 10 000 with 50% − 70% attack rate. A) Epidemic scenarios used to evaluate the performance of calibration methods. Each scenario consists of 50 stochastic trajectories obtained by simulating the model in Fig 3 using the Gillespie algorithm. B) results for estimating *R*_0_, C) results for estimating *R*_eff_, D) results for estimating the mean duration of infectiousness, E) results for estimating the infection prevalence, F) results for predicting the next week diagnoses, G) results for predicting the diagnoses 3 weeks from now, H) results for predicting the diagnoses over the next 3 weeks, I) results for predicting the attack rate. In each panel: MSS: Multiple Shooting for Stochastic systems (the method proposed here); I.Poi: Independent Poisson (Benchmark Method A); PF: Particle Filter (Benchmark Method B); EnKF: Ensemble Kalman Filter (Benchmark Method C). P-values are from Wilcoxon Signed-Rank test evaluating the hypothesis that the median of relative errors for the MSS approach is smaller than that of I.Poi (first row), PF (second row), and EnKF (third row); p-values smaller than 0.001 are displayed as ***, p-values in between 0.001 and 0.01 as ** and between 0.01 and 0.05 as *. The values of mIRE for some scenarios fall above the vertical axis range and are not displayed.

To evaluate the performance of the calibration methods considered here, we used nine epidemic scenarios that differ by population size *N* ∈ {1000, 10,000, 10,0000} and the epidemic attack rate (moderate: [30% − 50%], severe: [50% − 70%] and extreme [70% − 100%]). To produce epidemic trajectories for each scenario, we first drew *R*_0_ from *U*([1, 3]) and mean duration of infectiousness from *U*([1, 20]), and then used the Gillespie algorithm [40] (see S1 Text subsection 1 “The Gillespie Algorithm” implemented in the software COPASI [42]) to simulate the SITR model of Fig 3 with $\theta_{1}=\frac{R_{0}}{\text{mean}\;\text{duration}\;\text{of}\;\text{infectiousness}}$ and $\theta_{2}=\frac{1}{\text{mean}\;\text{duration}\;\text{of}\;\text{infectiousness}}$ (Eq 14). We assumed that the delay until recovery after the onset of treatment is 4 days and that this is a known quantity (that is, *θ*_3_ = 0.25). To ensure that our selected simulated trajectories reflect expected shapes of outbreaks (rather than slow, simmering transmission), we only included trajectories with an epidemic peak that occurs between weeks 10 and 20 after take-off. Fig 4A and S1 to S8 Figs show the 50 simulated trajectories chosen for each scenario. In our evaluation, we assumed that the weekly number of disease-associated diagnoses was observed throughout the outbreak; this was calculated for each week as the number of people that transitioned from *I* to *T*. For our model, the number of diagnoses in week *i* is calculated as *x*_*T*_(*t*_*i*_)+*x*_*R*_(*t*_*i*_) − *x*_*T*_(*t*_*i*−1_) − *x*_*R*_(*t*_*i*−1_), where *t*_*i*_ − *t*_*i*−1_ = 7 days to obtain weekly data.

We implemented each of the calibration methods in Mathematica [43] and evaluated their performance based on their ability to accurately (1) estimate *R*_0_, effective *R* (*R*_eff_), the duration of infectiousness, and the current number of infectious individuals, and (2) predict the incident number of diagnoses (for the subsequent week and 3 weeks in the future) and the total cumulative cases (over the subsequent 3 weeks and over the whole epidemic). We note that in our analysis, we calibrate *R*_0_ and mean duration of infectiousness, and *θ*_1_ and *θ*_2_ are calculated based on the samples of *R*_0_ and mean duration of infectiousness for likelihood evaluations.

For each simulated trajectory shown in Fig 4A and S1A to S8A Figs, we evaluated the performance of our method at several different times during the epidemic: 8 weeks to peak incidence, 4 weeks to peak, at peak, 4 weeks after the peak, and 8 weeks after the peak. To quantify the performance of these methods based on each target metric *M*_*i*_ (e.g. estimating *R*_0_ or prediction of the number of incident cases in the subsequent period after observing *Y*_*i*_), we used the integrated relative error (IRE):

$$IRE\left(M_{i}\right)\;=\;100\int_{\bar{\Theta }}\int_{\Psi_{i}}\frac{|{\tilde{M}}_{i}-m|}{{\tilde{M}}_{i}}\;f_{M_{i}|\theta }\left(m|\theta \right)\pi_{i}\left(\theta |Y_{i}\right)\;dm\;d\theta$$ (16)

where *f*_*M*_*i*_|*θ*_(*m*|*θ*) is the probability density function of target *M*_*i*_ given the estimated parameter values *θ*, $\tilde{M_{i}}$ is the true value of target *M*_*i*_. Ψ_*i*_ is the set of possible values that the target *M*_*i*_ can take (i.e. the support of the probability density function *f*), and $\bar{\Theta }$ represents the set of all parameter values (see SI for further details). We chose IRE as the performance metric since it provides a precise measure for how well the mass of the posterior distribution covers the true value of the targets to estimate. Alternative performance metrics such as relative error or mean square error do not account for the variance of predictions and hence are not suitable for this purpose.

When fitting the model in Fig 3 to the simulated trajectories, we did not assume that the length of the period between the onset of the outbreak and the first observation is known. We therefore considered this delay as an additional model parameter that must be estimated through the calibration procedures in MSS.

## Results

### Performance evaluation: Parameter estimation

Fig 4B–4E displays the median of IRE (defined in Eq (16)) in estimating *R*_0_, effective *R*, duration of infectiousness, and infection prevalence from applying calibration methods described above on 50 simulated trajectories shown in Fig 4A for a severe scenario with attack rate between 50% and 70% for a population of 10000. See S1 to S8 Figs for the performance of these methods under other epidemic and population size scenarios.

In all 9 scenarios (defined by epidemic severity and population size) and 5 estimation times (8 and 4 weeks to peak, at the peak, and 4 and 8 weeks after the peak), the MSS method either outperforms the competing benchmark methods or demonstrates similar performance. In particular, we note that in contrast to other approaches, the MSS method offers continuous improvement in the accuracy of parameter estimation as epidemics progress and more observations become available. At the peak and thereafter, MSS displays dominating performance, which is attributable to the more accurate likelihood approximation offered by this approach (see Fig 4B–4E and S1B–S1E to S8B–S8E Figs.

Furthermore, the MSS method performs significantly better than the other competing methods in estimating the true (and unobserved) prevalence of infection. This suggests that the state updating procedure utilized by MSS method is more effective than the state updating methods employed by PF and EnKF. It is worth noting that the high IRE for infection prevalence at the beginning and the end of epidemics is the result of the small number of infectious individuals during these phases. While this impacts the performance of all methods (including MSS), the effect is small for MSS as the state updating procedure leads to more reliable state estimates. Also, since the estimation of the effective *R* requires an estimate of the current number of susceptibles, the MSS method again shows a superior behavior.

**Fig 5:**
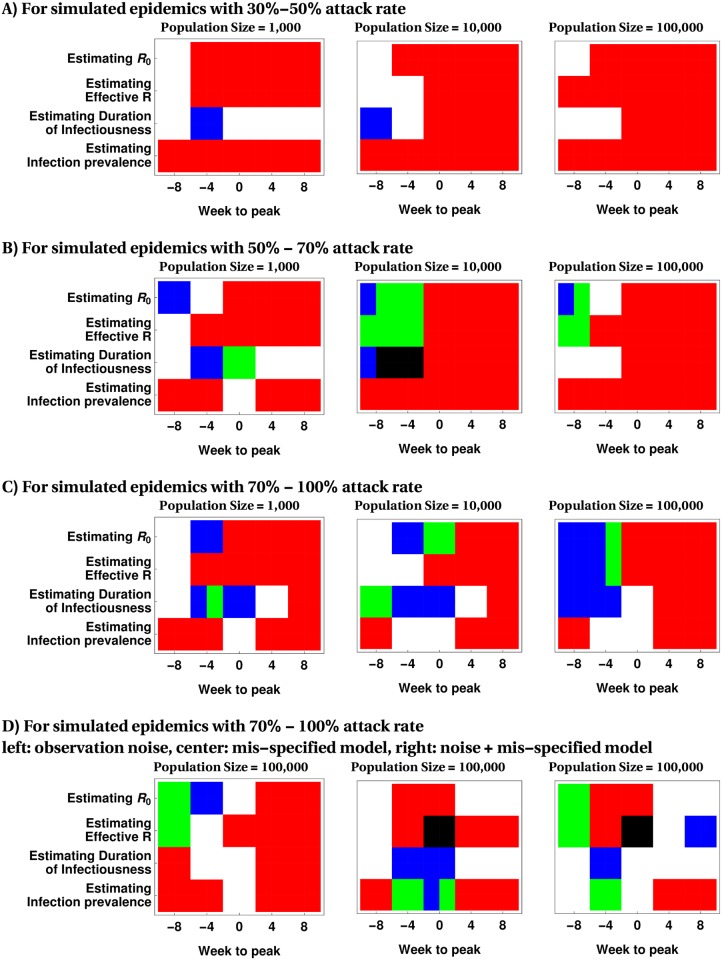
Identifying the calibration method with statistically dominant performance at 0.05 significance level for estimating model parameters and infection prevalence. Red, if MSS is statistically better than all benchmark methods. White, if all methods fail to demonstrate statistically dominant performance. Blue if I.Poi (Benchmark A), Green if Particle Filter (Benchmark B) and Black if the Ensemble Kalman filter (Benchmark C) statistically outperform the MSS method. Multiple colors are displayed if more than one method is significantly better than MSS.

Fig 5A–5C shows the results of testing the hypothesis that the MSS method outperforms all benchmark methods at 0.05 significance level at different epidemic times. This figure suggests that, in the majority of cases, the MSS method demonstrates either statistically superior or similar performance to the benchmark methods (represented by Green, Blue and Black colors). We note that even for the small number of situations in which MSS is statistically dominated by another benchmark method, the absolute difference in mIRE (as shown in Fig 4B–4E and S1B–S1E to S8B–S8E Figs is small.

The performance of each method tested does not change markedly between scenarios which differ by host population size (Fig 4B–4E and S1B–S1E to S8B–S8E Figs). Even though the trajectories appear less stochastic when larger host populations are considered, because all scenarios assume a small number of infective persons in the early phase, there remains substantial stochasticity during the period of initial take-off which influences the timing of the epidemic peak.

We note that the performance of I.Poi (in terms of estimating *R*_0_ and *R*_*eff*_) deteriorates as more observations accumulate (Fig 4B and 4C and S1B–S1C to S8B –S8C Figs. A potential explanation of this observation is that I.Poi uses the mean of 1000 stochastic simulations which is likely to be close to the deterministic solution. If the observed stochastic trajectory peaks early or late, each additional observation will deviate from the expected behavior (for the true parameter) and, therefore, the precision worsens.

In the analyses presented so far, we assumed that the model structure is correct and that the calibration target (i.e. weekly number of diagnoses) can be accurately measured. To test the robustness of the results to imperfect calibration targets, we allowed for accumulating observations to be disturbed by an error term that follows a Gaussian distribution with mean zero and standard deviation 100. In this sensitivity analysis, the MSS method demonstrated the ability to cope with noisy data and sustain its performance (S9 Fig). We next considered a scenario for which the epidemic trajectories are provided by an SEITR model (see S1 Text subsection 2 “Equations of the SEITR model”) but an SITR model is chosen for parameter estimation and prediction. This allowed us to test to what extent model misspecification could erode the performance of these approaches, which is important because the true epidemic process may not be known. This sensitivity analysis revealed that each of the calibration methods considered here maintained their performance under this particular model misspecification scenario with respect to most performance criteria, but had problems with estimates for *R*_eff_ (S10 and S11 Figs). Each method failed to accurately estimate *R*_eff_ after the peak, which may be explained by the fact that in extreme epidemic scenarios used in this experiment, only a few number of individuals remain in the Susceptible compartment. Since the misspecified model used for calibration does not include the additional Exposed compartment, the size of Susceptible compartment is overestimated by all calibration methods, which leads to inaccurate *R*_eff_ estimates after the peak.

### Performance evaluation: Prediction

The F–I panels of Fig 4 and S1 to S8 Figs display the median of integrated relative errors (defined in Eq (16)) in predicting the number of diagnoses during the subsequent week, three weeks from now, accumulated over the next three weeks, and accumulated until the end of the epidemic from applying calibration methods described above on 50 simulated trajectories shown their A panels.

In all 9 scenarios (defined by epidemic severity and population size) and 5 epidemic times (8 and 4 weeks to peak, at the peak, and 4 and 8 weeks after the peak), the MSS method demonstrates superior or similar performance against the competing benchmark methods. As for the estimation targets, benchmark methods show higher integrated relative errors during both early and late phases of epidemics. This behavior occurs due to the small number of infectious individuals (which results in a low number of diagnoses) during these epidemic phases. The state updating procedure employed by the MSS method plays a central role in sustaining the performance of MSS method under these conditions.

**Fig 6:**
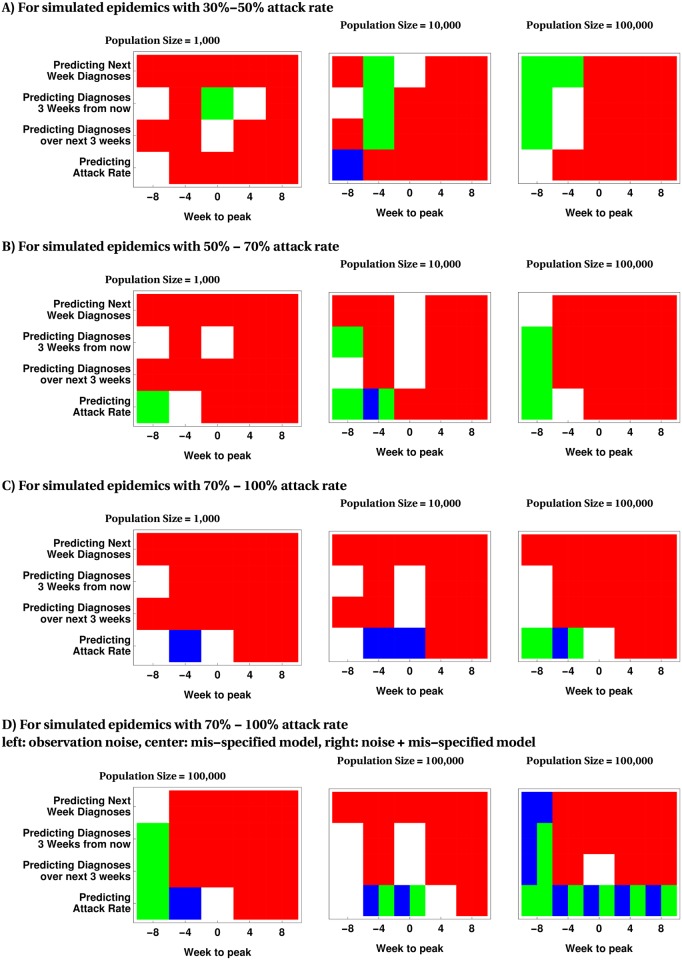
Identifying the calibration method with statistically dominant performance for predictions at 0.05 significance level. Red, if MSS is statistically better than all benchmark methods. White, if all methods fail to demonstrate statistically dominant performance. Blue if I.Poi (benchmark A), Green if Particle Filter (benchmark B) and Black if the Ensemble Kalman filter (benchmark C) statistically outperform the MSS method. Multiple colors can occur if more than one method is significantly better than MSS.

Fig 6A–6C displays the results of testing the hypothesis that the MSS method outperforms all benchmark methods at 0.05 significance level at different epidemic times. This figure suggest that in the majority of cases the MSS method demonstrates statistically superior than or similar performance to the benchmark methods (represented by Green, Blue and Black colors). We note that even for case where MSS is statistically dominated by another benchmark method, the absolute difference in mIRE (as shown in Fig 4 and S1 to S8 Figs) is not substantial.

The prediction ability of all methods is not strongly influenced by a slight model mis-specification as shwon in S10 Fig, but, importantly, the superiority holds as well despite lack of knowledge of the exact model.

## Discussion

Our results suggest that the MSS calibration method can address several major challenges inherent in parameter estimation and model-based prediction during outbreaks, when the true epidemic state can be only partially observed and the behavior of disease spread is most stochastic. Using a comprehensive set of simulation studies, we compare the performance of our method to several existing state-of-the-art calibration methods including a particle filter (PF) [27], an ensemble Kalman filter (EnKF) [27], and a likelihood approximation with the assumption of independent Poisson observations (I.Poi) [23]. For the majority of epidemic scenarios we evaluated, we find that MSS either outperforms or does as well as existing methods in terms of the ability to provide accurate estimates for the basic reproductive number (*R*_0_), effective *R*, mean duration of infectiousness, unobserved number of infected individuals, the epidemic final attack rate and the number of cases during future weeks.

The superior performance of MSS can be attributed to the use of LNA to capture the correlations between epidemic compartments throughout the simulated outbreak, and to the state updating procedure employed by MSS. While it would be in principal possible to incorporate an LNA approximation into a PF framework, we used the version of PF that is commonly used [27] for the purpose of this comparison study. The main difference between EnKF and MSS is that EnKF does not use an approximation for the likelihood for an observation but rather updates states and parameters based on correlation between compartments and data using multiple parameter vectors. The MSS state updating procedure allows the calculation of these correlations using a single parameter vector. This results in a more accurate description of correlations between compartments as epidemic data are accumulating.

The LNA has been successfully applied to describe intrinsic stochastic fluctuations in various contexts [44–46] and has a solid theoretical basis [47, 48]. The LNA provides accurate approximation for large populations or when the fluctuations are small compared to the steady state of the system. As also suggested by the results presented here, LNA can perform well even when the system is not in steady state as long as the intervals over which the LNA projections are made are sufficiently small (here, observations occurred at weekly time steps for epidemics that lasted up to 100 weeks).

A main advantage of the MSS technique is that the LNA needs only to be employed on the relatively short time intervals between recordings and not over the whole course of the epidemics. This is possible by employing the state estimation procedure to initialize the LNA on the succeeding interval. Additionally, the simulation studies presented here and previous work in a Systems Biology context [35, 36] show that LNA can yield accurate estimates for parameters for stochastic systems. Nevertheless, if required, LNA accuracy can be improved by using higher order terms for correction [49]. For additional discussion on the LNA in a calibration setting (LNA on a whole system) see [50], for additional theoretical remarks [48] or the original work [47].

The computationally challenging part of the MSS is the interval-wise evaluation of the LNA which consists of *n*_*c*_ + *n*_*c*_ (*n*_*c*_ + 1)/2 ODEs where *n*_*c*_ stands for the number of compartments. PF and EnKF, on the other hand, only require the solution of the ODE system that consists of *n*_*c*_ equations. The I.Poi. requires the execution of stochastic simulations for each parameter and is the most cost intensive of all four alternatives tested. MSS can be easily carried out without the use of a computing cluster: calibration and prediction for one time-series at the peak of the epidemic takes roughly 5 minutes on an Intel Xeon CPU E5-2630 v3 with 16GM RAM, see the S1 Text subsection 4 “Computational Effort” for a comparison of runtimes.

We note that the performance of filtering methods can be impaired by filter degeneracy, a phenomenon where particles or members of an ensemble or filter have a very small likelihood value that varies over orders of magnitude even for the best members/particles. Our implementation of PF and EnKF is consistent with the original implementation by [27] and both methods are equipped with suitable mechanisms to handle filter degeneracy. Despite the fact that the MSS approach proposed here does not include such a mechanism to address filter degeneracy, MSS demonstrated very competitive performance in these simulated epidemic scenarios. We believe that speaks to the promise of MSS and potential extensions of this methodology.

The I.Poi method assumes that epidemic observations gathered over time are independent, an assumption that may be violated in many epidemic scenarios. For example, S12 Fig shows a clear correlation pattern among observations of epidemics trajectories produced by the SITR model of Fig 3. Despite lacking a mechanism to account for correlations between epidemic observations, I.Poi remained competative under several of the epidemic scenarios studied here.

Our numerical analysis suggests that the combination of noisy observations and model mis-specification will erode the performance of all calibration methods considered here S11 Fig. However, our results indicate that mis-specification of model structure far outweighs the impact of observational noise in these simulations (compare S8 and S9 Figs which show similar results fort he noise-free and noisy scenarios). This finding highlights the importance of selecting a model structure that adequately reflects the complexity of the disease.

The performance of PF and EnKF can be affected by the choice of observational variance (Eq 13). The observation variance we selected for our analyses is adapted from [27], which is a heuristic choice but has proved reliable in previous studies [26]. We ran a sensitivity analysis on this parameter by dropping the absolute variance term from 10,000 to 1, and observed no major change in the performance of PF and EnKF methods in populations of various size. This suggests that the absolute term in the observational variance is not the determining factor in the relative performance of EnKF, PF and MSS.

We note four main limitations of our study. First, while including the necessary complexity to offer a suitable platform for our analyses, the epidemic model used to evaluate the comparative performance of calibration methods is highly simplified. Investigating the impact of model complexity level on the performance of calibration methods would be of immediate interest for future research. Second, our evaluation are based on “simulated” epidemic trajectories. While only simulated scenarios can allow us to calculate the relative errors in estimating the unknown parameters (e.g. *R*_0_, mean duration of infectiousness), further investigation using real-world data is required to comprehensively evaluate the performance of MSS along with other existing calibration methods.

Third, in our analyses, we used an SITR model of influenza epidemics that allows for a delay between the time of infection and time of diagnosis. Our model, however, does not account for the fact that in reality only a portion of influenza cases will be reported as not all infected individuals will have severe enough symptoms to seek care. If the fraction of cases that remains undetected is known and fixed, this is easily handled in the model with the addition of compartments and parameters to reflect the observation process, However, the degree of underreporting is often unknown and may change over the course of an epidemic. Such underreporting impacts the performance of all available calibration methods, and the development of novel approaches that can better account for this phenomenon is an important direction for future research.

And finally, our simulation study assumed that epidemic parameters, such as contact rate, remain constant through the outbreak. In reality, however, some epidemic parameters may vary over time due to changes in population behavior and interactions. We also note that for simplicity, our analysis used only one type of real-time observations (i.e. weekly diagnoses), but the methods presented here can be extended for scenarios where multiple sources of real-time data (e.g. disease-related hospitalizations and deaths) are available.

In summary, precise and timely estimation of key epidemic parameters (e.g. expected number of secondary cases, or mean duration of infectiousness) remains a critical component of accurate model-based prediction and effective response to infectious disease outbreaks. The increase in accuracy offered by the MSS method could enable public health officials to response more effectively to epidemic threats.

## Supporting Information

S1 Text (PDF)

S1 Fig Median integrated relative errors (mIRE) in estimating model parameters and infection prevalence as well as prediction for simulated epidemics in a population of 1 000 with 30% − 50% attack rate. Same setting as in Fig 4.
(TIF)

S2 Fig Median integrated relative errors (mIRE) in estimating model parameters and infection prevalence as well as prediction for simulated epidemics in a population of 10 000 with 30% − 50% attack rate. Same setting as in Fig 4.
(TIF)

S3 Fig Median integrated relative errors (mIRE) in estimating model parameters and infection prevalence as well as prediction for simulated epidemics in a population of 100 000 with 30% − 50% attack rate. Same setting as in Fig 4.
(TIF)

S4 Fig Median integrated relative errors (mIRE) in estimating model parameters and infection prevalence as well as prediction for simulated epidemics in a population of 1 000 with 50% − 70% attack rate. Same setting as in Fig 4.
(TIF)

S5 Fig Median integrated relative errors (mIRE) in estimating model parameters and infection prevalence as well as prediction for simulated epidemics in a population of 100 000 with 50% − 70% attack rate. Same setting as in Fig 4.
(TIF)

S6 Fig Median integrated relative errors (mIRE) in estimating model parameters and infection prevalence as well as prediction for simulated epidemics in a population of 1 000 with 70% − 100% attack rate. Same setting as in Fig 4.
(TIF)

S7 Fig Median integrated relative errors (mIRE) in estimating model parameters and infection prevalence as well as prediction for simulated epidemics in a population of 10 000 with 70% − 100% attack rate. Same setting as in Fig 4.
(TIF)

S8 Fig Median integrated relative errors (mIRE) in estimating model parameters and infection prevalence as well as prediction for simulated epidemics in a population of 100 000 with 70% − 100% attack rate. Same setting as in Fig 4.
(TIF)

S9 Fig Median integrated relative errors (mIRE) in estimating model parameters and infection prevalence as well as prediction for simulated epidemics in a population of 100 000 with 70% − 100% attack rate and additive observation noise. Same setting as in Fig 4.
(TIF)

S10 Fig Median integrated relative errors (mIRE) in estimating model parameters and infection prevalence as well as prediction for simulated epidemics in a population of 100 000 with 70% − 100% attack rate with a mis-specified model. Same setting as in Fig 4.
(TIF)

S11 Fig Median integrated relative errors (mIRE) in estimating model parameters and infection prevalence as well as prediction for simulated epidemics in a population of 100 000 with 70% − 100% attack rate with a mis-specified model and additive observation noise. Same setting as in Fig 4.
(TIF)

S12 Fig Correlation structure of the SITR model: Inter-temporal correlation for new cases in SITR model, calculated from the 50 stochastic simulations shown in Fig 4A. (TIF)

S1 File Mathematica Code of the method and all simulation studies. (TAR.GZ)

## References

[1] Lipsitch M, Finelli L, Heffernan RT, Leung GM, Redd; for the 2009 H1N1 Surveillance Group SC. Improving the evidence base for decision making during a pandemic: The example of 2009 influenza A/H1 N1. Biosecurity and bioterrorism: biodefense strategy, practice, and science. 2011;9(2):89–115. 10.1089/bsp.2011.0007 PMC3102310 21612363

[2] Mills CE, Robins JM, Lipsitch M. Transmissibility of 1918 pandemic influenza. Nature. 2004;432(7019):904–906. 10.1038/nature03063 15602562 PMC7095078

[3] Cauchemez S, Donnelly CA, Reed C, Ghani AC, Fraser C, Kent CK, et al Household transmission of 2009 pandemic influenza A (H1N1) virus in the United States. New England Journal of Medicine. 2009;361(27):2619–2627. 10.1056/NEJMoa0905498 20042753 PMC3840270

[4] Cowling BJ, Lau MS, Ho LM, Chuang SK, Tsang T, Liu SH, et al The effective reproduction number of pandemic influenza: prospective estimation. Epidemiology. 2010;21(6):842 10.1097/EDE.0b013e3181f20977 20805752 PMC3084966

[5] Cori A, Ferguson NM, Fraser C, Cauchemez S. A new framework and software to estimate time-varying reproduction numbers during epidemics. American journal of epidemiology. 2013;178(9):1505–1512. 10.1093/aje/kwt133 24043437 PMC3816335

[6] Cauchemez S, Epperson S, Biggerstaff M, Swerdlow D, Finelli L, Ferguson NM. Using routine surveillance data to estimate the epidemic potential of emerging zoonoses: application to the emergence of US swine origin influenza A H3N2v virus. PLoS Med. 2013;10(3):e1001399 10.1371/journal.pmed.1001399 23472057 PMC3589342

[7] Cauchemez S, Carrat F, Viboud C, Valleron A, Boelle P. A Bayesian MCMC approach to study transmission of influenza: application to household longitudinal data. Statistics in medicine. 2004;23(22):3469–3487. 10.1002/sim.1912 15505892

[8] Höhle M, Jørgensen E, O’Neill PD. Inference in disease transmission experiments by using stochastic epidemic models. Journal of the Royal Statistical Society: Series C (Applied Statistics). 2005;54(2):349–366. 10.1111/j.1467-9876.2005.00488.x

[9] White LF, Pagano M. A likelihood-based method for real-time estimation of the serial interval and reproductive number of an epidemic. Stat Med. 2008 7;27(16):2999–3016. 10.1002/sim.3136 18058829 PMC3951165

[10] O’Neill PD. A tutorial introduction to Bayesian inference for stochastic epidemic models using Markov chain Monte Carlo methods. Mathematical biosciences. 2002;180(1):103–114. 12387918 10.1016/s0025-5564(02)00109-8

[11] Obadia T, Haneef R, Boelle PY. The R0 package: a toolbox to estimate reproduction numbers for epidemic outbreaks. BMC Med Inform Decis Mak. 2012;12:147 10.1186/1472-6947-12-147 23249562 PMC3582628

[12] Cauchemez S, Boelle PY, Donnelly CA, Ferguson NM, Thomas G, Leung GM, et al Real-time estimates in early detection of SARS. Emerging Infect Dis. 2006 1;12(1):110–113. 10.3201/eid1201.050593 16494726 PMC3293464

[13] Cauchemez S, Boëlle PY, Thomas G, Valleron AJ. Estimating in real time the efficacy of measures to control emerging communicable diseases. American Journal of Epidemiology. 2006;164(6):591–597. 10.1093/aje/kwj274 16887892

[14] Wallinga J, Teunis P. Different epidemic curves for severe acute respiratory syndrome reveal similar impacts of control measures. American Journal of Epidemiology. 2004;160(6):509–516. 10.1093/aje/kwh255 15353409 PMC7110200

[15] Davoudi B, Miller JC, Meza R, Meyers LA, Earn DJD, Pourbohloul B. Early real-time estimation of the basic reproduction number of emerging infectious diseases. Physical Review X. 2012;2 10.1103/PhysRevX.2.031005

[16] Cauchemez S, Ferguson NM. Methods to infer transmission risk factors in complex outbreak data. J R Soc Interface. 2012 3;9(68):456–469. 10.1098/rsif.2011.0379 21831890 PMC3262428

[17] Saramaki J, Kaski K. Modelling development of epidemics with dynamic small-world networks. J Theor Biol. 2005 6;234(3):413–421. 10.1016/j.jtbi.2004.12.003 15784275

[18] Anderson RM, May RM. Infectious Diseases of Humans: Dynamics and Control. Oxford University Press; 1992.

[19] Daley DJ, Gani JM. Epidemic Modelling: An Introduction. Cambridge; New York: Cambridge University Press; 1999.

[20] Alkema L, Raftery AE, Clark SJ. Probabilistic projections of HIV prevalence using Bayesian melding. The Annals of Applied Statistics. 2007;p. 229–248. 10.1214/07-AOAS111

[21] Elderd BD, Dukic VM, Dwyer G. Uncertainty in predictions of disease spread and public health responses to bioterrorism and emerging diseases. Proceedings of the National Academy of Sciences.2006;103(42):15693–15697. 10.1073/pnas.0600816103 PMC1592533 17030819

[22] Birrell PJ, Ketsetzis G, Gay NJ, Cooper BS, Presanis AM, Harris RJ, et al Bayesian modeling to unmask and predict influenza A/H1N1pdm dynamics in London. Proceedings of the National Academy of Sciences.2011;108(45):18238–18243. 10.1073/pnas.1103002108 PMC3215054 22042838

[23] Riley S, Fraser C, Donnelly CA, Ghani AC, Abu-Raddad LJ, Hedley AJ, et al Transmission dynamics of the etiological agent of SARS in Hong Kong: impact of public health interventions. Science. 2003;300(5627):1961–1966. 10.1126/science.1086478 12766206

[24] Choi B, Rempala GA. Inference for discretely observed stochastic kinetic networks with applications to epidemic modeling. Biostatistics. 2012 1;13(1):153–165. 10.1093/biostatistics/kxr019 21835814 PMC3276272

[25] Ionides EL, Breto C, King AA. Inference for nonlinear dynamical systems. Proc Natl Acad Sci USA. 2006 12;103(49):18438–18443. 10.1073/pnas.0603181103 17121996 PMC3020138

[26] Shaman J, Karspeck A, Yang W, Tamerius J, Lipsitch M. Real-time influenza forecasts during the 2012–2013 season. Nat Commun. 2013;4:2837 10.1038/ncomms3837 24302074 PMC3873365

[27] Yang W, Karspeck A, Shaman J. Comparison of Filtering Methods for the Modeling and Retrospective Forecasting of Influenza Epidemics. PLOS Computational Biology. 2014;10:e1003583 10.1371/journal.pcbi.1003583 24762780 PMC3998879

[28] Yang W, Cowling BJ, Lau EH, Shaman J. Forecasting Influenza Epidemics in Hong Kong. PLoS Comput Biol. 2015 7;11(7):e1004383 10.1371/journal.pcbi.1004383 26226185 PMC4520691

[29] Shaman J, Karspeck A. Forecasting seasonal outbreaks of influenza. Proc Natl Acad Sci USA. 2012 12;109(50):20425–20430. 10.1073/pnas.1208772109 23184969 PMC3528592

[30] Ong JB, Chen MI, Cook AR, Lee HC, Lee VJ, Lin RT, et al Real-time epidemic monitoring and forecasting of H1N1-2009 using influenza-like illness from general practice and family doctor clinics in Singapore. PLoS ONE. 2010;5(4):e10036 10.1371/journal.pone.0010036 20418945 PMC2854682

[31] Dukic V, Lopes HF, Polson NG. Tracking Epidemics With Google Flu Trends Data and a State-Space SEIR Model. Journal of the American Statistical Association. 2012;107 10.1080/01621459.2012.713876 PMC10426794 37583443

[32] Chretien JP, George D, Shaman J, Chitale RA, McKenzie FE. Influenza forecasting in human populations: a scoping review. PLoS ONE. 2014;9(4):e94130 10.1371/journal.pone.0094130 24714027 PMC3979760

[33] Bettencourt LM, Ribeiro RM. Real time Bayesian estimation of the epidemic potential of emerging infectious diseases. PLoS One. 2008;3(5):e2185 10.1371/journal.pone.0002185 18478118 PMC2366072

[34] Abbey H. An examination of the Reed-Frost theory of epidemics. Human biology. 1952;24(3):201–233. 12990130

[35] Zimmer C, Sahle S. Deterministic inference for stochastic systems using multiple shooting and a linear noise approximation for the transition probabilities. IET Systems Biology. 2015;9:181–192. 10.1049/iet-syb.2014.0020 26405142 PMC8687418

[36] Zimmer C. Reconstructing the hidden states in time course data of stochastic models. Mathematical BioSciences. 2015;269:117–129. 10.1016/j.mbs.2015.08.015 26363082

[37] Thomas P, Matuschek H, Grima R. Intrinsic noise analyzer: a software package for the exploration of stochastic biochemical kinetics using the system size expansion. PloS one. 2012;7(6):e38518 10.1371/journal.pone.0038518 22723865 PMC3373587

[38] Van Kampen NG. Stochastic processes in physics and chemistry. vol. 1 Elsevier; 1992.

[39] Yaesoubi R, Cohen T. Generalized Markov models of infectious disease spread: A novel framework for developing dynamic health policies. European Journal of Operational Research. 2011;215:679–687. 10.1016/j.ejor.2011.07.016 21966083 PMC3182455

[40] Gillespie DT. A General Method for Numerically Simulating the Stochastic Time Evolution of coupled Chemical Reactions. Journal of Computational Physics. 1976;22 (4):403–434. 10.1016/0021-9991(76)90041-3

[41] Influenza prediction challenge. Center for disease control and prevention.2016; http://www.cdc.gov/flu/news/flu-forecast-website-launched.htm.

[42] Hoops S, Sahle S, Gauges R, Lee C, Pahle J, Simus N, et al COPASI—a COmplex PAthway SImulator. Bioinformatics. 2006;22 (24):3067–3074. 10.1093/bioinformatics/btl485 17032683

[43] Mathematica, Version 10.4 Wolfram Research, Inc 2015;Champaign, IL.

[44] Pahle J, Challenger JD, Mendes P, McKane AJ. Biochemical fluctuations, optimisation and the linear noise approximation. BMC Systems Biology. 2012;6 10.1186/1752-0509-6-86 22805626 PMC3814289

[45] Challenger JD, McKane AJ, Pahle J. Multi-compartment linear noise approximation. Journal of Statistical Mechanics: Theory and Experiment. 2012;P11010 10.1088/1742-5468/2012/11/P11010

[46] Straube R, von Kamp A. LiNA—A Graphical Matlab Tool for Analyzing Intrinsic Noise in Biochemical Reaction Networks. online tutorial. 2013; http://www2.mpi-magdeburg.mpg.de/projects/LiNA/Tutorial_LiNA_v1.pdf.

[47] van Kampen NG. Stochastic processes in physics and chemistry. Elsevier; 2007.

[48] Grima R. An effective rate equation approach to reaction kinetics in small volumes: Theory and application to biochemical reactions in nonequilibrium steady-state conditions. The Journal of Chemical Physics. 2010;133:035101 10.1063/1.3454685 20649359

[49] Thomas P, Matuschek H, Grima R. Intrinsic Noise Analyzer: A Software Package for the Exploration of Stochastic Biochemical Kinetics Using the System Size Expansion. Plos ONE. 2012;7:e38518 10.1371/journal.pone.0038518 22723865 PMC3373587

[50] Komorowski M, Finkenstädt B, Harper CV, Rand DA. Bayesian inference of biochemical kinetic parameters using the linear noise approximation. BMC Bioinformatics. 2009;10:343 10.1186/1471-2105-10-343 19840370 PMC2774326

