# Supplementary material for: A Likelihood Approach for Real-Time Calibration of Stochastic Compartmental Epidemic Models
Source: PLoS Comput Biol. 2017 Jan 17;13(1):e1005257. doi: 10.1371/journal.pcbi.1005257 (PMC5240920; doi:10.1371/journal.pcbi.1005257)
Supplement: S1 Text — (PDF) [file pcbi.1005257.s001.pdf]

S1 Text – Supporting text

S1.1 Text

The Gillespie Algorithm

In the section "Predicting epidemic behavior", we discussed that to use Eq. (11) to project the expected behavior of an epidemic outcome Z using the accrued observation Y_i at time t_i, one needs access to a stochastic simulator to sample from the random variable $Z|\nu_i$ for a given set of parameter values θ. We use the Gillespie algorithm [40] for this purpose. To describe the steps of the Gillespie algorithm, we consider an epidemic model with K compartments (e.g. in the model described in § "Design of the Performance Analysis", $K = 4$). Let $\nu(t) = (\nu^1(t), \nu^2(t), \ldots, \nu^K(t))$ denote the state of the epidemic at time t, where $\nu^k(t)$ denotes the number of individuals in compartment $k \in \{1, 2, \ldots, K\}$ at time t.

The state of an epidemic (i.e. $\nu(t)$) may change due to the occurrence of specific events, such the transmission of the disease to a susceptible or the recovery from the disease. We use Q to denote the number of such events. In the model shown in Fig 3, these epidemic events include Infection, Seeking Treatment, and Recovery (and hence $Q = 3$). Let $\Lambda_q(\nu(t), \theta)$ denote the rate at which the event q is occurring when the epidemic is at state $\nu(t)$ and with parameter values θ that is a transformation of the parameter θ.

Note that the stochastic model usually uses a volume dependent transformation of the parameter θ from the ODE model. We do not need this transformation as our ODE description is volume independent.

In the model described in §– "Design of the Performance Analysis", the force of infection is $\theta_1 \nu^S(t) \frac{\nu^I(t)}{N(t)}$, the rate of seeking treatment is $\theta_2 \nu^I(t)$, and the rate of recovery is $\theta_3 \nu^T(t)$, where $\nu^S(t)$, $\nu^I(t)$ and $\nu^T(t)$ denote, respectively, the number of individuals in the compartments Susceptible, Infective, and Treatment (see Fig 3). The Gillespie algorithm proceeds as follows.

Assuming that the epidemic is at state $v(t)$ at time t :

1. Calculate the sum of rates: $\Lambda_0 = \sum_{r=1}^{Q} \Lambda_q(\nu(t), \theta)$.
2. Sample the time to the next event: $\tau \leftarrow -\frac{1}{\Lambda_0} \ln u_1$, where u_1 is a random number drawn from a uniform distribution $\mathcal{U}((0, 1])$.
3. Determine the event that just realized: Select the event $\tilde{q}$ such that

$$\sum_{q=1}^{\tilde{q}-1} \frac{\Lambda_q(\nu(t), \theta)}{\Lambda_0} < u_2 \leq \sum_{q=\tilde{q}}^{Q} \frac{\Lambda_q(\nu(t), \theta)}{\Lambda_0}$$

   where u_2 is another random number drawn from a uniform distribution $\mathcal{U}((0, 1])$.
4. Update epidemic state given the realized event $\tilde{r}$.
5. Until a desired time condition is satisfied, increment time $t \leftarrow t + \tau$ and move to Step 1.

Figure SI 1. Pseudo-code of Gillespie algorithm for simulating stochastic compartmental models.

To obtain simulation trajectories displayed in Fig 4 A and Figs S1 Fig A to S8 Fig A,

we use the Gillespie algorithm (Fig SI 1) to simulate the SITR compartmental model shown in Fig 1.

These SITR trajectories are used to calculate the number of new diagnoses in week i which is equal to $T_i + R_i - T_{i-1} - R_{i-1}$. If for a simulated trajectory the conditions on attack rate (30% - 50% for the mild scenario, 50%-70% for the severe scenario and 70%-100% for the extreme scenario) and timing of peak (between 10 and 20) are fulfilled, we keep the trajectory for the respective scenarios or we otherwise we reject it.

S1.2 Text

Equations of the SEITR model

The Susceptible - Exposed - Infected - Treatment - Recovered model contains one more compartment than the SITR model, namely Exposed. Disease transmission can be modeled using the following ODE model:

$$\begin{aligned}
\frac{d}{dt}x_S(t) &= -k_1 x_S(t)\frac{x_I(t)}{N(t)}, \\
\frac{d}{dt}x_E(t) &= k_1 x_S(t)\frac{x_I(t)}{N(t)} - k_2 x_E(t), \\
\frac{d}{dt}x_I(t) &= k_2 x_E(t) - \theta_2 x_I(t), \\
\frac{d}{dt}x_T(t) &= \theta_2 x_I(t) - \theta_3 x_t(t), \\
\frac{d}{dt}x_R(t) &= \theta_3 x_T(t)
\end{aligned} \tag{17}$$

where $k_1 = k_2 = 2\theta_1$ and θ_1 is the disease transmission rate, θ_2 is the rate of seeking treatment while infectious, and θ_3 is the rate of recovering.

In our analysis, we assume that at time $t = 0$ one population member becomes infected. The model initial condition can therefore be defined as $x_0 = (x_S(0), x_E(0), x_I(0), x_T(0), x_R(0)) = (N-1, 1, 0, 0)$. We furthermore assume a constant population size $N(t) = x_S(t) + x_E(S) + x_I(t) + x_T(t) + x_R(t)$ denote the population size at time t .

S1.3 text

Formulas to calculate integrated relative errors (IRE) for different metrics

The exact form of $f_{M_i|\theta}(m|\theta)$ of the IREs in Eq. (16) depends on the metric of interest.

R_0 and duration of infectiousness: For R_0 or the mean duration of infectiousness, we have $f_{M_i|\theta}(m|\theta) = 1$, as R_0 and the mean duration of infectiousness only depend on the parameter vector sampled from the posterior π_i.

R_{eff}: For R_{eff} as the performance target, M_i is defined as $M_i = R_0 \frac{S_i}{N}$. As S is the only state that the target depends on, the function $f_{M_i|\theta}(m|\theta)$ needs to yield the probability for this state. This is accomplished by integrating over the remaining states:

$$f_{M_i|\theta}(m|\theta) = \int_{\left(\nu_i^{(S)},\nu_i^{(I)},\nu_i^{(T)},\nu_i^{(R)}\right)\in\Omega_i} \Pi_i\left(\left(\nu_i^{(I)},\nu_i^{(T)},\nu_i^{(R)}\right)|Y_i;\theta\right)$$

Infection prevalence: If the target is the infection prevalence: $M_i = \nu_i^{(S)}$, the function $f_{M_i|\theta}(m|\theta)$ needs to yield the probability for this state and, hence, is calculated by integrating over the remaining states.

$$f_{\nu_i^{(S)}|\theta}(\nu_i^{(I)}|\theta) = \int_{\left(\nu_i^{(S)},\nu_i^{(T)},\nu_i^{(R)}\right)\in\Omega_i} \Pi_i\left(\left(\nu_i^{(S)},\nu_i^{(T)},\nu_i^{(R)}\right)|Y_i;\theta\right)$$

As the I.Poi benchmark does not calculate a belief state for time t_i, we use the simulation model to carry out simulations with parameters according to $\pi_i(\theta|Y_i)$ and consider these simulations as a sample from the belief state Π_i.

All Predictions: In case of prediction for one week, three weeks (cumulative and specific) or the attack rate, it holds

$$f_{M_i|\theta}(m|\theta) = P(m|Y_i)$$

with P as in Eq. (9).

Median integrated relative error (mIRE): Next, we will calculate a median relative error (mIRE) over the 50 trajectories as

$$mIRE(M_i) = \text{median}\left(IRE(M_i^{(1)}),\ldots,IRE(M_i^{(50)})\right).$$

S1.4 text

Computational Effort

As mentioned in the Discussion, the most computationally challenging parts are the ODE system integration for MSS, PF and EnKF and the stochastic simulation for I. Poi. We analyzed the computational time for the severe scenario with a population size of 10000. Carrying out the 1000 stochastic simulations for benchmark I.Poi for the 1000 parameters takes about 100 minutes. The additional time for evaluating the likelihood approximation is rather short with about only 1 second, see table 1 for numbers.

The computational times are shorter before the peak and longer after the peak, when less or more data is taken into account. The computational time will only vary for

the I.Poi. when changing the population size as the other methods do not require stochastic simulations.

We note that the implementation was not carried out in a way to maximize speed, so there is opportunity for additional speed up with more efficient implementation. However, these numbers demonstrate that all the algorithms can be used on a personal computer in real time.

Table 1. Computational time for MSS and benchmarks:

time to peak	MSS	I.Poi.	PF	EnKF
8 weeks to peak	85.2 ± 32.8	100* min + 0.6 ± 0.3	7.7± 4.1	10.6± 5.9
4 weeks to peak	125.2± 33.2	100* min + 1 ± 0.3	12.5± 4.1	17.8± 6.
at peak	157.6± 31.6	100* min + 1.4 ± 0.3	17.4± 4.	26.4± 6.3
4 weeks after peak	196.2± 33.8	100* min + 1.7± 0.3	22.6± 4.	34.5± 6.6
8 weeks after peak	228.8± 35.	100* min + 2.1± 0.3	27.5± 4.1	41.8± 6.3

Computational time in seconds for the calibration of one time course in average for the severe scenario in the population with 10000 individuals.
* The 100 min are the computational time to carried out 1000 stochastic simulations for each parameter with the Gillespie algorithm.

S1.5 text

Pseudo code for the benchmark method A (I.Poi)

1. Initialization
   (a) Choose an initial prior probability function $\pi_0(\theta)$.
   (b) Choose the set Θ that includes the values of parameters θ for which the approximate likelihood function (10) should be calculated.
   (c) Set $\ln L^{(\text{I.Poi})}(Y_0;\theta) \leftarrow 0$ for every $\theta \in \Theta$.
2. Calibration: For each observation y_i , $i = 1, \ldots, n$,
   (a) Assume $\mathcal{P}^{(\text{I.Poi})}(\cdot|\theta) \sim Poisson(\mu_i)$ and use simulated trajectories to estimate the mean μ_i, for every $\theta \in \Theta$.
   (b) Update the likelihood function: $L^{(\text{I.Poi})}(Y_i;\theta) \leftarrow L^{(\text{I.Poi})}(Y_{i-1};\theta) \times \mathcal{P}^{(\text{I.Poi})}(y_i|\theta)$ for every $\theta \in \Theta$.
   (c) Update the parameter posterior distribution: $\pi_i(\theta|Y_i) \leftarrow L^{(\text{I.Poi})}(y_i|\theta)\ \pi_{i-1}(\theta, Y_{i-1})$ for every $\theta \in \Theta$.

Figure SI 2. Pseudo code for the benchmark method A (I.Poi) [23].

S1.6 text

Pseudo code for the Particle Filter

1. Initialization
   (a) Choose an initial prior probability function $\pi_0(\theta)$.
   (b) Choose the set Θ that includes the values of parameters θ for which the likelihood function (1) should be calculated with the specifications in Eq. (12) and name the specific likelihood approximation $L^{(\mathrm{PF})}$.
   (c) Set $\ln L^{(\mathrm{PF})}(Y_0;\theta) \leftarrow 0$ for every $\theta \in \Theta$.
2. Calibration: For each observation y_i , $i = 1, \ldots, n$,
   (a) Calculate the probability $\mathcal{P}^{(\mathrm{PF})}(y_i|Y_{i-1};\theta)$ for every $\theta \in \Theta$.
   (b) Update the likelihood function: $L^{(\mathrm{PF})}(Y_i;\theta) \leftarrow L^{(\mathrm{PF})}(Y_{i-1};\theta) \times \mathcal{P}^{(\mathrm{PF})}(y_i|Y_{i-1};\theta)$ for every $\theta \in \Theta$.
   (c) Update the parameter posterior distribution: $\pi_i(\theta|Y_i) \leftarrow L^{(\mathrm{PF})}(y_i|\theta)\ \pi_{i-1}(\theta, Y_{i-1})$ for every $\theta \in \Theta$.

Figure SI 3. Pseudo code for the Particle Filter [27]

S1.7 text

Pseudo code for the Ensemble Kalmann filter

1. Initialization
   (a) Choose an initial prior probability function $\pi_0(\theta)$.
   (b) Choose the set Θ that includes the values of parameters θ for which the likelihood function (1) should be calculated with the specifications in Eq. (12).
   (c) Choose an initial set of particles Ψ. Its elements $\psi \in \Psi$ contain values for the parameters and states: $\psi = (\theta, \nu)$.
2. Calibration: For each observation $y_i, i = 1, \ldots, n$,
   (a) Propagate each particle by setting $\psi \leftarrow (\theta, \nu_{prior})$ with $\nu_{prior} = x(t_i - t_{i-1}, \nu; \theta)$.
   (b) Calculate the prior for the observations: $\psi^{(y)} = \nu^{(T)}_{prior} + \nu^{(R)}_{prior} - \nu^{(T)} - \nu^{(R)}$.
   (c) Calculate the variance $\sigma^2_{obs,i}$ as in Eq. (13).
   (d) Calculate the prior variance of the observed quantity as $\sigma^2_{prior} = \text{Variance}\left(\psi^{(y)}\right)$.
   (e) Calculate the prior co-variance of the observed quantity and each unobserved component of the particle ψ as $\sigma_m = \text{co-variance}\left(\psi^{(y)}, \psi^{(m)}\right)$, where m denotes the unobserved component.
   (f) Update $\psi \leftarrow \frac{\sigma_m}{\sigma^2_{prior}} \delta$ with
   $$\delta = \frac{\sigma^2_{obs}}{\sigma^2_{obs} + \sigma^2_{prior}} \psi^y_{prior} + \frac{\sigma^2_{prior}}{\sigma^2_{obs} + \sigma^2_{prior}} \tilde{y}_i$$
   and $\tilde{y}_i = y_i + \xi_i$, with $\xi_i \sim N(0, \sigma^2_{obs,i})$:
   (g) Set $(\theta, \nu) \leftarrow \psi$ for the next iteration.

Figure SI 4. Pseudo code for the Ensemble Kalmann filter [27].

S1.8 text

Detailed pseudo code for our MSS for SITR

1. **Input:**
   - ODE solution $x(t, x_0; \theta)$ for an ODE x' integrated for time t with initial value x_0 and parameter θ such as in equation (5).
   - Data set $y_1, \ldots, y_n$
   - Number of ensemble members N_{grid}.
   - Total populations size N.

2. **Initialization:**

FOR j=1,...,N_{grid}

$\theta^{(j)} = \left(R_0^{(j)}, mdi^{(j)}, \gamma^{(j)}\right) \sim U(R_0^{(\text{range})} \times mdi^{(\text{range})} \times \gamma^{(\text{range})})$

% Draw a parameter sample from an uninformative prior; mdi - mean duration of infectiousness;

% γ - additional parameter for the number of weeks, the epidemics started before the first observation

% in our case $R_0^{(range)} = [1,3]$, $mdi^{(range)} = [1,20]$, $\gamma^{(range)} = [0,5]$.

$S_{-\gamma}^{(j)} = N - 1$ *% initialize states*

$I_{-\gamma}^{(j)} = 1$ *% we assume one infected at start, but no knowledge when epidemic started*

$T_{-\gamma}^{(j)} = R_{-\gamma}^{(j)} = 0$

$\hat{\nu}_{-\gamma}^{(j)} = \left(S_{-\gamma}^{(j)}, I_{-\gamma}^{(j)}, T_{-\gamma}^{(j)}, R_{-\gamma}^{(j)}\right)$

$L(Y_{-\gamma}|\theta^{(j)}) = 1$

$y_{-\gamma} = y_{-\gamma+1} = \ldots = y_0 = 0$ % auxiliary observations to model the time before the first case

END FOR

3. **Loop:**

FOR $i = -\gamma, \ldots, n$ *% epidemic stared at $-\gamma$ and data available until n*

FOR $j = 1, \ldots, N_{grid}$

% % % Forward integration

$\tilde{\nu}_i^{(j)} = x(t_i - t_{i-1}, \hat{\nu}_{i-1}^{(j)}; \theta^{(j)})$ *% ODE integration*

$Cov_i^{(j)} = \Sigma(t_i - t_{i-1}, \hat{\nu}_{i-1}^{(j)}; \theta^{(j)})$ *% LNA co-variance integration*

$Cov_{i,(k,k)}^{(j)} = \max(Cov_{i,(k,k)}^{(j)}, \alpha)$ for $k \in \{S, I\}$ *% ensure that no negative co-variance, we use $\alpha = 10^{-6}$*

% % % % calculate state estimate in case of observation noise with standard deviation σ^2

% The state estimate is calculated by maximizing the probability in equation 7 .

% LNA $\Rightarrow$ p normal and $P(y_i|\nu_i, \hat{\nu}_{i-1}; \theta)$ normal as gaussian observation noise

% taking into account that $\hat{\nu}_i^{(j,T)} + \hat{\nu}_i^{(j,R)} - \hat{\nu}_{i-1}^{(j,T)} + \hat{\nu}_{i-1}^{(j,R)}$ would correspond to a noise free observation

% $\Rightarrow \max_{\hat{\nu}_i} \quad (2\pi)^{-\frac{n_{dim}}{2}} (det(Cov_i^{(j)}))^{-\frac{1}{2}} \times exp\left(-(\tilde{\nu}_i^{(j)} - \hat{\nu}_i^{(j)})(Cov_i^{(j)})^{-1}(\tilde{\nu}_i^{(j)} - \hat{\nu}_i^{(j)})\right)$

% $\frac{1}{\sqrt{2\pi\sigma^1}} exp\left(-\frac{\left(\left(\hat{\nu}_i^{(j,T)}+\hat{\nu}_i^{(j,R)}-\hat{\nu}_{i-1}^{(j,T)}+\hat{\nu}_{i-1}^{(j,R)}\right)-y_i\right)^2}{\sigma^2}\right)$

% As the 1st, 2nd and 4th factor do not contain $\hat{\nu}_i$ and max is equal to min(-log(.)) , define h as

$$h\left(\hat{\nu}_i^{(j)}\right) = \left(\hat{\nu}_i^{(j)} - \tilde{\nu}_i^{(j)}\right).\left(Cov_i^{(j)}\right)^{-1}.\left(\hat{\nu}_i^{(j)} - \tilde{\nu}_i^{(j)}\right) + \frac{\left(\left(\hat{\nu}_i^{(j,T)}+\hat{\nu}_i^{(j,R)}-\hat{\nu}_{i-1}^{(j,T)}+\hat{\nu}_{i-1}^{(j,R)}\right)-y_i\right)^2}{\sigma^2}$$

% and minimize by solving derivative equals 0.

$$\begin{pmatrix} \frac{\partial h(\hat{\nu}_i^{(j)})}{\partial \hat{\nu}_i^{(j,S)}} = 0 \\ \frac{\partial h(\hat{\nu}_i^{(j)})}{\partial \hat{\nu}_i^{(j,I)}} = 0 \\ \frac{\partial h(\hat{\nu}_i^{(j)})}{\partial \hat{\nu}_i^{(j,T)}} = 0 \end{pmatrix}$$ *% solve the optimization problem analytically for* $\hat{\nu}_i^{(j,S)}$, $\hat{\nu}_i^{(j,I)}$ *and* $\hat{\nu}_i^{(j,T)}$

% % % % calculate state estimate in case of no observation noise

% The state estimate is calculated by maximizing the probability in equation 7 .

% LNA $\Rightarrow$ *p normal*

% taking into account that $y_i = \hat{\nu}_i^{(j,T)} + \hat{\nu}_i^{(j,R)} - \hat{\nu}_{i-1}^{(j,T)} + \hat{\nu}_{i-1}^{(j,R)}$ *(noise free observation), reduces one dimension of the optimization problem*

$$h\left(\hat{\nu}_i^{(j)}\right) = \left(g(\hat{\nu}_i^{(j)}) - \tilde{\nu}_i^{(j)}\right).(Cov_i^{(j)})^{-1}.\left(g(\hat{\nu}_i^{(j)}) - \tilde{\nu}_i^{(j)}\right)$$ *% define function h*

$$g\left(\hat{\nu}_i^{(j)}\right) := \begin{pmatrix} \hat{\nu}_i^{(j,S)} \\ N - \hat{\nu}_{i-1}^{(j,T)} - \hat{\nu}_i^{(j,S)} - \hat{\nu}_{i-1}^{(j,R)} - y_i \\ \hat{\nu}_i^{(j,T)} \end{pmatrix}$$ *% function g taking into account the relation of* y_i

$$\begin{pmatrix} \frac{dh(\hat{\nu}_i^{(j)})}{d\hat{\nu}_i^{(j,S)}} = 0 \\ \frac{dh(\hat{\nu}_i^{(j)})}{d\hat{\nu}_i^{(j,T)}} = 0 \end{pmatrix}$$ *% solve the optimization problem analytically for* $\hat{\nu}_i^{(j,S)}$ *and* $\hat{\nu}_i^{(j,T)}$

% % % Calculate state specific bounds in case of observation noise with standard deviation σ^2

$$\hat{\nu}_i^{(j,S)} = \min\left\{\max\left\{0, \hat{\nu}_i^{(j,S)}\right\}, N\right\}$$ *% within the bounds of 0 and population size*

$$\hat{\nu}_i^{(j,S)} = \min\left\{\max\left\{0, \hat{\nu}_i^{(j,S)}\right\}, N\right\}$$

$$\hat{\nu}_i^{(j,S)} = \min\left\{\max\left\{0, \hat{\nu}_i^{(j,S)}\right\}, N\right\}$$

% % % Calculate state specific bounds in case of no observation noise

$$\hat{\nu}_i^{(j,S)} = \min\left(\hat{\nu}_i^{(j,S)}, N-1, N - \hat{\nu}_{i-1}^{(j,T)} - \hat{\nu}_{i-1}^{(j,R)} - y_i - \alpha, N - \textstyle\sum_{l=1}^{i} y_l - 1\right) \quad \text{\% we use } \alpha = 10^{-6}$$

% upper bounds: population size, population size - already diagnosed cases - current cases,

% population size - all cases; whichever is lowest

$$\hat{\nu}_i^{(j,T)} = \min\left(\max\left(0, \hat{\nu}_i^{(j,T)}\right), \textstyle\sum_{l=1}^{i} y_l\right)$$ *% no more people in T than already diagnosed cases*

$$\hat{\nu}_i^{(j,I)} = N - \hat{\nu}_{i-1}^{(j,T)} - \hat{\nu}_i^{(j,S)} - \hat{\nu}_{i-1}^{(j,R)} - y_i$$

% % % calculate likelihood

% need to integrate over state space $\rightarrow$ *sampling based*

$$\text{FOR } l = 1, \ldots, n_r : z_l^{(j)} \sim \mathcal{N}\left(\hat{\nu}_i^{(j)}, Cov_i^{(j)}\right) \text{ END FOR} \qquad \text{\%}\ n_r = 10000$$

$$newlys_{(i,l)}^{(j)} = z_l^{(j,T)} - \hat{\nu}_{i-1}^{(j,T)} + \left(N - z_l^{(j,S)} - z_l^{(j,I)} - z_l^{(j,T)}\right) - \hat{\nu}_{i-1}^{(j,R)}$$

$$dist = \mathcal{N}_{truncated(-10^{-10},N)}\left(\text{mean}\left(newlys_{(i,l=1,\ldots,n_r)}^{(j)}\right), \text{ Variance }\left(newlys_{(i,l=1,\ldots,n_r)}^{(j)}\right) + \sigma^2\right)$$

$$L(y_i|\theta^{(j)}) = PDF(dist, y_i)$$

END FOR

$$L^{(\text{MSS})}(Y_i|\theta^{(j)}) = L^{(\text{MSS})}(Y_{i-1}|\theta^{(j)}) \times L^{(\text{MSS})}(y_i|\theta^{(j)})$$

END FOR

4. **Output:** sample of parameters $\theta^{(j)}$ with corresponding likelihood values $L(Y_n|\theta^{(j)})$ and belief states.

S1.9 text

Detailed pseudo code for I.Poi. for SITR

1. **Input:**
   - Stochastic simulation model $H(t, x_0; \theta)$ simulating a trajectory until time t with an initial value x_0 and a parameter θ.
   - Data set $y_1, \dots, y_n$
   - Number of ensemble members N_{grid}.
   - Total populations size N.
   - Number of simulation being carried out for each parameter value $N_{prio,sim}$.

2. **Initialization:**
   FOR j=1,...,N_{grid}
   $\theta_0^{(j)} = \left(R_0^{(j)}, mdi_0^{(j)}\right) \sim U(R_0^{(\text{range})} \times mdi^{(\text{range})})$ % mdi - mean duration of infectiousness
   % in our case $R_0^{(\text{range})} = [1, 3]$, $mdi^{(\text{range})} = [1, 20]$, $\gamma^{(\text{range})} = [0, 5]$.
   $S_0^{(j)} = N - 1$
   $I_0^{(j)} = 1$
   $T_0^{(j)} = R_0^{(j)} = 0$
   $\nu_0^{(j)} = \left(S_0^{(j)}, I_0^{(j)}, T_0^{(j)}, R_0^{(j)}\right)$
   $L(Y_0|\theta^{(j)}) = 1$
   END FOR

3. **Calibration:**
   FOR $i = 1, \dots, n$
   FOR j=1,...,N_{grid}
   For $k = 1, \dots, N_{prio,sim}$
   $z_{i-1}^{(j,k)} = H^{(k)}(t_{i-1}, \nu_0; \theta^{(j)})$ % previous state
   $z_i^{(j,k)} = H^{(k)}(t_i, \nu_0; \theta^{(j)})$ % current state
   $newly_i^{(j,k)} = z_i^{(j,k,T)} + z_i^{(j,k,R)} - z_{i-1}^{(j,k,T)} - z_{i-1}^{(j,k,R)}$
   END FOR k
   $me = \text{mean}\left(newly_i^{(j,1)}, \dots, newly_i^{(j,N_{prio,sim})}\right)$ % calculate mean for Poisson distribution
   IF $me > 0$ % Poisson distribution needs parameter greater 0
   $L(y_i|\theta^{(j)}) = PDF(PoissonDistribution(mean), y_i)$ % if so, evaluate
   ELSE: IF $y_i = 0$ then 1 ELSE $\tilde{\alpha}$ END IF % if not, check whether $y_i = 0$, we use $\tilde{\alpha} = 10^{-100}$.
   END IF
   END FOR
   $L^{(\text{I.Poi})}(Y_i|\theta^{(j)}) = L^{(\text{I.Poi})}(Y_{i-1}|\theta^{(j)}) \times L^{(\text{I.Poi})}(y_i|\theta^{(j)})$ % update likelihood
   END FOR

4. **Output:** sample of parameters θ with corresponding likelihood values $L(Y_i|\theta)$.

S1.10 text

Detailed pseudo code for Particle Filter for SITR

1. **Input:**
   - ODE solution $x(t, x_0; \theta)$ for an ODE x' integrated for time t with initial value x_0 and parameter θ such as in equation (5).
   - Data set $y_1, \ldots, y_n$
   - Number of ensemble members N_{grid}.
   - Total populations size N.

2. **Initialization:**

   FOR j=1,...,N_{grid}

   $\theta_0^{(j)} = \left(R_{0_0}^{(j)}, mdi_0^{(j)}\right) \sim U(R_0^{(\text{range})} \times mdi^{(\text{range})})$ % mdi - mean duration of infectiousness

   % in our case $R_0^{(\text{range})} = [1, 3]$, $mdi^{(\text{range})} = [1, 20]$, $\gamma^{(\text{range})} = [0, 5]$.

   $S_0^{(j)} \sim U([\bar{\alpha}N, N])$ % we use $\bar{\alpha} = 0.9$

   $I_0^{(j)} \sim U([0, N - S_0^{(j)}])$

   $T_0^{(j)} \sim U([0, N - S_0^{(j)} - I_0^{(j)}])$

   $R_0^{(j)} \sim U([0, N - S_0^{(j)} - I_0^{(j)} - T_0^{(j)}])$

   $\hat{\nu}_0^{(j)} = \left(S_0^{(j)}, I_0^{(j)}, T_0^{(j)}, R_{0_0}^{(j)}\right)$

   $w_0^{(j)} = 1/N_{grid}$

   END FOR

3. **Loop:**

   FOR i=1,...,n

   $W_{i-1} = \sum_{j=1}^{N_{grid}} w_{i-1}^{(j)}$

   FOR j=1,...,N_{grid}: $\tilde{w}_{i-1}^{(j)} = w_{i-1}^{(j)}/W_i$ END FOR % normalize weights

   $n_{eff} = 1/\sum_{j=1}^{N_{grid}} (\tilde{w}_{i-1}^{(j)})^2$

   IF $n_{eff} < N_{grid}/2$ % check whether to re-sample

   $n_1 = 7$

   $C_1 = \Pi^{n_1/2}/\text{Gamma}(n_1/2 + 1)$ % with the gamma function Gamma

   $A_1 = (8/C_1(n_1 + 4) * (2\sqrt{\Pi})^{n_1})^{-1/(n_1+4)}$

   $h_{opt} = 2 * A_1 * N_{grid}^{-1/(n_1+4)}$

   $x_K = (-1, -0.999, \ldots, 0.999, 1)$

   $d_K = ((n_1 + 2)/2/C_1) * (1 - x_K^2)$ % kernel

   $c_K = d_K = d_K / \sum_{j=1}^{\text{length}(d_K)} d_{Kj}$

   $FOR\ \ j = 1, \ldots, \text{length}(d_K):\ \ c_{Kj} = c_{Kj-1} + d_{Kj}$ END FOR % cumulative

   f: function interpolating (x_K, c_K)

   $\psi_{i-1}^{(j)}$ drawn from $\left\{\left(\theta_{i-1}^{(1)}, \hat{\nu}_{i-1}^{(1)}\right), \ldots, \left(\theta_{i-1}^{(N_{grid})}, \hat{\nu}_{i-1}^{(N_{grid})}\right)\right\}$ with weights $\left\{\tilde{w}_{i-1}^{(1)}, \ldots, \tilde{w}_{i-1}^{(N_{grid})}\right\}$

   $\tilde{w}_{i-1}^{(j)} = 1/N_{grid}, j = 1, \ldots, N_{grid}$

$\sigma_{i-1} = StdDev\left(\psi^{(1)}_{i-1}, \dots, \psi^{(N_{grid})}_{i-1}\right)$ % vector of standard deviations

IF length $\left(\cup_{j=1}^{N_{grid}} \left\{\psi^{(j)}_{i-1}\right\}\right) < \max(\alpha_1, 0.01 N_{grid})$ % we use $\alpha_1 = 20$.

$\sigma_{i-1} = StdDev\left(\psi^{(1)}_{\max(i-2-1,0)}, \dots, \psi^{(N_{grid})}_{\max(i-2-1,0)}\right)$

$\sigma^{(I)}_{i-1} = \max\left(\sigma^{(I)}_{i-1}, y_i/5\right)$

END IF

FOR $k = 1, \dots, n_1$:

$z_k = f(\Xi)$ with a $\Xi \sim UniformDistribution\left([0,1]^{N_{grid}}\right)$

END FOR

$\psi^{(j)}_{i-1} = \psi^{(j)}_{i-1} + h_{opt}\ z\ SD$ % with SD a diagonal matrix with entries σ_{i-1} regularization noise

$FOR\ j = 1, \dots, N_{grid}$ % check boundaries

$\hat{\nu}^{(j,m)}_{i-1} = \max\left(0, \min\left(N, \psi^{(j,m)}_{i-1}\right)\right), m \in \{S, I, T, R\}$

$\theta^{(j,R_0)}_{i-1} = \max\left(1, \min\left(R_0^{(\text{range,up})}, \psi^{(j,R_0)}_{i-1}\right)\right)$

$\theta^{(j,mdi)}_{i-1} = \max\left(1, \min\left(mdi^{(\text{range,up})}, \psi^{(j,mdi)}_{i-1}\right)\right)$,

END FOR

ELSE

nothing % do not change θ or $\hat{\nu}$.

END IF

% % % *Forward Propagation*

FOR $j = 1, \dots, N_{grid}$

$\hat{\nu}^{(j)}_i = x(t_i - t_{i-1}, \hat{\nu}^{(j)}_{i-1}; \theta^{(j)}_{i-1})$ % forward propagation

$\theta^{(j)}_i = \theta^{(j)}_{i-1}$

END FOR

% % % *Assign weights*

FOR $j = 1, \dots, N_{grid}$

$y^{(j)}_{i,prior} = \hat{\nu}^{(j,T)}_i + \hat{\nu}^{(j,R)}_i - \hat{\nu}^{(j,T)}_{i-1} - \hat{\nu}^{(j,R)}_{i-1}$ % prior observations

$\sigma^2_{i,obs} = 10000 + \frac{1}{50}\left(\frac{1}{3}\sum_{j=i-3}^{i-1} y_j\right)^2.$ % observation variance

$w^{(j)}_i = \tilde{w}^{(j)}_{i-1} \times PDF\left(\mathcal{N}\left(y^{(j)}_{i,prior}, \sigma^2_{i,obs}\right), y_i\right)$

END FOR

END FOR

4. **Output:** posterior sample of parameters θ_n and states $\hat{\nu}_n$.

S1.11 text

Detailed pseudo code for the Ensemble Kalman filter for SITR

1. **Input:**
   - ODE solution $x(t, x_0; \theta)$ for an ODE x' integrated until time t with initial value x_0 and parameter θ such as in equation (5).
   - Data set $y_1, \ldots, y_n$
   - Number of ensemble members N_{grid}.
   - Total populations size N.

2. **Initialization:**

FOR j=1,...,N_{grid}

$\theta_0^{(j)} = \left(R_{00}^{(j)}, mdi_0^{(j)}\right) \sim U(R_0^{(\text{range})} \times mdi^{(\text{range})})$ % mdi - mean duration of infectiousness

% in our case $R_0^{(\text{range})} = [1,3]$, $mdi^{(\text{range})} = [1,20]$, $\gamma^{(\text{range})} = [0,5]$.

$S_0^{(j)} \sim U\left([\bar{\alpha}N, N]\right)$ % we use $\bar{\alpha} = 0.9$.

$I_0^{(j)} \sim U\left([0, N - S_0^{(j)}]\right)$

$T_0^{(j)} \sim U\left([0, N - S_0^{(j)} - I_0^{(j)}]\right)$

$R_0^{(j)} \sim U\left([0, N - S_0^{(j)} - I_0^{(j)} - T_0^{(j)}]\right)$

$\hat{\nu}_0^{(j)} = \left(S_0^{(j)}, I_0^{(j)}, T_0^{(j)}, R_{00}^{(j)}\right)$

$\psi_0^{(j)} = \left(\hat{\nu}_0^{(j)}, \theta_0^{(j)}, y_0 = 0\right)$

END FOR

3. **Loop:**

FOR i=1,...,n

% % Forward propagation

FOR j=1,...,N_{grid}

$\nu_{i,prior}^{(j)} = x(t_i - t_{i-1}, \hat{\nu}_{i-1}^{(j)}; \theta_{i-1}^{(j)})$ % propagation

END FOR

FOR j=1,...,N_{grid}

$\nu_{i,prior}^{(j)} = \alpha' \left(\nu_{i,prior}^{(j)} - \text{mean}\left(\nu_{i,prior}^{(1)}, \ldots, \nu_{i,prior}^{(N_{grid})}\right)\right) + \nu_{i,prior}^{(j)}$

% Inflation with an inflation paramter $\alpha' = 1.07$

$\psi_i^{(y,j)} = \nu_{i,prior}^{(j,T)} + \nu_{i,prior}^{(j,R)} - \hat{\nu}_{i-1}^{(j,T)} - \hat{\nu}_{i-1}^{(j,R)}$ % prior observations

$\sigma_{i,obs}^2 = 10000 + \frac{1}{50}\left(\frac{1}{3}\sum_{j=i-3}^{i-1} y_j\right)^2.$ % observation variance

$\sigma_{i,prior}^2 = \text{variance}\left(\psi_i^{(1,y)}, \ldots, \psi_i^{(N_{grid},y)}\right)$ % prior variance

$\sigma_i^{(m)} = \text{co-variance}\left(\psi_i^y, \nu_{i,prior}^{(m)}\right), m \in \{R_0, mdi, S, I, T, R\}$ % co-variance observation prior

$\tilde{y}_i^{(j)} = \max\left(0, y_i + \xi_i^{(j)}\right)$ with $\xi_i^{(j)} \sim \mathcal{N}(0, \sigma_{i,obs}^2)$ % noisy observation

END FOR

% % Update

FOR j=1,...,N_{grid}

$\delta_i^{(j)} = \frac{\sigma_{i,obs}^2}{\sigma_{i,obs}^2 + \sigma_{i,prior}^2}\psi_i^{(y,j)} + \frac{\sigma_{i,prior}^2}{\sigma_{i,obs}^2 + \sigma_{i,prior}^2}\tilde{y}_i^{(j)}.$

$\theta_i^{(j,m)} = \frac{\sigma_i^{(m)}}{\sigma_{i,prior}^2}\delta_i^{(j)} + \theta_{i-1}^{(j,m)}, m \in \{R_0, mdi\}$
$\hat{\nu}_i^{(j,m)} = \frac{\sigma_i^{(m)}}{\sigma_{i,prior}^2}\delta_i^{(j)} + \hat{\nu}_{i-1}^{(j,m)}, m \in \{S, I, T, R\}$
END FOR

% % Check boundaries
FOR j=1,...,N_{grid}
$\hat{\nu}_i^{(j,S)} =$ IF $\hat{\nu}_i^{(j,S)} > N : N-1$ ELSE $\hat{\nu}_i^{(j,S)}$ END IF
$\hat{\nu}_i^{(j,m)} =$ IF $\hat{\nu}_i^{(j,m)} > N : \text{median}\left(\hat{\nu}_i^{(j=1,\ldots,N_{grid},m)}\right)$ ELSE $\hat{\nu}_i^{(j,m)}, m \in \{I, T, R\}$ END IF
$\theta_i^{(j,R_0)} =$ IF $\theta_i^{(j,R_0)} > R_0^{(\text{range,up})} : \min\left(R_0^{(\text{range,up})}, \text{median}\left(\theta_i^{(j=1,\ldots,N_{grid},R_0)}\right)\right)$ ELSE $\theta_i^{(j,R_0)}$ END IF
$\theta_i^{(j,mdi)} =$ IF$\theta_i^{(j,mdi)} > mdi^{(\text{range,up})} : \min\left(mdi^{(\text{range,up})}, \text{median}\left(\theta_i^{(j=1,\ldots,N_{grid},mdi)}\right)\right)$ ELSE $\theta_i^{(j,mdi)}$
END IF
$\hat{\nu}_i^{(j,m)} =$ IF $\hat{\nu}_i^{(j,m)} < 0 : \max\left(1, \text{mean}\left(\hat{\nu}_i^{(j=1,\ldots,N_{grid},m)}\right)\right)$ ELSE $\hat{\nu}_i^{(j,m)}, m \in \{S, I, T, R\}$ END IF
$\theta_i^{(j,m)} =$ IF $\theta_i^{(j,m)} < 0 : \max\left(1, \text{mean}\left(\theta_i^{(j=1,\ldots,N_{grid},m)}\right)\right)$ ELSE $\theta_i^{(j,m)}, m \in \{R_0, mdi\}$ END IF
END FOR
END FOR

4. **Output:** a posteriori sample of parameters θ_n and states $\hat{\nu}_n$.

References

1. Lipsitch M, Finelli L, Heffernan RT, Leung GM, Redd; for the 2009 H1N1 Surveillance Group SC. Improving the evidence base for decision making during a pandemic: the example of 2009 influenza A/H1N1. Biosecurity and bioterrorism: biodefense strategy, practice, and science. 2011;9(2):89–115.

2. Mills CE, Robins JM, Lipsitch M. Transmissibility of 1918 pandemic influenza. Nature. 2004;432(7019):904–906.

3. Cauchemez S, Donnelly CA, Reed C, Ghani AC, Fraser C, Kent CK, et al. Household transmission of 2009 pandemic influenza A (H1N1) virus in the United States. New England Journal of Medicine. 2009;361(27):2619–2627.

4. Cowling BJ, Lau MS, Ho LM, Chuang SK, Tsang T, Liu SH, et al. The effective reproduction number of pandemic influenza: prospective estimation. Epidemiology. 2010;21(6):842.

5. Cori A, Ferguson NM, Fraser C, Cauchemez S. A new framework and software to estimate time-varying reproduction numbers during epidemics. American journal of epidemiology. 2013;178(9):1505–1512.

6. Cauchemez S, Epperson S, Biggerstaff M, Swerdlow D, Finelli L, Ferguson NM. Using routine surveillance data to estimate the epidemic potential of emerging zoonoses: application to the emergence of US swine origin influenza A H3N2v virus. PLoS Med. 2013;10(3):e1001399.

7. Cauchemez S, Carrat F, Viboud C, Valleron A, Boelle P. A Bayesian MCMC approach to study transmission of influenza: application to household longitudinal data. Statistics in medicine. 2004;23(22):3469–3487.

8. Höhle M, Jørgensen E, O'Neill PD. Inference in disease transmission experiments by using stochastic epidemic models. Journal of the Royal Statistical Society: Series C (Applied Statistics). 2005;54(2):349–366.

9. White LF, Pagano M. A likelihood-based method for real-time estimation of the serial interval and reproductive number of an epidemic. Stat Med. 2008 Jul;27(16):2999–3016.

10. O'Neill PD. A tutorial introduction to Bayesian inference for stochastic epidemic models using Markov chain Monte Carlo methods. Mathematical biosciences. 2002;180(1):103–114.

11. Obadia T, Haneef R, Boelle PY. The R0 package: a toolbox to estimate reproduction numbers for epidemic outbreaks. BMC Med Inform Decis Mak. 2012;12:147.

12. Cauchemez S, Boelle PY, Donnelly CA, Ferguson NM, Thomas G, Leung GM, et al. Real-time estimates in early detection of SARS. Emerging Infect Dis. 2006 Jan;12(1):110–113.

13. Cauchemez S, Boëlle PY, Thomas G, Valleron AJ. Estimating in real time the efficacy of measures to control emerging communicable diseases. American Journal of Epidemiology. 2006;164(6):591–597.

14. Wallinga J, Teunis P. Different epidemic curves for severe acute respiratory syndrome reveal similar impacts of control measures. American Journal of Epidemiology. 2004;160(6):509–516.

15. Davoudi B, Miller JC, Meza R, Meyers LA, Earn DJD, Pourbohloul B. Early real-time estimation of the basic reproduction number of emerging infectious diseases. Physical Review X. 2012;2.

16. Cauchemez S, Ferguson NM. Methods to infer transmission risk factors in complex outbreak data. J R Soc Interface. 2012 Mar;9(68):456–469.

17. Saramaki J, Kaski K. Modelling development of epidemics with dynamic small-world networks. J Theor Biol. 2005 Jun;234(3):413–421.

18. Anderson RM, May RM. Infectious Diseases of Humans: Dynamics and Control. Oxford University Press; 1992.

19. Daley DJ, Gani JM. Epidemic Modelling: An Introduction. Cambridge; New York: Cambridge University Press; 1999.

20. Alkema L, Raftery AE, Clark SJ. Probabilistic projections of HIV prevalence using Bayesian melding. The Annals of Applied Statistics. 2007;p. 229–248.

21. Elderd BD, Dukic VM, Dwyer G. Uncertainty in predictions of disease spread and public health responses to bioterrorism and emerging diseases. Proceedings of the National Academy of Sciences. 2006;103(42):15693–15697.

22. Birrell PJ, Ketsetzis G, Gay NJ, Cooper BS, Presanis AM, Harris RJ, et al. Bayesian modeling to unmask and predict influenza A/H1N1pdm dynamics in London. Proceedings of the National Academy of Sciences. 2011;108(45):18238–18243.

23. Riley S, Fraser C, Donnelly CA, Ghani AC, Abu-Raddad LJ, Hedley AJ, et al. Transmission dynamics of the etiological agent of SARS in Hong Kong: impact of public health interventions. Science. 2003;300(5627):1961–1966.

24. Choi B, Rempala GA. Inference for discretely observed stochastic kinetic networks with applications to epidemic modeling. Biostatistics. 2012 Jan;13(1):153–165.

25. Ionides EL, Breto C, King AA. Inference for nonlinear dynamical systems. Proc Natl Acad Sci USA. 2006 Dec;103(49):18438–18443.

26. Shaman J, Karspeck A, Yang W, Tamerius J, Lipsitch M. Real-time influenza forecasts during the 2012-2013 season. Nat Commun. 2013;4:2837.

27. Yang W, Karspeck A, Shaman J. Comparison of Filtering Methods for the Modeling and Retrospective Forecasting of Influenza Epidemics. PLOS Computational Biology. 2014;10:e1003583.

28. Yang W, Cowling BJ, Lau EH, Shaman J. Forecasting Influenza Epidemics in Hong Kong. PLoS Comput Biol. 2015 Jul;11(7):e1004383.

29. Shaman J, Karspeck A. Forecasting seasonal outbreaks of influenza. Proc Natl Acad Sci USA. 2012 Dec;109(50):20425–20430.

30. Ong JB, Chen MI, Cook AR, Lee HC, Lee VJ, Lin RT, et al. Real-time epidemic monitoring and forecasting of H1N1-2009 using influenza-like illness from general practice and family doctor clinics in Singapore. PLoS ONE. 2010;5(4):e10036.

31. Dukic V, Lopes HF, Polson NG. Tracking Epidemics With Google Flu Trends Data and a State-Space SEIR Model. Journal of the American Statistical Association. 2012;107.

32. Chretien JP, George D, Shaman J, Chitale RA, McKenzie FE. Influenza forecasting in human populations: a scoping review. PLoS ONE. 2014;9(4):e94130.

33. Bettencourt LM, Ribeiro RM. Real time Bayesian estimation of the epidemic potential of emerging infectious diseases. PLoS One. 2008;3(5):e2185.

34. Abbey H. An examination of the Reed-Frost theory of epidemics. Human biology. 1952;24(3):201–233.

35. Zimmer C, Sahle S. Deterministic inference for stochastic systems using multiple shooting and a linear noise approximation for the transition probabilities. IET Systems Biology. 2015;9:181 – 192.

36. Zimmer C. Reconstructing the hidden states in time course data of stochastic models. Mathematical BioSciences. 2015;269:117 – 129.

37. Thomas P, Matuschek H, Grima R. Intrinsic noise analyzer: a software package for the exploration of stochastic biochemical kinetics using the system size expansion. PloS one. 2012;7(6):e38518.

38. Van Kampen NG. Stochastic processes in physics and chemistry. vol. 1. Elsevier; 1992.

39. Yaesoubi R, Cohen T. Generalized Markov models of infectious disease spread: A novel framework for developing dynamic health policies. European Journal of Operational Research. 2011;215:679–687.

40. Gillespie DT. A General Method for Numerically Simulating the Stochastic Time Evolution of coupled Chemical Reactions. Journal of Computational Physics. 1976;22 (4):403–434.

41. Influenza prediction challenge. Center for disease control and prevention. 2016;http://www.cdc.gov/flu/news/flu-forecast-website- launched.htm.

42. Hoops S, Sahle S, Gauges R, Lee C, Pahle J, Simus N, et al. COPASI - a COmplex PAthway SImulator. Bioinformatics. 2006;22 (24):3067–3074.

43. Mathematica, Version 10.4. Wolfram Research, Inc. 2015;Champaign, IL.

44. Pahle J, Challenger JD, Mendes P, McKane AJ. Biochemical fluctuations, optimisation and the linear noise approximation. BMC Systems Biology. 2012;6.

45. Challenger JD, McKane AJ, Pahle J. Multi-compartment linear noise approximation. Journal of Statistical Mechanics: Theory and Experiment. 2012;P11010.

46. Straube R, von Kamp A. LiNA – A Graphical Matlab Tool for Analyzing Intrinsic Noise in Biochemical Reaction Networks. online tutorial. 2013;http://www2.mpi-magdeburg.mpg.de/projects/LiNA/Tutorial_LiNA_v1.pdf.

47. van Kampen NG. Stochastic processes in physics and chemistry. Elsevier; 2007.

48. Grima R. An effective rate equation approach to reaction kinetics in small volumes: Theory and application to biochemical reactions in nonequilibrium steady-state conditions. The Journal of Chemical Physics. 2010;133:035101.

49. Thomas P, Matuschek H, Grima R. Intrinsic Noise Analyzer: A Software Package for the Exploration of Stochastic Biochemical Kinetics Using the System Size Expansion. Plos ONE. 2012;7:e38518.

50. Komorowski M, Finkenstädt B, Harper CV, Rand DA. Bayesian inference of biochemical kinetic parameters using the linear noise approximation. BMC Bioinformatics. 2009;10:343.
