# Supplementary figures and images for: A Likelihood Approach for Real-Time Calibration of Stochastic Compartmental Epidemic Models
Source: PLoS Comput Biol. 2017 Jan 17;13(1):e1005257. doi: 10.1371/journal.pcbi.1005257 (PMC5240920; doi:10.1371/journal.pcbi.1005257)

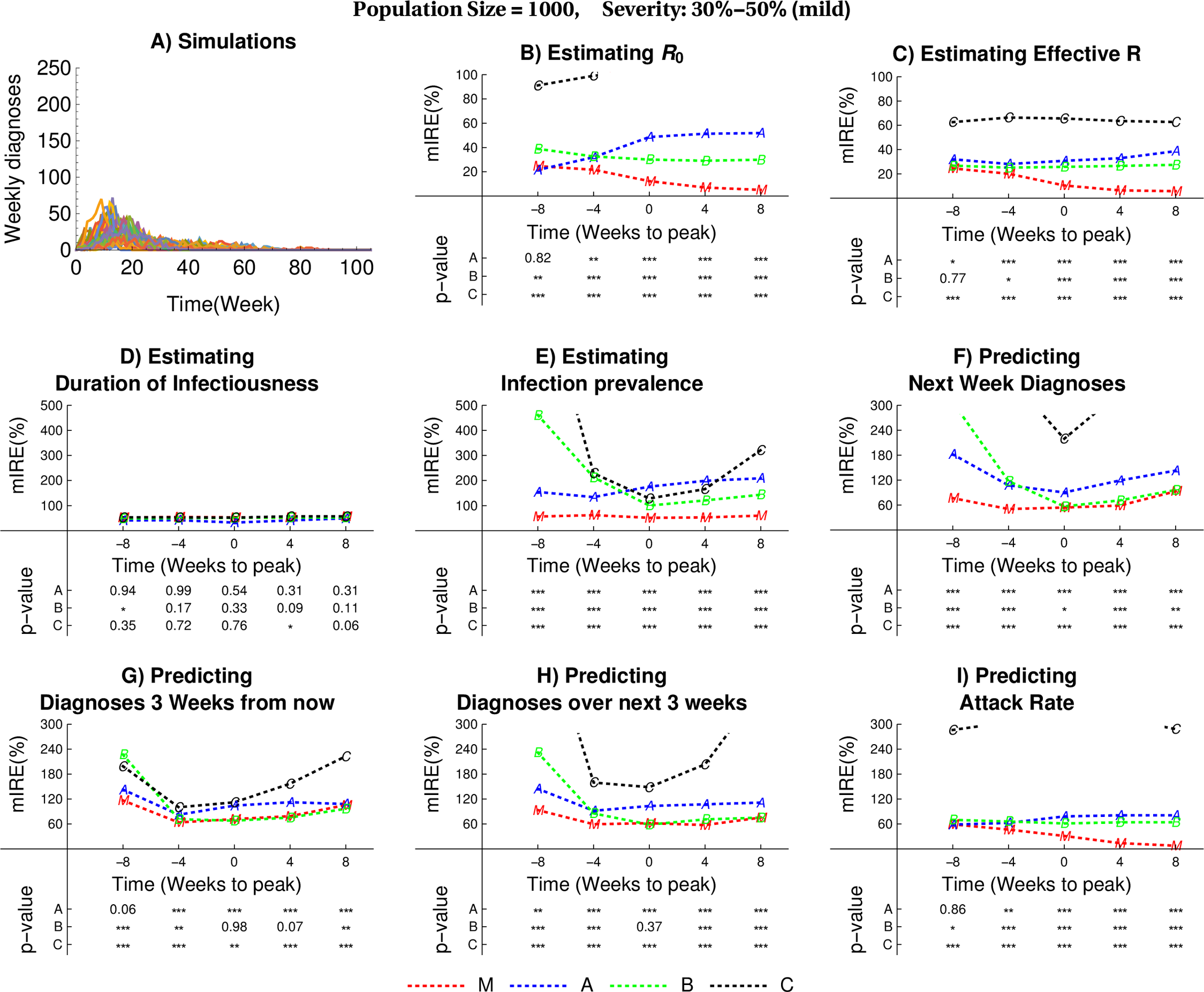

Supplement: S1 Fig — Same setting as in Fig 4. (TIF) [file pcbi.1005257.s002.tif]

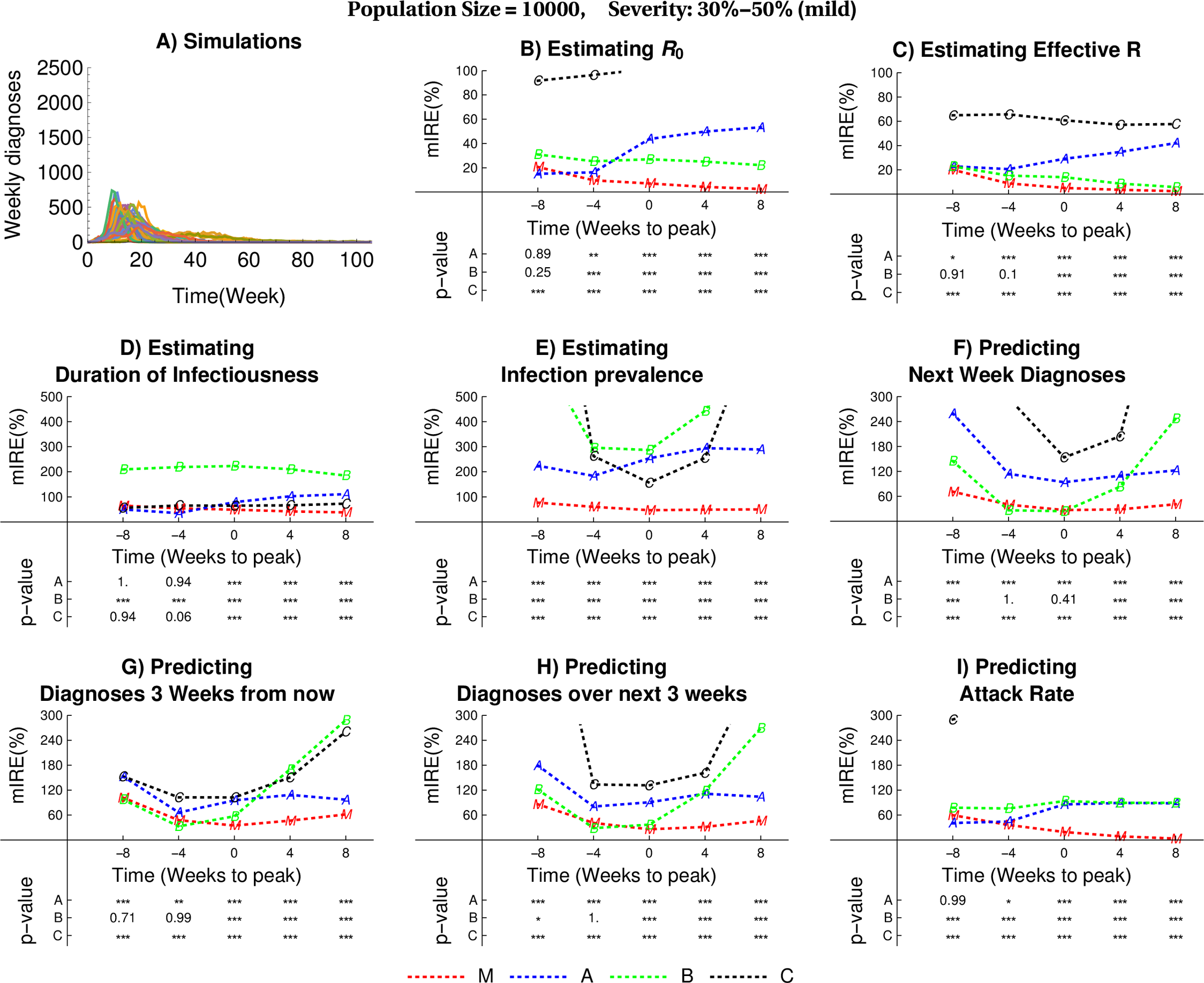

Supplement: S2 Fig — Same setting as in Fig 4. (TIF) [file pcbi.1005257.s003.tif]

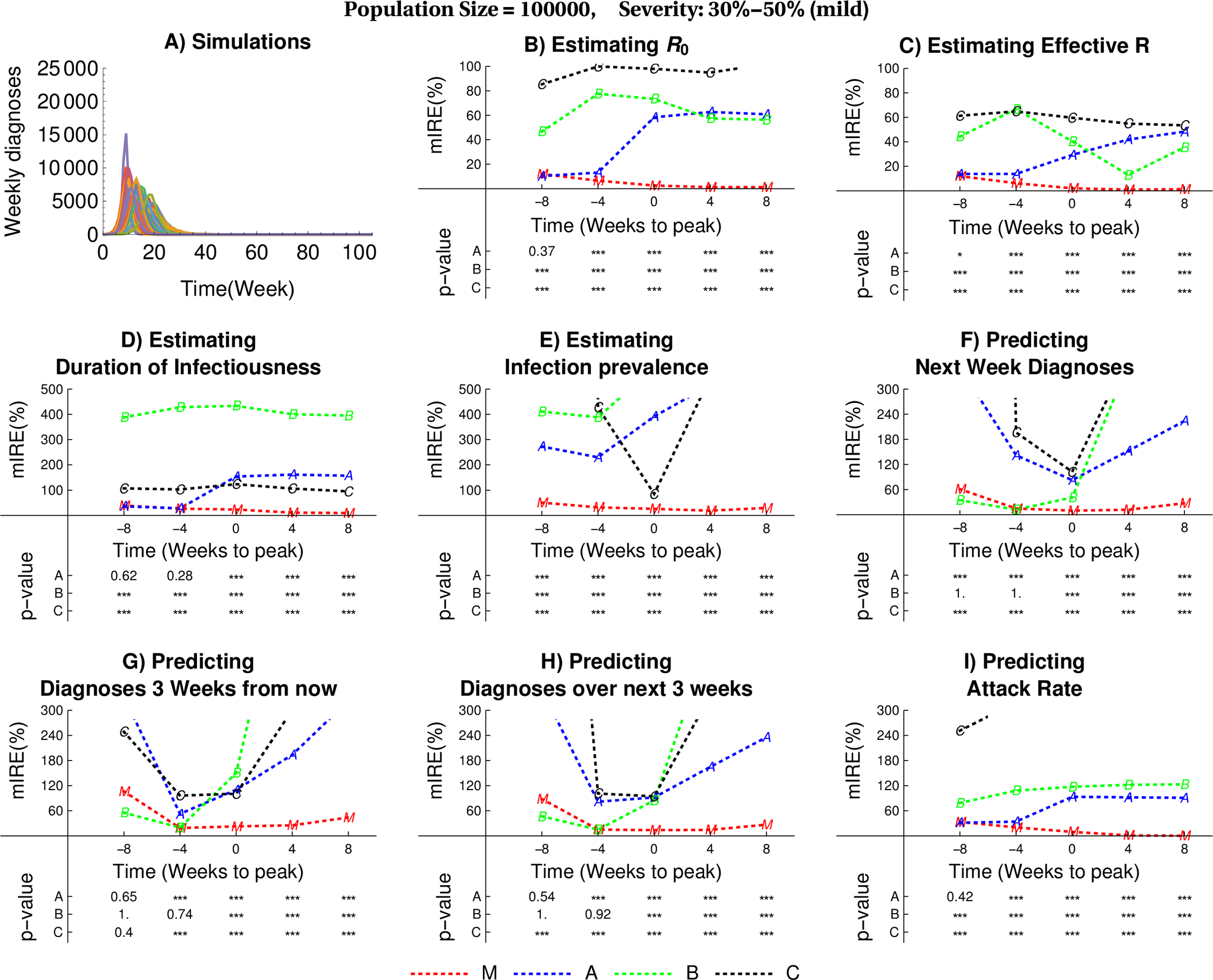

Supplement: S3 Fig — Same setting as in Fig 4. (TIF) [file pcbi.1005257.s004.tif]

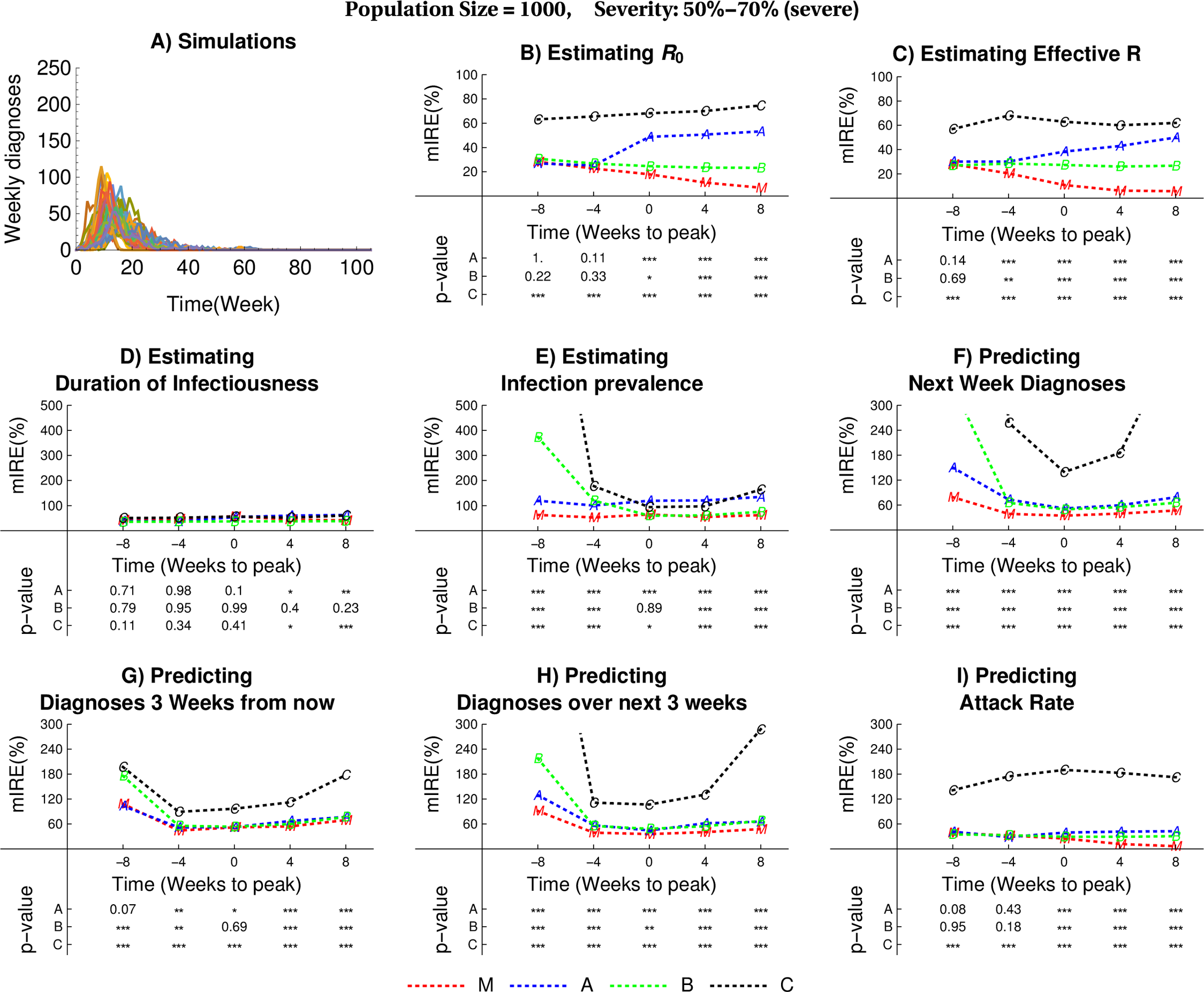

Supplement: S4 Fig — Same setting as in Fig 4. (TIF) [file pcbi.1005257.s005.tif]

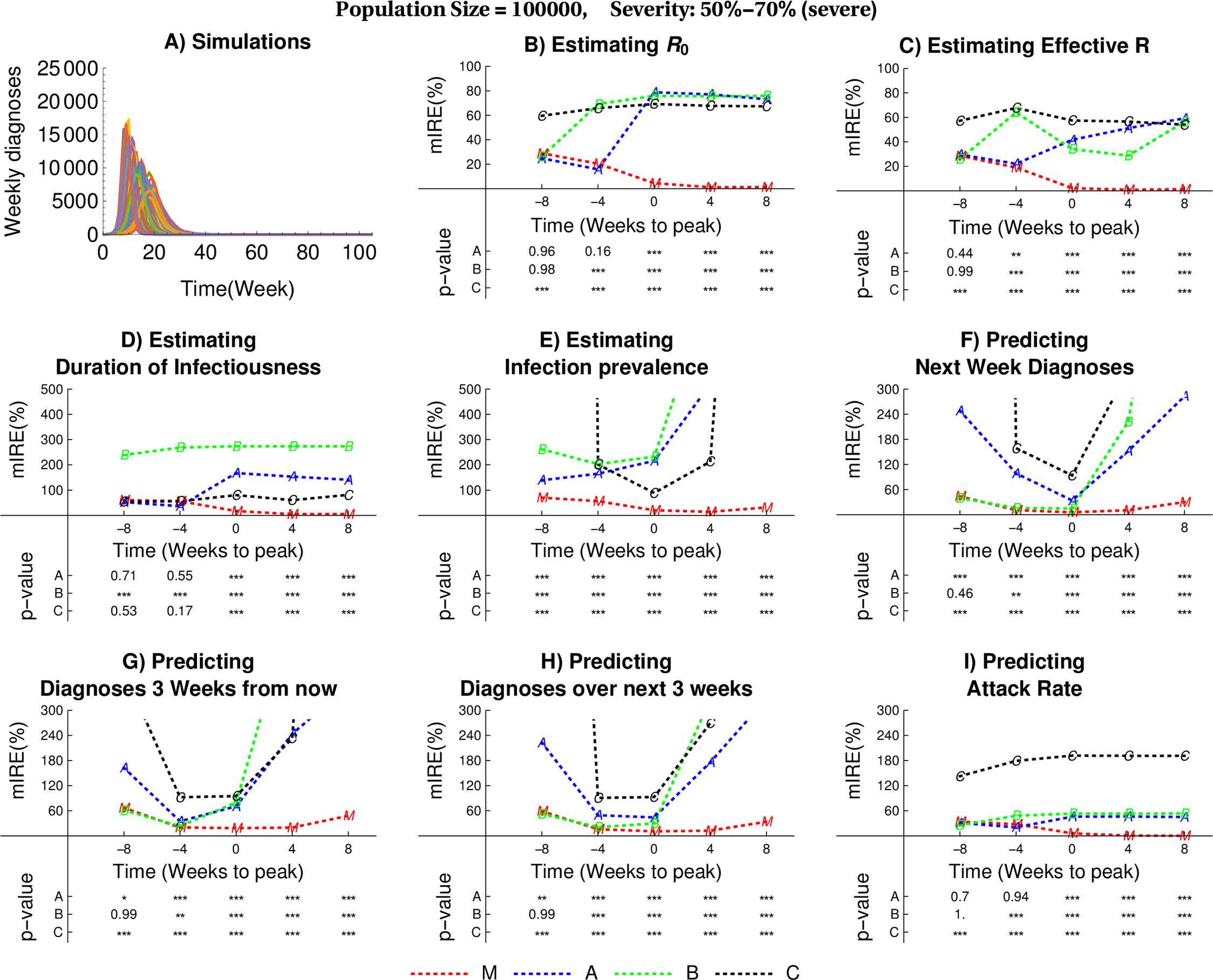

Supplement: S5 Fig — Same setting as in Fig 4. (TIF) [file pcbi.1005257.s006.tif]

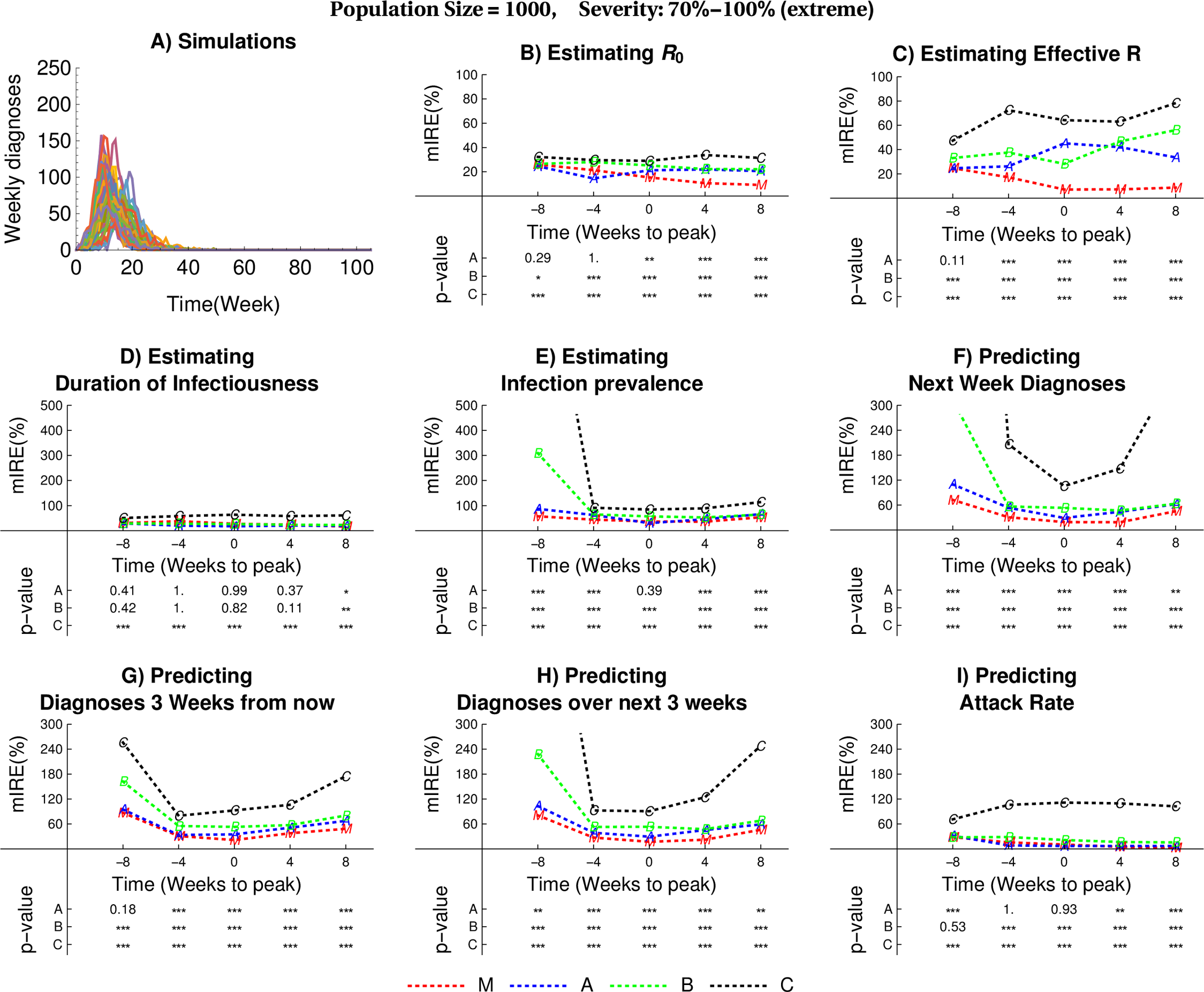

Supplement: S6 Fig — Same setting as in Fig 4. (TIF) [file pcbi.1005257.s007.tif]

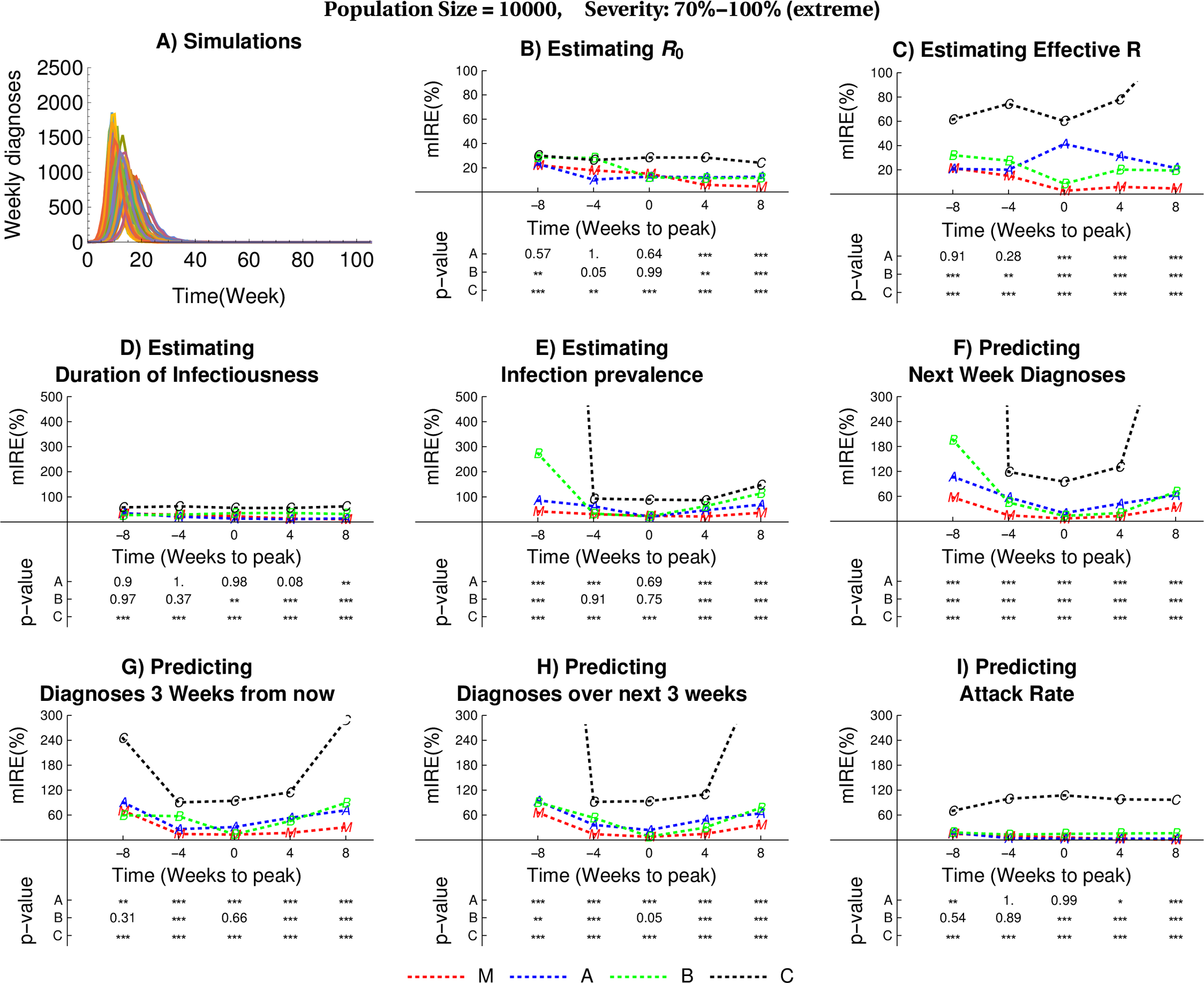

Supplement: S7 Fig — Same setting as in Fig 4. (TIF) [file pcbi.1005257.s008.tif]

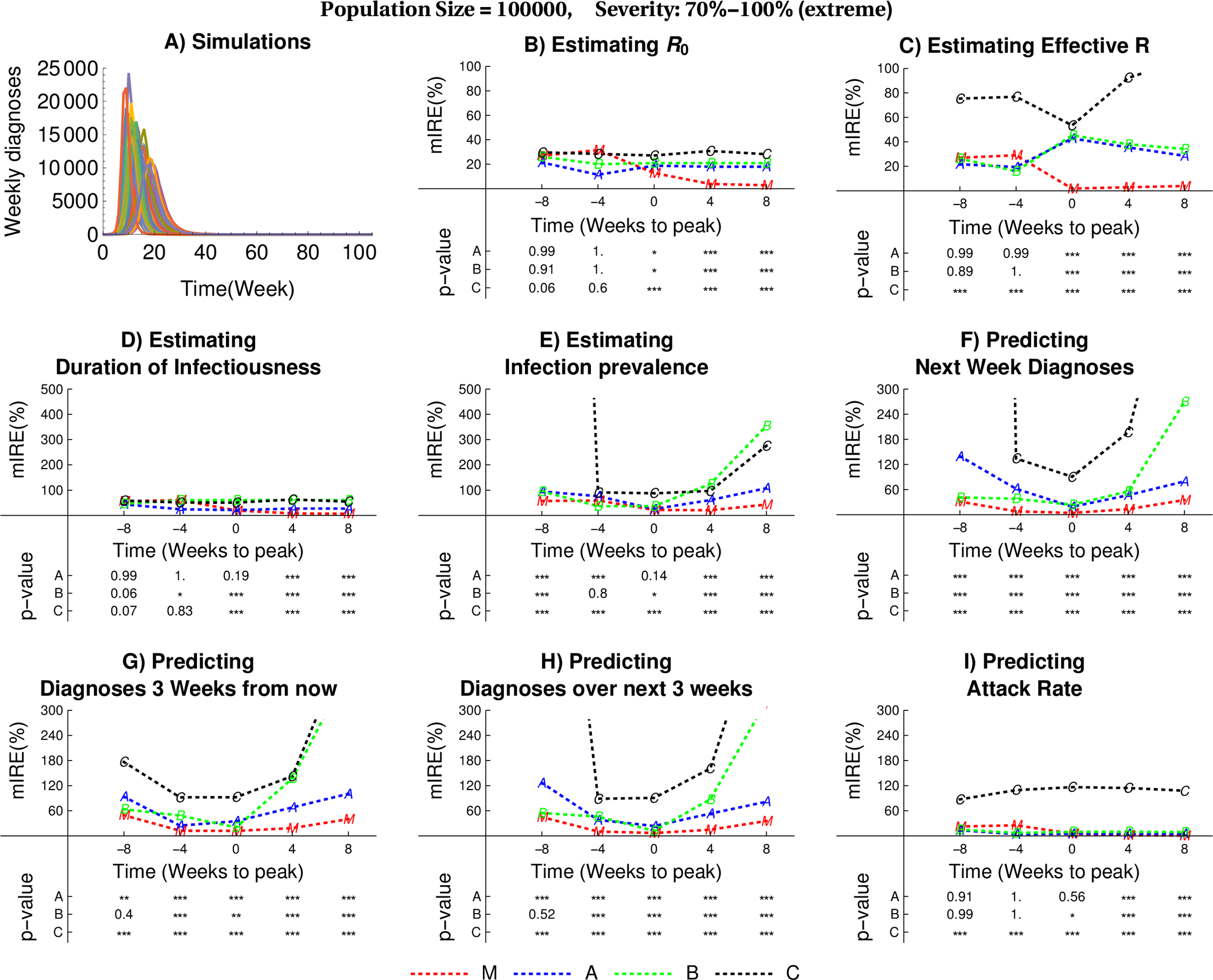

Supplement: S8 Fig — Same setting as in Fig 4. (TIF) [file pcbi.1005257.s009.tif]

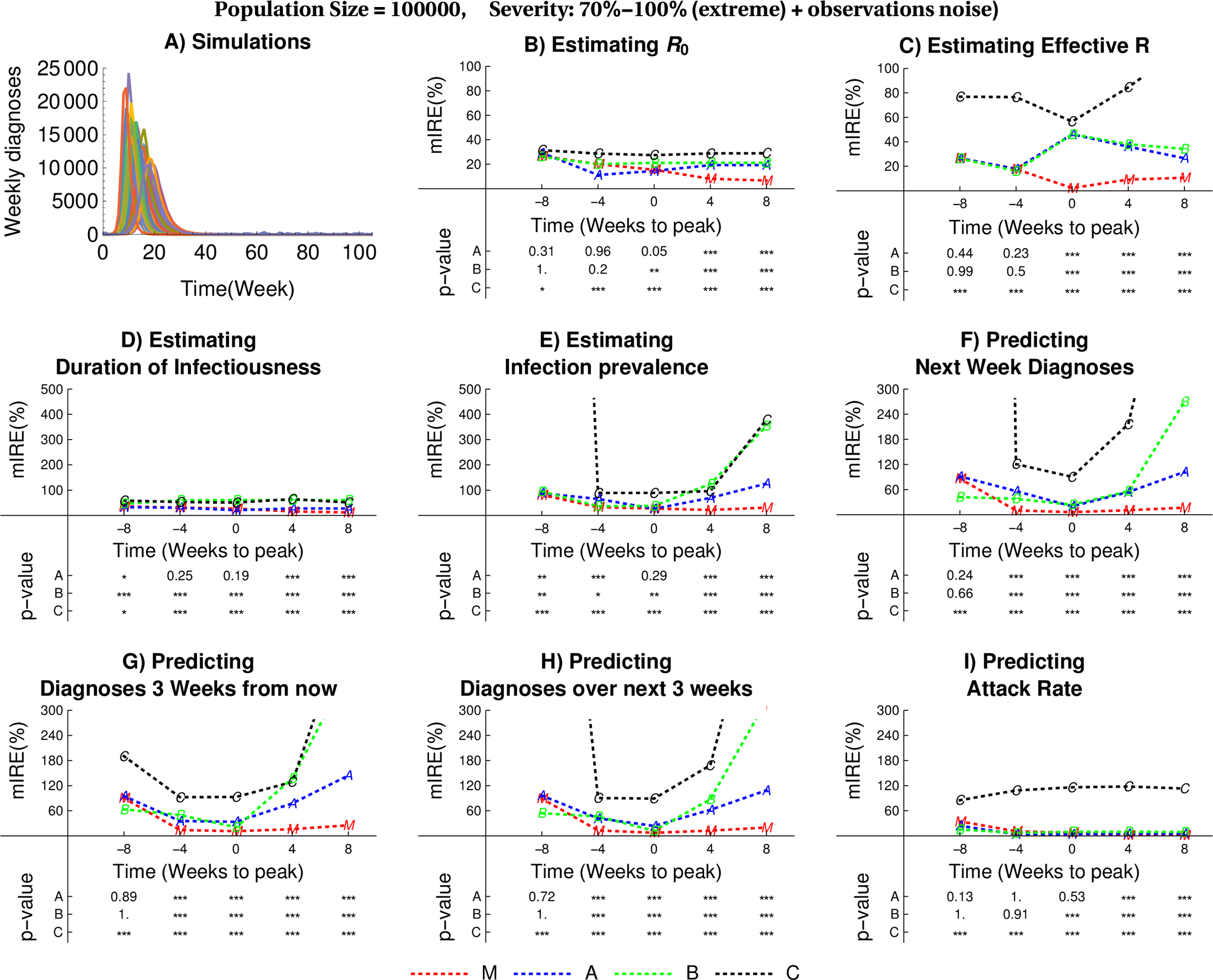

Supplement: S9 Fig — Same setting as in Fig 4. (TIF) [file pcbi.1005257.s010.tif]

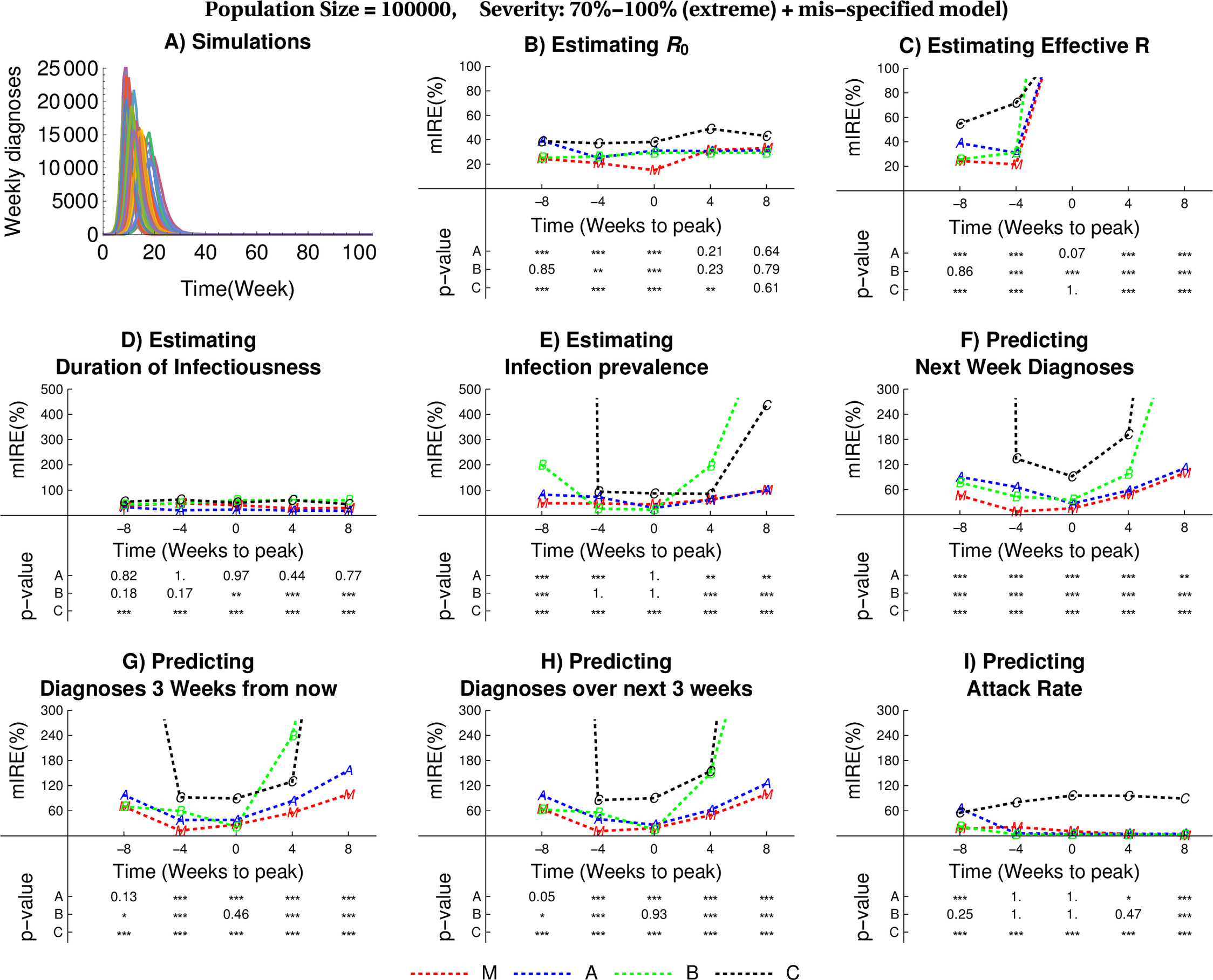

Supplement: S10 Fig — Same setting as in Fig 4. (TIF) [file pcbi.1005257.s011.tif]

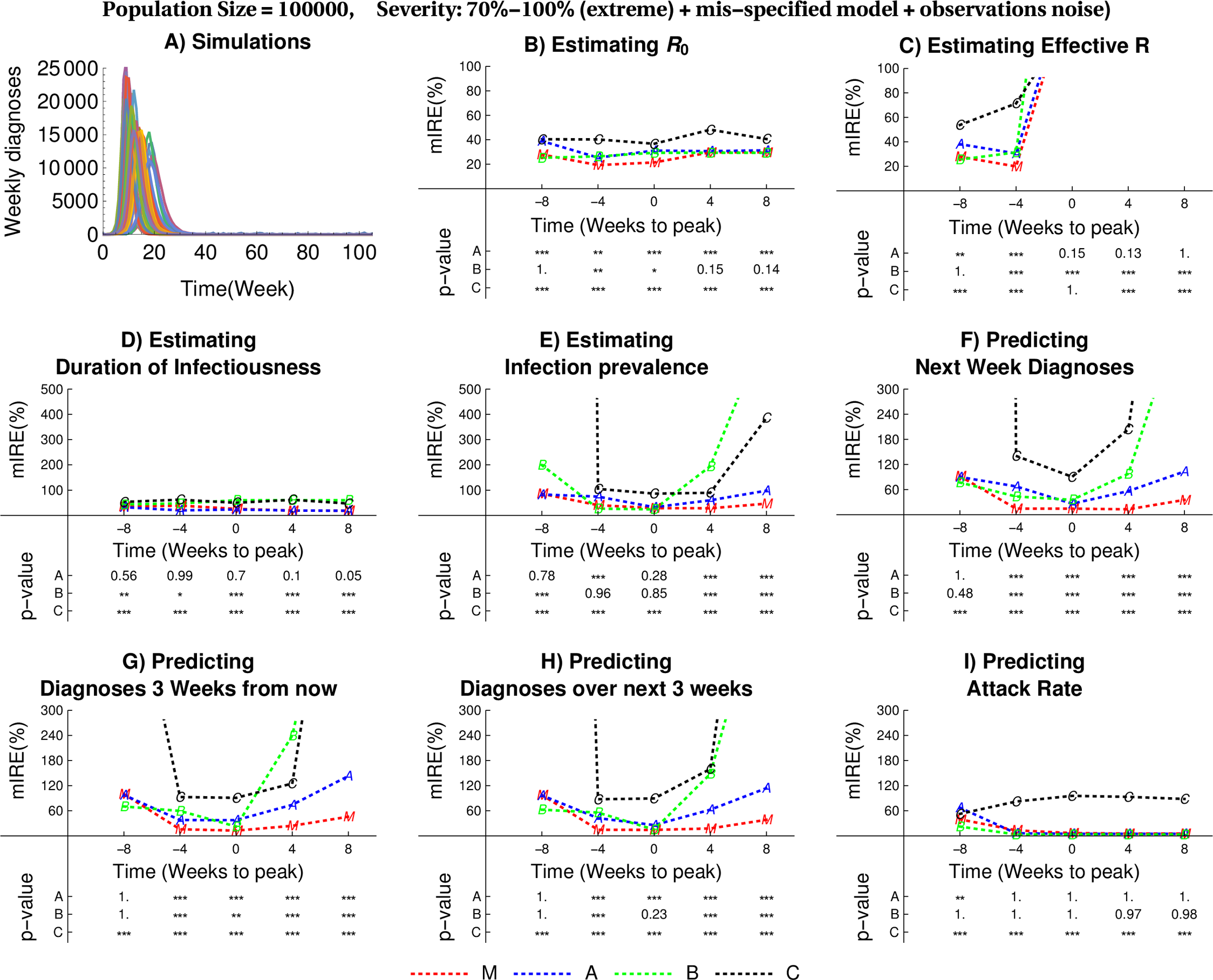

Supplement: S11 Fig — Same setting as in Fig 4. (TIF) [file pcbi.1005257.s012.tif]

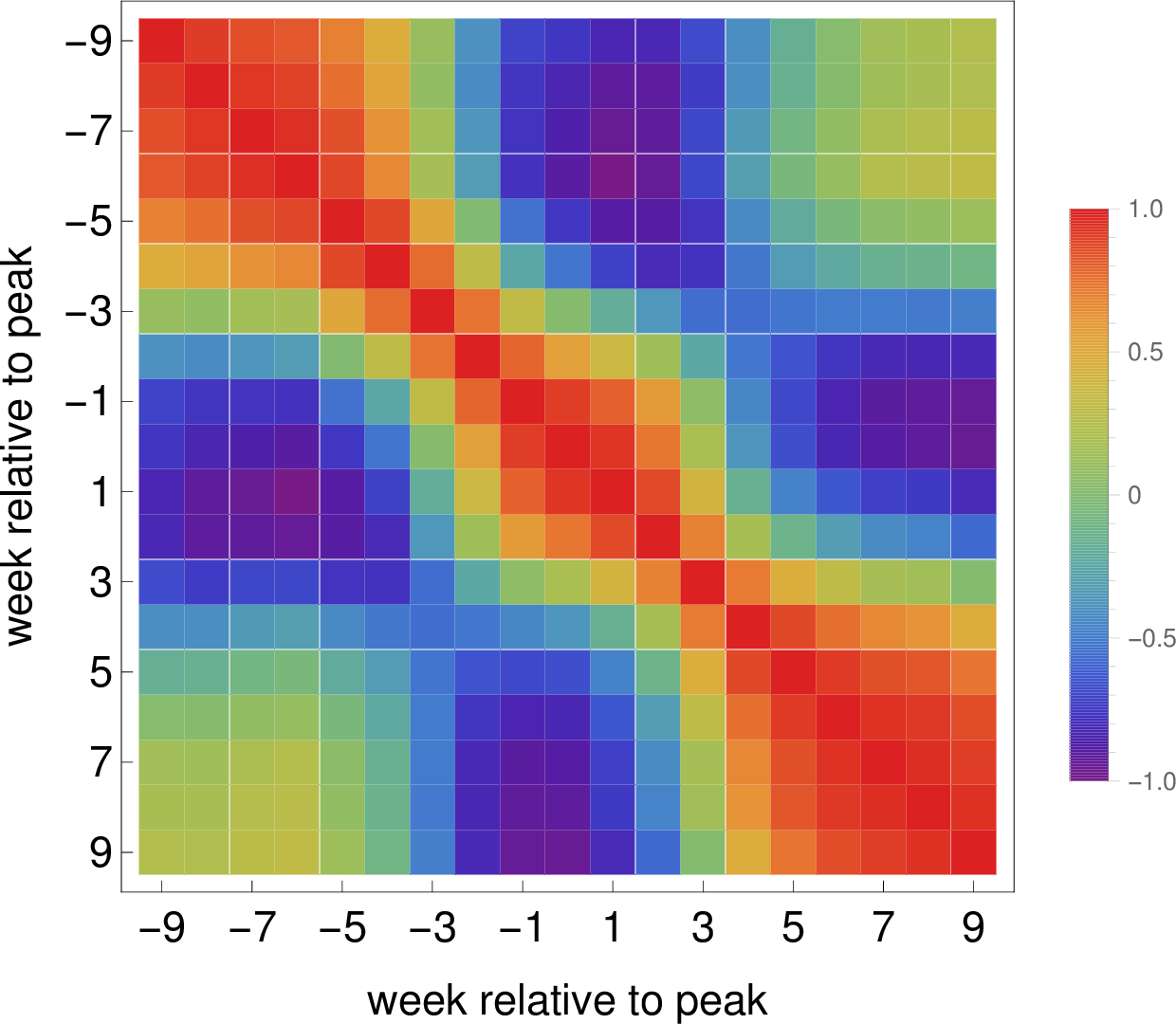

Supplement: S12 Fig — (TIF) [file pcbi.1005257.s013.tif]

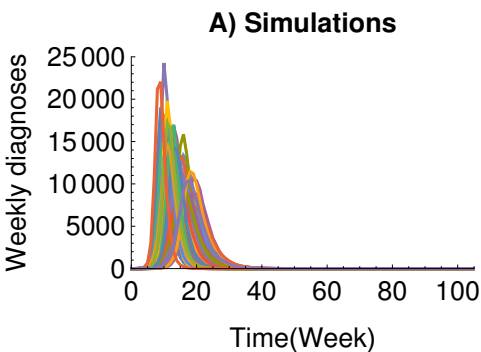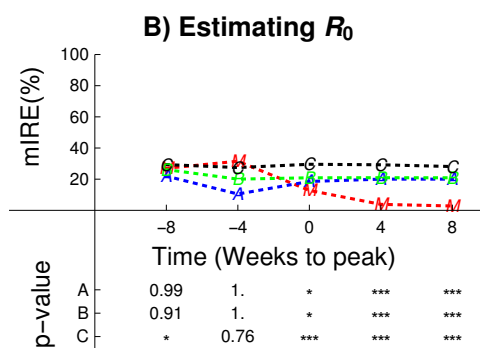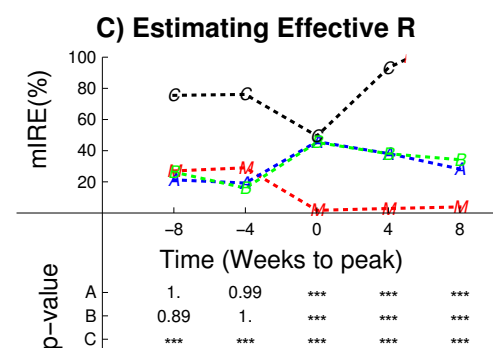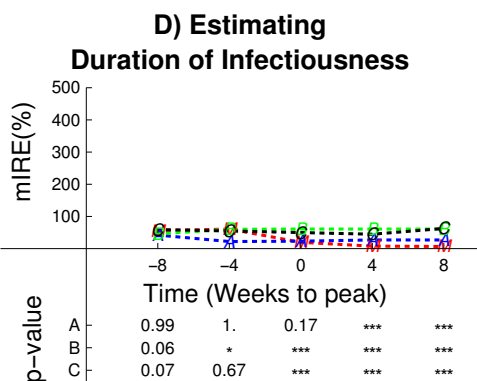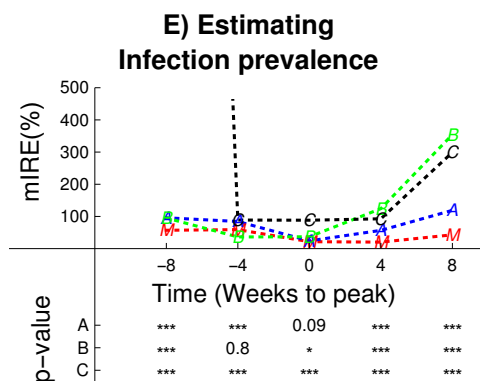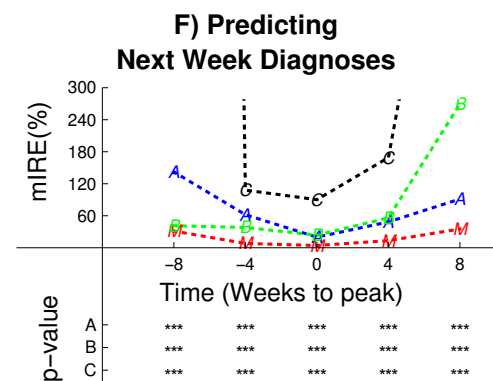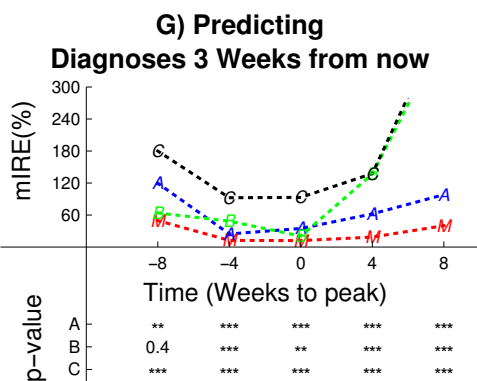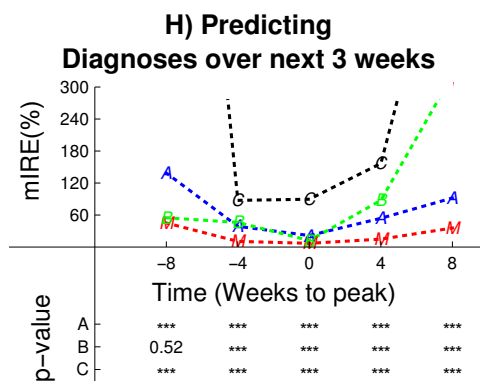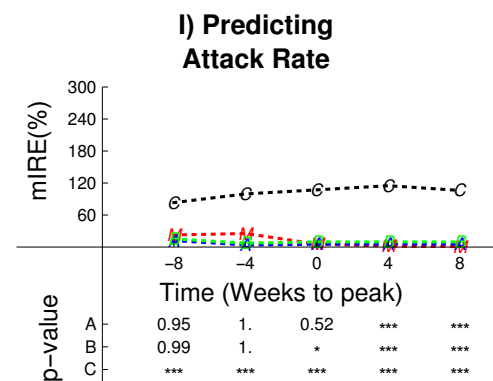

--- M --- A --- B --- C

Supplement: S1 File — (TAR.GZ) [file pcbi.1005257.s014.tar.gz › HSPH_Online-SI-Revision/output/S8Fig_n100-extreme.pdf]

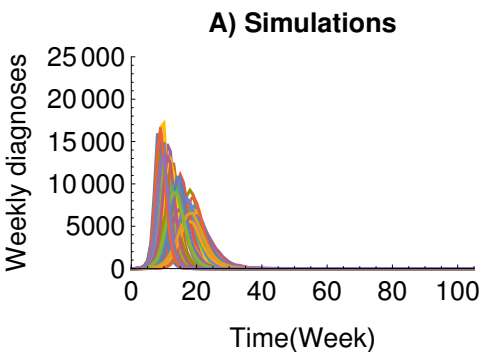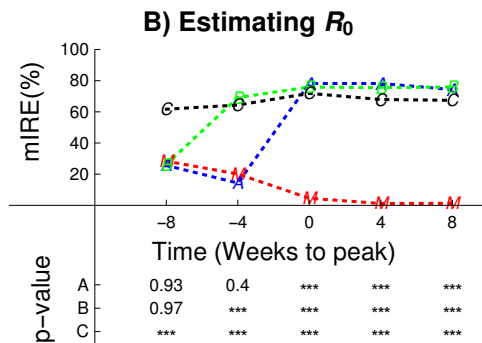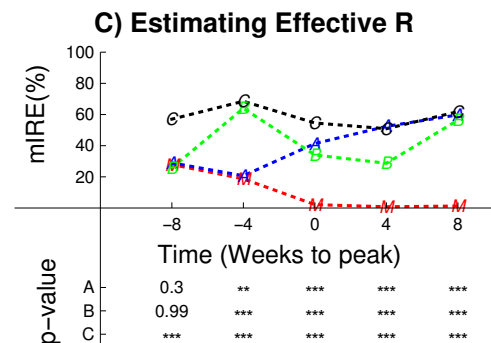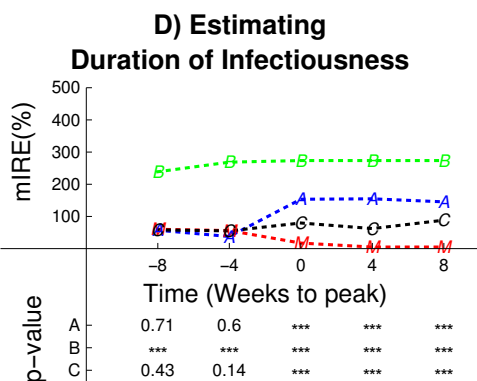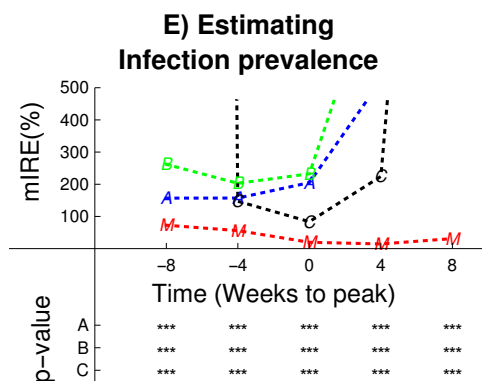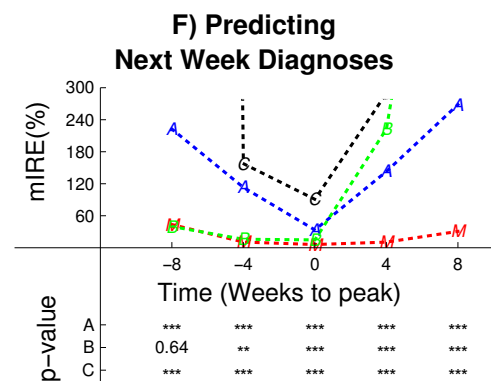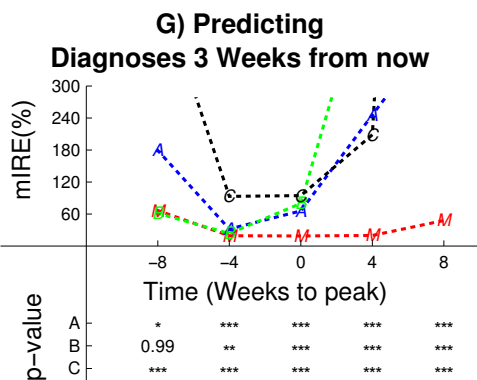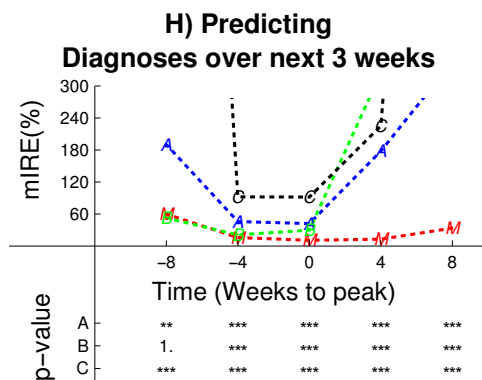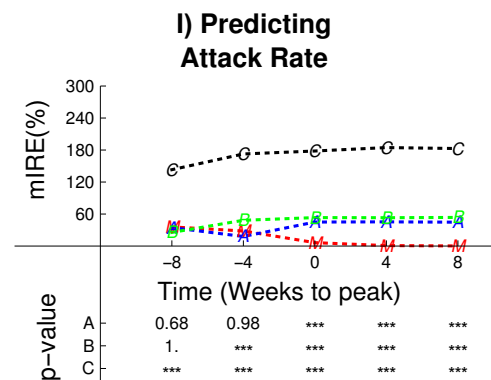

--- M --- A --- B --- C

Supplement: S1 File — (TAR.GZ) [file pcbi.1005257.s014.tar.gz › HSPH_Online-SI-Revision/output/S5Fig_n100-severe.pdf]
