# Supplementary material for: A Likelihood Approach for Real-Time Calibration of Stochastic Compartmental Epidemic Models
Source: PLoS Comput Biol. 2017 Jan 17;13(1):e1005257. doi: 10.1371/journal.pcbi.1005257 (PMC5240920; doi:10.1371/journal.pcbi.1005257)

A) For simulated epidemics with 30%–50% attack rate

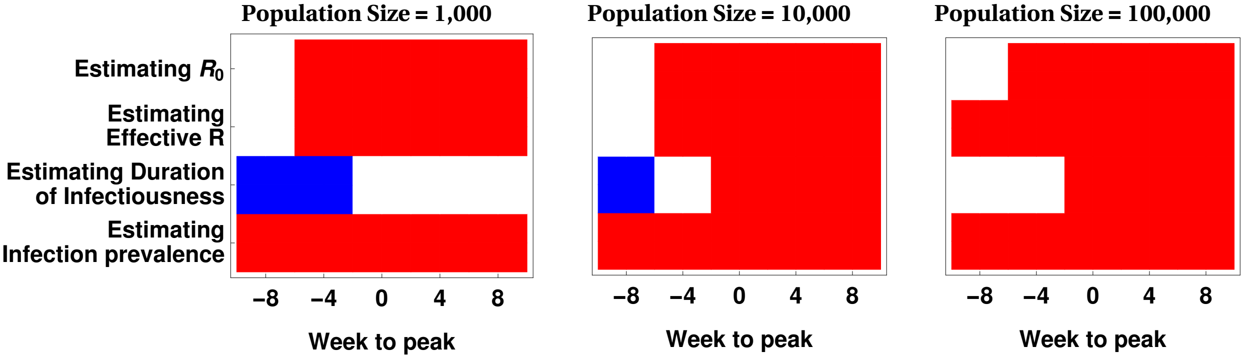


B) For simulated epidemics with 50% – 70% attack rate

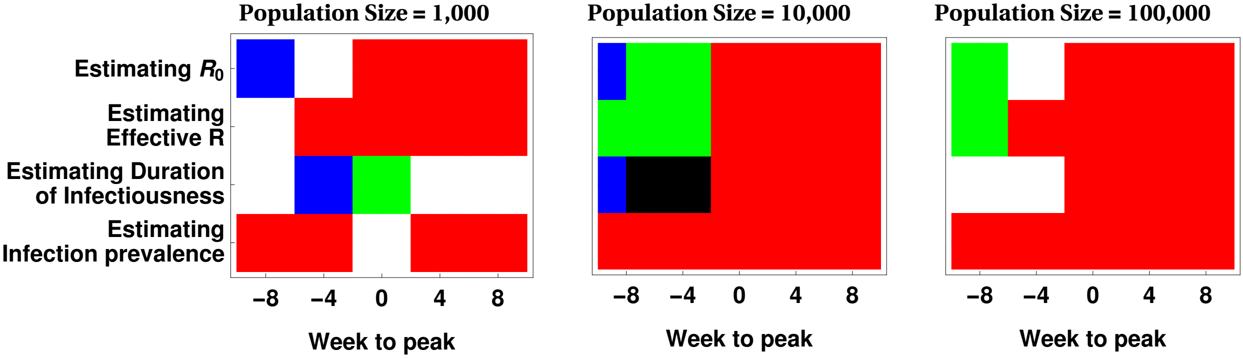


C) For simulated epidemics with 70% – 100% attack rate

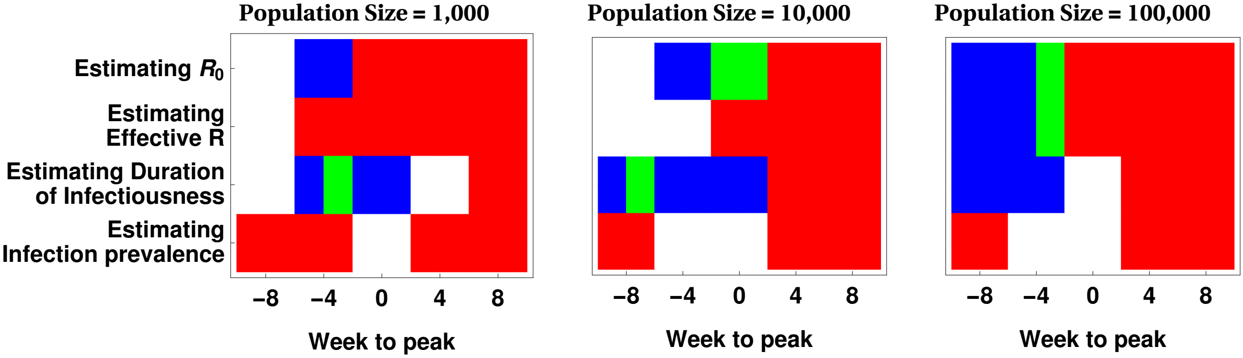


D) For simulated epidemics with 70% – 100% attack rate
left: observation noise, center: mis–specified model, right: noise + mis–specified model

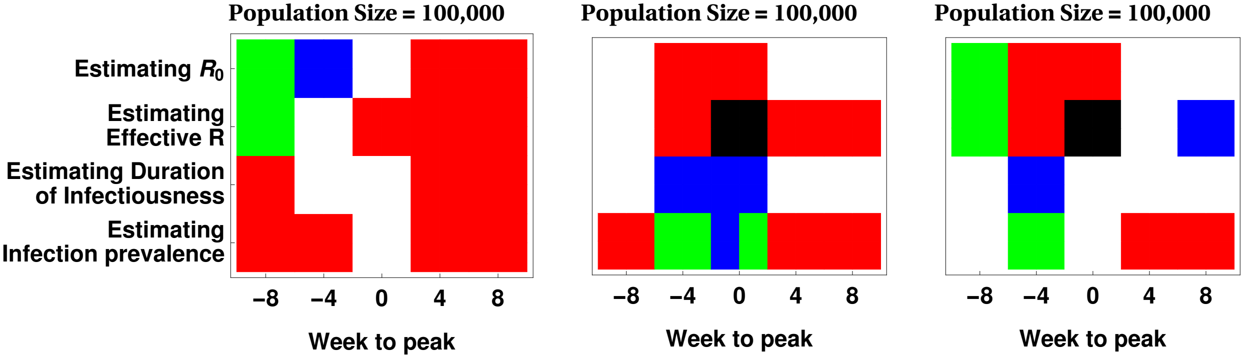

Supplement: S1 File — (TAR.GZ) [file pcbi.1005257.s014.tar.gz › HSPH_Online-SI-Revision/output/pv-estimation.pdf]
