# Supplementary material for: A Likelihood Approach for Real-Time Calibration of Stochastic Compartmental Epidemic Models
Source: PLoS Comput Biol. 2017 Jan 17;13(1):e1005257. doi: 10.1371/journal.pcbi.1005257 (PMC5240920; doi:10.1371/journal.pcbi.1005257)
Supplement: S1 File — (TAR.GZ) [file pcbi.1005257.s014.tar.gz › HSPH_Online-SI-Revision/output/pv-prediction.pdf]

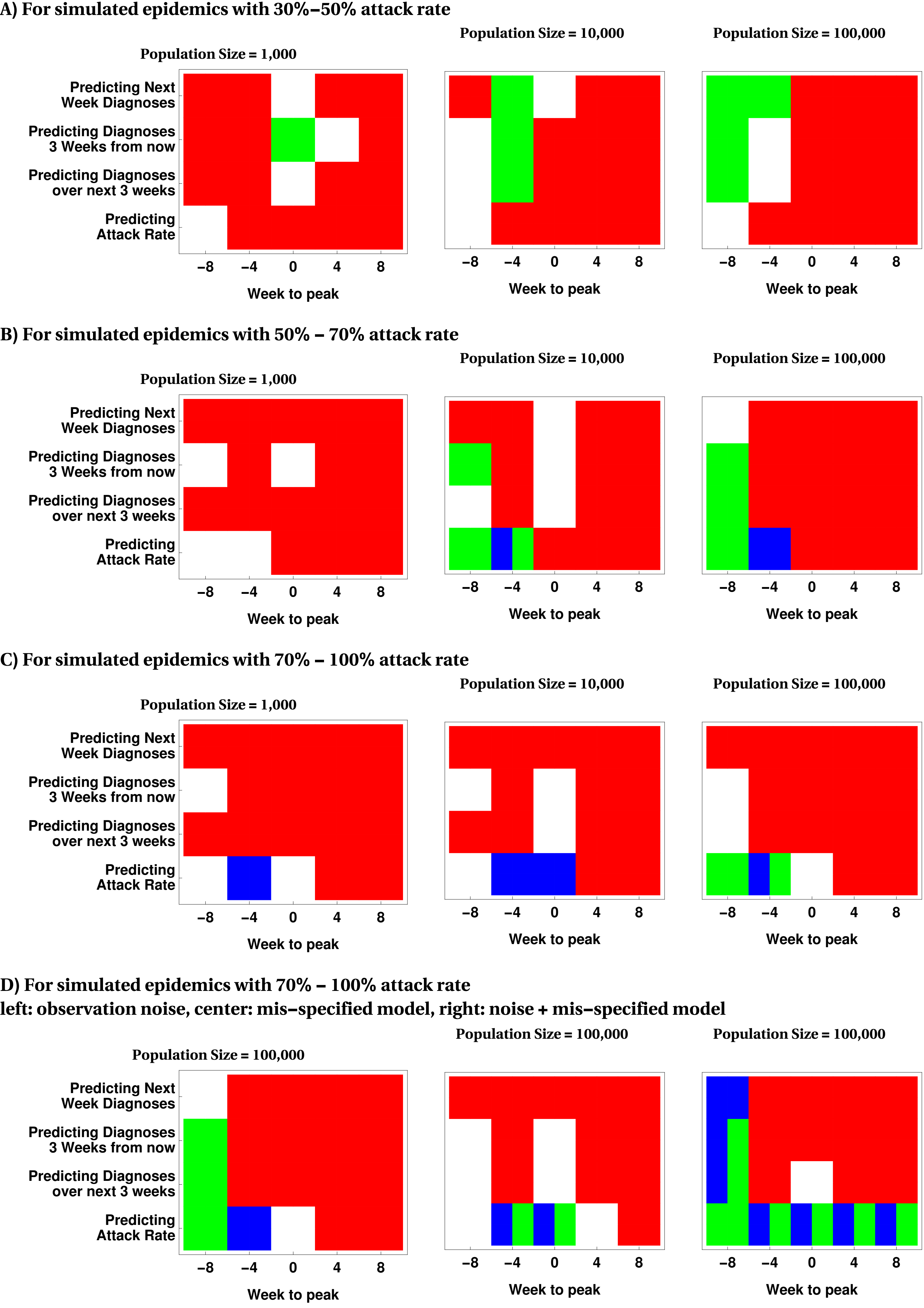
A) For simulated epidemics with 30%–50% attack rate
Population Size = 1,000
Population Size = 10,000
Population Size = 100,000
Predicting Next Week Diagnoses
Predicting Diagnoses 3 Weeks from now
Predicting Diagnoses over next 3 weeks
Predicting Attack Rate
−8 −4 0 4 8
Week to peak
B) For simulated epidemics with 50% – 70% attack rate
Population Size = 1,000
Population Size = 10,000
Population Size = 100,000
Predicting Next Week Diagnoses
Predicting Diagnoses 3 Weeks from now
Predicting Diagnoses over next 3 weeks
Predicting Attack Rate
−8 −4 0 4 8
Week to peak
C) For simulated epidemics with 70% – 100% attack rate
Population Size = 1,000
Population Size = 10,000
Population Size = 100,000
Predicting Next Week Diagnoses
Predicting Diagnoses 3 Weeks from now
Predicting Diagnoses over next 3 weeks
Predicting Attack Rate
−8 −4 0 4 8
Week to peak
D) For simulated epidemics with 70% – 100% attack rate
left: observation noise, center: mis–specified model, right: noise + mis–specified model
Population Size = 100,000
Population Size = 100,000
Population Size = 100,000
Predicting Next Week Diagnoses
Predicting Diagnoses 3 Weeks from now
Predicting Diagnoses over next 3 weeks
Predicting Attack Rate
−8 −4 0 4 8
Week to peak
