# Supplementary material for: A Likelihood Approach for Real-Time Calibration of Stochastic Compartmental Epidemic Models
Source: PLoS Comput Biol. 2017 Jan 17;13(1):e1005257. doi: 10.1371/journal.pcbi.1005257 (PMC5240920; doi:10.1371/journal.pcbi.1005257)
Supplement: S1 File — (TAR.GZ) [file pcbi.1005257.s014.tar.gz › HSPH_Online-SI-Revision/output/S2Fig_n10-mild.pdf]

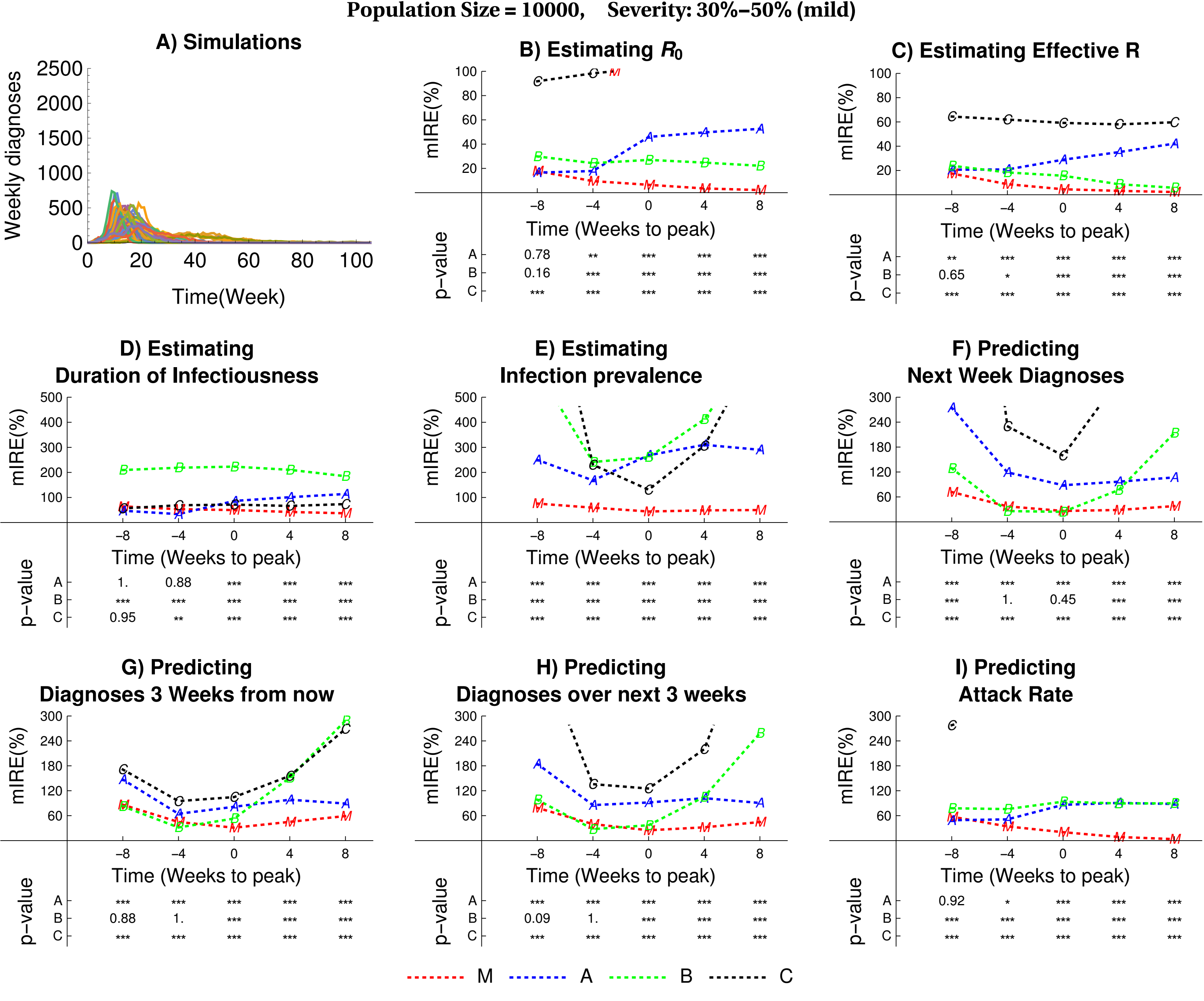

Population Size = 10000, Severity: 30%–50% (mild)
A) Simulations
Weekly diagnoses
Time(Week)
B) Estimating R_0
C) Estimating Effective R
D) Estimating Duration of Infectiousness
E) Estimating Infection prevalence
F) Predicting Next Week Diagnoses
G) Predicting Diagnoses 3 Weeks from now
H) Predicting Diagnoses over next 3 weeks
I) Predicting Attack Rate
mIRE(%)
Time (Weeks to peak)
p–value
B) p-value: A 0.78 ** *** *** ***; B 0.16 *** *** *** ***; C *** *** *** *** ***
C) p-value: A ** *** *** *** ***; B 0.65 * *** *** ***; C *** *** *** *** ***
D) p-value: A 1. 0.88 *** *** ***; B *** *** *** *** ***; C 0.95 ** *** *** ***
E) p-value: A *** *** *** *** ***; B *** *** *** *** ***; C *** *** *** *** ***
F) p-value: A *** *** *** *** ***; B *** 1. 0.45 *** ***; C *** *** *** *** ***
G) p-value: A *** *** *** *** ***; B 0.88 1. *** *** ***; C *** *** *** *** ***
H) p-value: A *** *** *** *** ***; B 0.09 1. *** *** ***; C *** *** *** *** ***
I) p-value: A 0.92 * *** *** ***; B *** *** *** *** ***; C *** *** *** *** ***
M A B C
