# Supplementary material for: A Likelihood Approach for Real-Time Calibration of Stochastic Compartmental Epidemic Models
Source: PLoS Comput Biol. 2017 Jan 17;13(1):e1005257. doi: 10.1371/journal.pcbi.1005257 (PMC5240920; doi:10.1371/journal.pcbi.1005257)
Supplement: S1 File — (TAR.GZ) [file pcbi.1005257.s014.tar.gz › HSPH_Online-SI-Revision/output/S9Fig_n100-extreme_noisy.pdf]

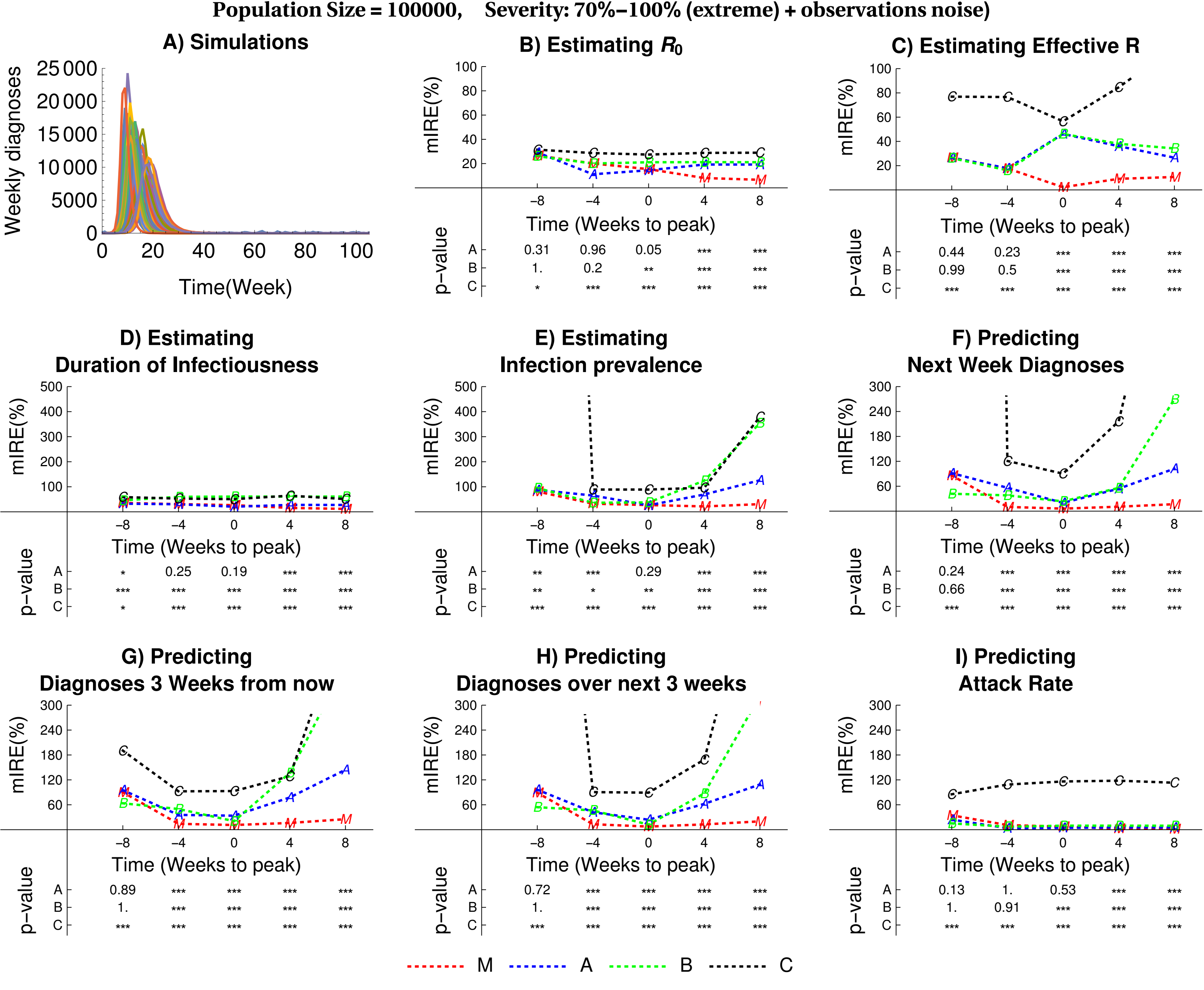
Population Size = 100000, Severity: 70%–100% (extreme) + observations noise
A) Simulations
Weekly diagnoses
Time(Week)
B) Estimating R_0
C) Estimating Effective R
D) Estimating Duration of Infectiousness
E) Estimating Infection prevalence
F) Predicting Next Week Diagnoses
G) Predicting Diagnoses 3 Weeks from now
H) Predicting Diagnoses over next 3 weeks
I) Predicting Attack Rate
mIRE(%)
Time (Weeks to peak)
p–value
M
A
B
C
