# Supplementary material for: A Likelihood Approach for Real-Time Calibration of Stochastic Compartmental Epidemic Models
Source: PLoS Comput Biol. 2017 Jan 17;13(1):e1005257. doi: 10.1371/journal.pcbi.1005257 (PMC5240920; doi:10.1371/journal.pcbi.1005257)
Supplement: S1 File — (TAR.GZ) [file pcbi.1005257.s014.tar.gz › HSPH_Online-SI-Revision/output/S4Fig_n1-severe.pdf]

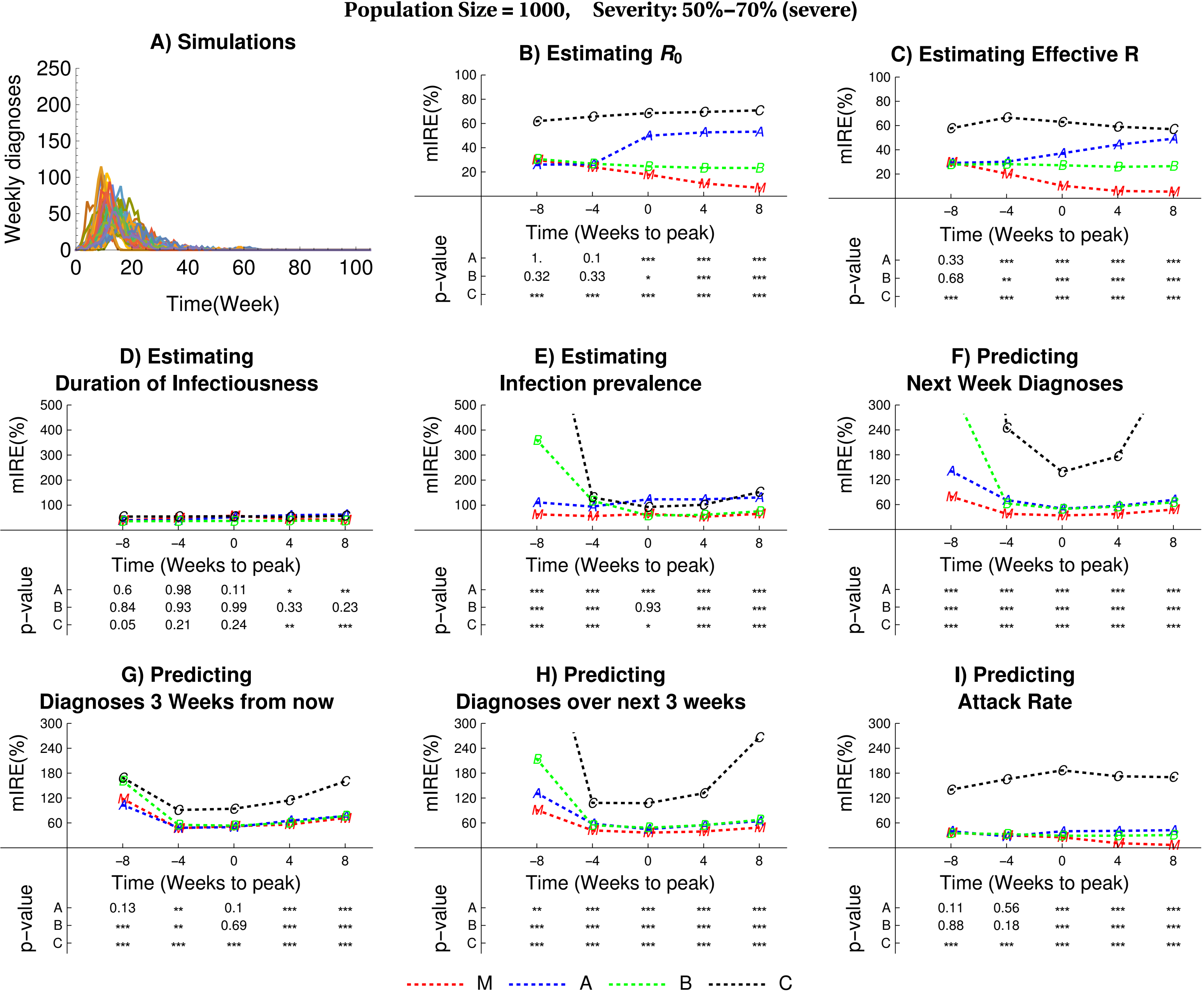

Panel	Row	−8	−4	0	4	8
B	A	1.	0.1	***	***	***
B	B	0.32	0.33	*	***	***
B	C	***	***	***	***	***
C	A	0.33	***	***	***	***
C	B	0.68	**	***	***	***
C	C	***	***	***	***	***
D	A	0.6	0.98	0.11	*	**
D	B	0.84	0.93	0.99	0.33	0.23
D	C	0.05	0.21	0.24	**	***
E	A	***	***	***	***	***
E	B	***	***	0.93	***	***
E	C	***	***	*	***	***
F	A	***	***	***	***	***
F	B	***	***	***	***	***
F	C	***	***	***	***	***
G	A	0.13	**	0.1	***	***
G	B	***	**	0.69	***	***
G	C	***	***	***	***	***
H	A	**	***	***	***	***
H	B	***	***	***	***	***
H	C	***	***	***	***	***
I	A	0.11	0.56	***	***	***
I	B	0.88	0.18	***	***	***
I	C	***	***	***	***	***
