# Supplementary material for: A Likelihood Approach for Real-Time Calibration of Stochastic Compartmental Epidemic Models
Source: PLoS Comput Biol. 2017 Jan 17;13(1):e1005257. doi: 10.1371/journal.pcbi.1005257 (PMC5240920; doi:10.1371/journal.pcbi.1005257)
Supplement: S1 File — (TAR.GZ) [file pcbi.1005257.s014.tar.gz › HSPH_Online-SI-Revision/MSS10/n10-extreme/n10-extreme_NEW2_table8.pdf]

1	newly 3 weeks spec	33.	14.59	{9., 19.}	55.7879	0.370642	newly 3 weeks cumul	135.	70.26	{63., 78.}	47.9556	0.285239	newly 5 weeks spec	7.	5.29	{2., 8.}	33.2857	0.223485	newly 5 weeks cumul	159.	84.37	{74., 95.}	46.9371	0.277041	newly 50 weeks cumul	163.	94.85	{82., 105.}	41.8098	0.236899
2	newly 3 weeks spec	108.	88.98	{77., 102.}	18.5	0.0913499	newly 3 weeks cumul	418.	397.69	{368., 429.}	5.97847	0.0271204	newly 5 weeks spec	44.	41.52	{29., 52.}	16.0909	0.0766702	newly 5 weeks cumul	535.	499.83	{461., 537.}	7.41495	0.0340138	newly 46 weeks cumul	683.	585.29	{528., 644.}	14.5051	0.0692584
3	newly 3 weeks spec	12.	13.51	{8., 19.}	29.9167	0.128083	newly 3 weeks cumul	49.	73.36	{62., 82.}	49.7143	0.172818	newly 5 weeks spec	3.	4.34	{2., 8.}	68.6667	0.258218	newly 5 weeks cumul	60.	85.76	{72., 96.}	42.9333	0.152435	newly 52 weeks cumul	67.	92.83	{79., 107.}	38.6418	0.139045
4	newly 3 weeks spec	57.	42.04	{33., 50.}	26.3158	0.138022	newly 3 weeks cumul	214.	184.54	{171., 197.}	13.8411	0.0652886	newly 5 weeks spec	34.	20.9	{16., 27.}	38.5294	0.22164	newly 5 weeks cumul	292.	235.62	{220., 253.}	19.3082	0.0937705	newly 46 weeks cumul	396.	288.35	{264., 311.}	27.1843	0.138528
5	newly 3 weeks spec	61.	51.99	{44., 60.}	15.5246	0.0762118	newly 3 weeks cumul	281.	224.06	{213., 235.}	20.2633	0.0986329	newly 5 weeks spec	29.	27.85	{22., 36.}	14.5862	0.0661295	newly 5 weeks cumul	366.	289.41	{280., 299.}	20.9262	0.102143	newly 49 weeks cumul	445.	362.55	{347., 379.}	18.5281	0.0892431
6	newly 3 weeks spec	52.	46.13	{39., 56.}	14.2885	0.0687708	newly 3 weeks cumul	218.	204.95	{195., 216.}	6.22477	0.0282301	newly 5 weeks spec	24.	24.02	{16., 30.}	17.3333	0.0775532	newly 5 weeks cumul	283.	262.2	{250., 274.}	7.42049	0.0337723	newly 50 weeks cumul	342.	320.7	{305., 340.}	6.39766	0.029027
7	newly 3 weeks spec	45.	76.48	{66., 90.}	69.9556	0.226975	newly 3 weeks cumul	222.	329.45	{301., 353.}	48.4009	0.170724	newly 5 weeks spec	26.	38.16	{28., 46.}	47.1538	0.160947	newly 5 weeks cumul	276.	422.32	{395., 445.}	53.0145	0.184267	newly 51 weeks cumul	350.	517.2	{481., 548.}	47.7714	0.169093
8	newly 3 weeks spec	74.	76.54	{66., 85.}	9.32432	0.0397452	newly 3 weeks cumul	355.	336.86	{322., 354.}	5.24507	0.0236679	newly 5 weeks spec	40.	37.08	{30., 44.}	13.5	0.0640579	newly 5 weeks cumul	452.	428.56	{414., 443.}	5.25221	0.0235765	newly 48 weeks cumul	577.	515.27	{494., 537.}	10.7019	0.0493641
9	newly 3 weeks spec	75.	67.07	{56., 77.}	11.72	0.0559801	newly 3 weeks cumul	323.	275.55	{261., 290.}	14.6904	0.0693449	newly 5 weeks spec	31.	35.95	{29., 43.}	19.9032	0.0774017	newly 5 weeks cumul	408.	360.21	{341., 378.}	11.7132	0.0543723	newly 48 weeks cumul	485.	453.9	{431., 474.}	6.44536	0.0292677
10	newly 3 weeks spec	97.	97.25	{86., 110.}	7.70103	0.0334263	newly 3 weeks cumul	429.	390.06	{372., 409.}	9.07692	0.0415793	newly 5 weeks spec	65.	57.35	{46., 69.}	15.2769	0.0737985	newly 5 weeks cumul	590.	522.07	{501., 545.}	11.5136	0.0533396	newly 48 weeks cumul	747.	699.85	{673., 723.}	6.33601	0.0285986
11	newly 3 weeks spec	96.	16.43	{12., 22.}	82.8854	0.783113	newly 3 weeks cumul	365.	81.22	{69., 92.}	77.7479	0.655527	newly 5 weeks spec	43.	6.96	{3., 12.}	83.814	0.862859	newly 5 weeks cumul	474.	98.77	{82., 113.}	79.1624	0.684453	newly 43 weeks cumul	637.	111.85	{90., 131.}	82.4411	0.759733
12	newly 3 weeks spec	56.	132.73	{118., 145.}	137.018	0.373241	newly 3 weeks cumul	258.	525.61	{508., 544.}	103.725	0.308885	newly 5 weeks spec	30.	83.1	{70., 97.}	177.	0.439359	newly 5 weeks cumul	326.	715.85	{691., 740.}	119.586	0.341458	newly 48 weeks cumul	370.	992.32	{960., 1024.}	168.195	0.428314
13	newly 3 weeks spec	46.	95.99	{81., 108.}	108.674	0.316947	newly 3 weeks cumul	235.	371.74	{352., 390.}	58.1872	0.198833	newly 5 weeks spec	40.	58.86	{47., 70.}	47.25	0.163493	newly 5 weeks cumul	321.	506.23	{482., 527.}	57.704	0.197531	newly 44 weeks cumul	406.	724.96	{680., 768.}	78.5616	0.251373
14	newly 3 weeks spec	50.	54.04	{43., 68.}	14.44	0.0575376	newly 3 weeks cumul	229.	229.54	{209., 267.}	6.73362	0.0286348	newly 5 weeks spec	25.	27.64	{20., 36.}	21.36	0.0852572	newly 5 weeks cumul	291.	295.04	{269., 356.}	6.68041	0.0277491	newly 49 weeks cumul	352.	366.41	{327., 458.}	8.26989	0.0332569
15	newly 3 weeks spec	41.	14.05	{6., 22.}	65.7317	0.510021	newly 3 weeks cumul	175.	72.71	{56., 91.}	58.4514	0.390941	newly 5 weeks spec	20.	5.66	{2., 10.}	71.7	0.642718	newly 5 weeks cumul	229.	86.81	{67., 109.}	62.0917	0.430493	newly 46 weeks cumul	253.	95.38	{70., 121.}	62.3004	0.434142
16	newly 3 weeks spec	68.	89.6	{79., 98.}	31.7647	0.117742	newly 3 weeks cumul	284.	388.99	{372., 406.}	36.9683	0.136387	newly 5 weeks spec	35.	44.23	{36., 52.}	27.9714	0.104298	newly 5 weeks cumul	368.	493.89	{479., 508.}	34.2092	0.127608	newly 49 weeks cumul	425.	598.88	{577., 622.}	40.9129	0.148752
17	newly 3 weeks spec	58.	65.93	{55., 74.}	15.7759	0.0626239	newly 3 weeks cumul	228.	299.96	{286., 315.}	31.5614	0.118819	newly 5 weeks spec	27.	31.08	{22., 39.}	22.8148	0.0890484	newly 5 weeks cumul	296.	377.86	{360., 392.}	27.6554	0.105797	newly 51 weeks cumul	355.	447.01	{427., 467.}	25.9183	0.0998123
18	newly 3 weeks spec	58.	67.26	{56., 78.}	17.3448	0.0674771	newly 3 weeks cumul	249.	291.51	{279., 306.}	17.0723	0.0681891	newly 5 weeks spec	33.	34.83	{27., 42.}	15.1212	0.0635514	newly 5 weeks cumul	331.	374.73	{364., 385.}	13.2115	0.0537598	newly 49 weeks cumul	406.	456.89	{444., 468.}	12.5345	0.0511606
19	newly 3 weeks spec	19.	61.62	{51., 71.}	224.316	0.506962	newly 3 weeks cumul	129.	295.91	{280., 312.}	129.388	0.360158	newly 5 weeks spec	10.	26.18	{19., 33.}	161.8	0.407225	newly 5 weeks cumul	154.	361.71	{340., 384.}	134.877	0.3704	newly 51 weeks cumul	172.	406.04	{382., 432.}	136.07	0.372575
20	newly 3 weeks spec	82.	73.23	{62., 83.}	12.2561	0.0583853	newly 3 weeks cumul	349.	324.51	{307., 342.}	7.08023	0.0322242	newly 5 weeks spec	51.	34.91	{28., 43.}	31.549	0.170545	newly 5 weeks cumul	464.	410.42	{394., 428.}	11.5474	0.0535103	newly 48 weeks cumul	599.	488.07	{465., 510.}	18.5192	0.0892205
21	newly 3 weeks spec	71.	86.58	{75., 100.}	22.6197	0.0865189	newly 3 weeks cumul	319.	402.17	{387., 421.}	26.0721	0.100406	newly 5 weeks spec	24.	38.49	{31., 47.}	60.7917	0.200659	newly 5 weeks cumul	400.	498.98	{478., 518.}	24.745	0.0958375	newly 45 weeks cumul	509.	577.47	{552., 603.}	13.4519	0.0545592
22	newly 3 weeks spec	15.	18.32	{12., 24.}	30.	0.113851	newly 3 weeks cumul	86.	99.46	{91., 107.}	15.6977	0.0625206	newly 5 weeks spec	10.	6.48	{3., 10.}	38.4	0.242643	newly 5 weeks cumul	114.	116.17	{106., 127.}	5.57018	0.0237476	newly 52 weeks cumul	122.	125.44	{115., 138.}	5.95082	0.0251541
23	newly 3 weeks spec	49.	198.75	{180., 215.}	305.612	0.606977	newly 3 weeks cumul	175.	730.87	{704., 758.}	317.64	0.620628	newly 5 weeks spec	26.	128.6	{113., 145.}	394.615	0.691921	newly 5 weeks cumul	236.	1022.06	{987., 1063.}	333.076	0.636382	newly 47 weeks cumul	286.	1513.32	{1452., 1585.}	429.133	0.723323
24	newly 3 weeks spec	62.	100.23	{89., 111.}	61.6613	0.206859	newly 3 weeks cumul	297.	407.71	{389., 424.}	37.2761	0.137382	newly 5 weeks spec	38.	55.98	{46., 64.}	47.3158	0.164955	newly 5 weeks cumul	391.	537.91	{523., 557.}	37.5729	0.138412	newly 45 weeks cumul	543.	709.29	{689., 729.}	30.6243	0.11592
25	newly 3 weeks spec	17.	8.04	{4., 12.}	52.7059	0.371034	newly 3 weeks cumul	104.	48.21	{42., 55.}	53.6442	0.336668	newly 5 weeks spec	9.	2.81	{0., 5.}	68.7778	0.691926	newly 5 weeks cumul	127.	55.07	{46., 66.}	56.6378	0.365942	newly 48 weeks cumul	139.	58.36	{48., 69.}	58.0144	0.380785
26	newly 3 weeks spec	8.	2.08	{0., 5.}	83.25	1.07265	newly 3 weeks cumul	30.	13.04	{4., 20.}	67.6667	0.512426	newly 5 weeks spec	1.	0.46	{0., 1.}	110.	0.865843	newly 5 weeks cumul	36.	14.56	{4., 22.}	70.6111	0.557233	newly 51 weeks cumul	40.	15.17	{4., 22.}	73.325	0.596114
27	newly 3 weeks spec	65.	60.95	{51., 71.}	10.4769	0.0484279	newly 3 weeks cumul	303.	265.86	{251., 278.}	12.2574	0.057166	newly 5 weeks spec	45.	30.65	{24., 38.}	32.2889	0.177437	newly 5 weeks cumul	394.	339.87	{322., 357.}	13.7386	0.0645224	newly 49 weeks cumul	475.	414.83	{390., 435.}	12.6674	0.059167
28	newly 3 weeks spec	25.	18.64	{12., 25.}	26.8	0.148619	newly 3 weeks cumul	120.	95.85	{86., 104.}	20.125	0.0987274	newly 5 weeks spec	9.	7.79	{4., 12.}	31.8889	0.172972	newly 5 weeks cumul	146.	115.84	{106., 125.}	20.6575	0.101386	newly 52 weeks cumul	160.	128.61	{116., 140.}	19.6188	0.0958887
29	newly 3 weeks spec	36.	84.56	{74., 97.}	134.889	0.36826	newly 3 weeks cumul	170.	354.29	{339., 367.}	108.406	0.318698	newly 5 weeks spec	12.	45.61	{37., 53.}	280.083	0.575074	newly 5 weeks cumul	201.	461.73	{445., 477.}	129.716	0.361056	newly 47 weeks cumul	219.	588.19	{570., 606.}	168.58	0.428934
30	newly 3 weeks spec	50.	36.88	{27., 45.}	26.6	0.140611	newly 3 weeks cumul	199.	177.78	{162., 191.}	10.8643	0.050762	newly 5 weeks spec	23.	17.06	{11., 24.}	29.2174	0.16254	newly 5 weeks cumul	254.	220.36	{201., 241.}	13.2598	0.0628136	newly 47 weeks cumul	304.	252.98	{223., 282.}	16.7829	0.0813476
31	newly 3 weeks spec	53.	50.35	{41., 57.}	11.2264	0.0512101	newly 3 weeks cumul	252.	222.27	{209., 235.}	11.8452	0.055228	newly 5 weeks spec	36.	25.51	{19., 31.}	29.5833	0.161217	newly 5 weeks cumul	334.	282.81	{266., 298.}	15.3263	0.0726643	newly 50 weeks cumul	377.	340.24	{318., 357.}	9.75066	0.0449425
32	newly 3 weeks spec	76.	103.26	{90., 117.}	35.8684	0.130913	newly 3 weeks cumul	281.	408.91	{392., 426.}	45.5196	0.162703	newly 5 weeks spec	36.	60.34	{50., 71.}	67.6111	0.220259	newly 5 weeks cumul	374.	547.32	{526., 569.}	46.3422	0.165185	newly 42 weeks cumul	533.	747.91	{716., 779.}	40.3208	0.146921
33	newly 3 weeks spec	8.	20.31	{14., 26.}	153.875	0.392696	newly 3 weeks cumul	52.	105.07	{97., 113.}	102.058	0.304666	newly 5 weeks spec	2.	8.66	{5., 12.}	333.	0.61025	newly 5 weeks cumul	59.	127.46	{118., 137.}	116.034	0.333854	newly 50 weeks cumul	62.	141.74	{131., 154.}	128.613	0.358265
34	newly 3 weeks spec	8.	21.54	{14., 30.}	169.75	0.412892	newly 3 weeks cumul	61.	120.35	{106., 134.}	97.2951	0.293175	newly 5 weeks spec	7.	6.73	{2., 11.}	40.4286	0.211269	newly 5 weeks cumul	76.	138.73	{120., 157.}	82.5395	0.259009	newly 52 weeks cumul	80.	147.6	{128., 169.}	84.5	0.263253
35	newly 3 weeks spec	95.	41.77	{33., 50.}	56.0316	0.362477	newly 3 weeks cumul	347.	178.41	{163., 196.}	48.585	0.290023	newly 5 weeks spec	52.	23.3	{15., 31.}	55.1923	0.363166	newly 5 weeks cumul	464.	233.62	{215., 255.}	49.6509	0.299082	newly 47 weeks cumul	569.	297.7	{264., 334.}	47.6801	0.283056
36	newly 3 weeks spec	110.	114.99	{102., 126.}	7.91818	0.0328775	newly 3 weeks cumul	389.	503.94	{487., 521.}	29.5476	0.112263	newly 5 weeks spec	47.	59.2	{49., 70.}	26.4255	0.0988816	newly 5 weeks cumul	515.	644.96	{626., 664.}	25.235	0.0976278	newly 45 weeks cumul	717.	787.68	{769., 808.}	9.85774	0.0407534
37	newly 3 weeks spec	60.	22.38	{17., 28.}	62.7	0.438846	newly 3 weeks cumul	245.	97.17	{87., 108.}	60.3388	0.402966	newly 5 weeks spec	22.	11.22	{6., 16.}	49.	0.320106	newly 5 weeks cumul	316.	124.54	{113., 134.}	60.5886	0.405349	newly 48 weeks cumul	370.	152.68	{135., 166.}	58.7351	0.385771
38	newly 3 weeks spec	10.	0.49	{0., 2.}	95.1	1.61035	newly 3 weeks cumul	32.	2.98	{1., 5.}	90.6875	1.09222	newly 5 weeks spec	1.	0.13	{0., 1.}	91.	0.894771	newly 5 weeks cumul	36.	3.33	{1., 6.}	90.75	1.09186	newly 51 weeks cumul	37.	3.48	{1., 6.}	90.5946	1.08847
39	newly 3 weeks spec	14.	6.89	{4., 10.}	50.7857	0.337072	newly 3 weeks cumul	85.	32.8	{28., 38.}	61.4118	0.416304	newly 5 weeks spec	6.	3.	{1., 5.}	51.6667	0.446089	newly 5 weeks cumul	101.	40.24	{34., 47.}	60.1584	0.402888	newly 51 weeks cumul	112.	45.52	{38., 53.}	59.3571	0.394617
40	newly 3 weeks spec	73.	42.22	{35., 52.}	42.1644	0.244037	newly 3 weeks cumul	282.	179.98	{167., 192.}	36.1773	0.195799	newly 5 weeks spec	30.	22.96	{15., 30.}	25.	0.134404	newly 5 weeks cumul	357.	232.66	{215., 250.}	34.8291	0.186588	newly 48 weeks cumul	439.	290.63	{268., 313.}	33.7973	0.179894
41	newly 3 weeks spec	66.	47.24	{38., 56.}	28.4545	0.149812	newly 3 weeks cumul	240.	215.23	{202., 229.}	10.3208	0.0477383	newly 5 weeks spec	31.	22.01	{16., 28.}	29.4516	0.161739	newly 5 weeks cumul	309.	268.79	{253., 285.}	13.0129	0.0609492	newly 47 weeks cumul	381.	315.22	{297., 332.}	17.2651	0.0827531
42	newly 3 weeks spec	69.	62.06	{53., 71.}	12.3478	0.0588492	newly 3 weeks cumul	344.	269.87	{255., 286.}	21.5494	0.105839	newly 5 weeks spec	67.	32.5	{26., 39.}	51.4925	0.319562	newly 5 weeks cumul	474.	346.66	{330., 364.}	26.865	0.136171	newly 42 weeks cumul	655.	420.78	{398., 443.}	35.7588	0.192555
43	newly 3 weeks spec	15.	3.32	{1., 6.}	77.8667	0.778002	newly 3 weeks cumul	71.	19.57	{13., 26.}	72.4366	0.573068	newly 5 weeks spec	6.	0.96	{0., 2.}	84.	1.10108	newly 5 weeks cumul	88.	22.44	{15., 30.}	74.5	0.60808	newly 52 weeks cumul	93.	24.15	{15., 32.}	74.0323	0.602844
44	newly 3 weeks spec	89.	71.95	{60., 82.}	19.2472	0.0960617	newly 3 weeks cumul	399.	308.42	{293., 324.}	22.7018	0.112199	newly 5 weeks spec	51.	36.38	{28., 45.}	28.7059	0.153558	newly 5 weeks cumul	518.	396.59	{378., 415.}	23.4382	0.116279	newly 43 weeks cumul	705.	490.15	{461., 518.}	30.4752	0.158241
45	newly 3 weeks spec	84.	35.94	{28., 43.}	57.2143	0.374207	newly 3 weeks cumul	323.	154.83	{140., 167.}	52.065	0.320252	newly 5 weeks spec	39.	18.95	{14., 24.}	51.4103	0.332835	newly 5 weeks cumul	423.	201.04	{184., 217.}	52.4728	0.323972	newly 47 weeks cumul	539.	254.1	{230., 279.}	52.8571	0.327768
46	newly 3 weeks spec	113.	134.98	{119., 148.}	19.5929	0.076156	newly 3 weeks cumul	424.	568.81	{548., 587.}	34.1533	0.127438	newly 5 weeks spec	54.	76.92	{66., 87.}	42.4444	0.150774	newly 5 weeks cumul	553.	750.08	{727., 772.}	35.6383	0.132265	newly 45 weeks cumul	782.	960.27	{931., 987.}	22.7967	0.0890904
47	newly 3 weeks spec	79.	79.32	{69., 88.}	7.79747	0.0337984	newly 3 weeks cumul	324.	327.78	{314., 341.}	2.98765	0.0128743	newly 5 weeks spec	43.	42.83	{35., 51.}	12.3023	0.0542418	newly 5 weeks cumul	419.	431.33	{414., 447.}	3.51551	0.0149368	newly 47 weeks cumul	531.	559.3	{542., 575.}	5.37476	0.0226198
48	newly 3 weeks spec	58.	56.52	{45., 67.}	11.	0.0501301	newly 3 weeks cumul	234.	254.03	{242., 265.}	8.57692	0.0354511	newly 5 weeks spec	22.	27.99	{18., 37.}	33.3182	0.121087	newly 5 weeks cumul	292.	322.16	{309., 336.}	10.3288	0.0424151	newly 50 weeks cumul	344.	386.48	{369., 409.}	12.3663	0.0502353
49	newly 3 weeks spec	22.	42.42	{32., 52.}	92.8182	0.278503	newly 3 weeks cumul	126.	200.78	{187., 216.}	59.3492	0.201744	newly 5 weeks spec	10.	19.04	{13., 24.}	90.4	0.267343	newly 5 weeks cumul	151.	247.93	{231., 266.}	64.1921	0.214752	newly 51 weeks cumul	165.	284.05	{263., 302.}	72.1515	0.235228
50	newly 3 weeks spec	52.	79.36	{69., 90.}	52.6154	0.181412	newly 3 weeks cumul	223.	327.55	{313., 340.}	46.8834	0.166714	newly 5 weeks spec	29.	42.49	{32., 51.}	47.069	0.161965	newly 5 weeks cumul	274.	429.77	{417., 446.}	56.8504	0.195308	newly 49 weeks cumul	323.	551.71	{528., 575.}	70.808	0.232305
