# Supplementary material for: A Likelihood Approach for Real-Time Calibration of Stochastic Compartmental Epidemic Models
Source: PLoS Comput Biol. 2017 Jan 17;13(1):e1005257. doi: 10.1371/journal.pcbi.1005257 (PMC5240920; doi:10.1371/journal.pcbi.1005257)
Supplement: S1 File — (TAR.GZ) [file pcbi.1005257.s014.tar.gz › HSPH_Online-SI-Revision/MSS10/n10-extreme/n10-extreme_NEW_table4.pdf]

1	R0	{2.68692, 12.5884, 1.}	{2.53075, 11.6597, 0.}	{{2.53075, 11.6597, 0.}, {2.53075, 11.6597, 0.}}	{5.81226, 7.37731, 100.}	inf	669.	607.736	{592.692, 621.162}	9.1575	0.0417954	newly	306.	296.87	{280., 313.}	4.04902	0.0181418	Reff	0.305503	0.30325	{0.298915, 0.306976}	0.999252	ar	9089.	9027.77	{8999., 9055.}	0.673671
2	R0	{1.79802, 12.5412, 1.}	{1.67732, 9.44073, 0.}	{{1.67732, 9.44073, 0.}, {1.67732, 9.44073, 0.}}	{6.713, 24.7223, 100.}	inf	841.	488.123	{460.652, 513.578}	41.9593	0.236595	newly	397.	320.04	{297., 341.}	19.3854	0.0943042	Reff	0.656637	0.671746	{0.667416, 0.676319}	2.30093	ar	7412.	6743.56	{6640., 6842.}	9.01835
3	R0	{2.6174, 10.1105, 0.1}	{2.55445, 9.40056, 0.86}	{{2.52681, 9.219, 0.}, {2.70795, 10.355, 1.}}	{3.54819, 8.14438, 788.}	inf	459.	372.787	{338.426, 488.022}	21.7048	0.106375	newly	256.	212.7	{191., 260.}	18.2578	0.0886388	Reff	0.268022	0.283231	{0.272332, 0.289042}	6.10537	ar	9109.	9024.16	{8972., 9155.}	1.16149
4	R0	{2.27532, 14.3149, 0.1}	{2.0968, 12.1899, 1.}	{{2.0968, 12.1899, 1.}, {2.0968, 12.1899, 1.}}	{7.84572, 14.8445, 900.}	inf	750.	857.717	{838.37, 877.789}	14.3623	0.058221	newly	320.	416.12	{396., 433.}	30.0375	0.11383	Reff	0.473267	0.413549	{0.409174, 0.417498}	12.6181	ar	8478.	8509.11	{8468., 8549.}	0.450696
5	R0	{2.96649, 18.0674, 2.}	{2.94865, 17.6303, 2.}	{{2.8833, 17.1847, 2.}, {2.96398, 17.7348, 2.}}	{0.601367, 2.41932, 0.}	inf	1193.	1100.	{990.035, 1136.37}	7.79552	0.0358271	newly	386.	376.37	{348., 400.}	4.47409	0.0199444	Reff	0.251262	0.277001	{0.267776, 0.302605}	10.244	ar	9476.	9380.68	{9278., 9427.}	1.00591
6	R0	{2.94778, 16.0002, 0.1}	{2.688, 14.8279, 2.}	{{2.688, 14.8279, 2.}, {2.688, 14.8279, 2.}}	{8.81277, 7.32652, 1900.}	inf	1090.	910.73	{895.001, 929.289}	16.4468	0.0780907	newly	362.	366.55	{347., 387.}	3.5221	0.0151227	Reff	0.295957	0.318063	{0.312393, 0.322236}	7.46921	ar	9321.	9151.12	{9122., 9182.}	1.82255
7	R0	{2.17562, 11.7184, 1.}	{1.94077, 8.67109, 0.68}	{{1.93419, 8.56054, 0.}, {1.95355, 8.88568, 2.}}	{10.7945, 26.0045, 100.}	inf	793.	477.562	{449.803, 507.613}	39.7777	0.22072	newly	406.	315.49	{286., 343.}	22.2931	0.110615	Reff	0.496041	0.503722	{0.496384, 0.510997}	1.62694	ar	8345.	7822.76	{7765., 7887.}	6.25812
8	R0	{2.7904, 17.8966, 2.}	{2.47514, 14.5291, 1.}	{{2.47514, 14.5291, 1.}, {2.47514, 14.5291, 1.}}	{11.2981, 18.8163, 50.}	inf	1403.	1141.86	{1119.84, 1162.14}	18.613	0.0894942	newly	475.	470.78	{445., 497.}	3.38947	0.0148558	Reff	0.341266	0.367345	{0.362137, 0.372634}	7.642	ar	9285.	9010.3	{8969., 9049.}	2.95854
9	R0	{2.51574, 16.376, 1.}	{2.63215, 17.0609, 2.}	{{2.53741, 16.1789, 2.}, {2.76849, 18.33, 2.}}	{4.62727, 5.60199, 100.}	inf	1116.	1205.49	{1051.71, 1415.42}	13.2086	0.0525313	newly	427.	432.37	{390., 483.}	7.59016	0.0324585	Reff	0.397235	0.390173	{0.353899, 0.416523}	6.49392	ar	8937.	9047.66	{8871., 9254.}	1.61911
10	R0	{2.56405, 18.1787, 2.}	{2.46976, 17.5057, 3.}	{{2.46976, 17.5057, 3.}, {2.46976, 17.5057, 3.}}	{3.67749, 3.70228, 50.}	inf	1437.	1132.04	{1106.34, 1163.44}	21.222	0.103666	newly	467.	407.46	{385., 427.}	12.7495	0.0596345	Reff	0.441786	0.500857	{0.493033, 0.506772}	13.371	ar	9005.	8730.13	{8682., 8773.}	3.05242
11	R0	{2.475, 17.1709, 1.}	{1.88607, 11.9453, 0.}	{{1.88607, 11.9453, 0.}, {1.88607, 11.9453, 0.}}	{23.7952, 30.4326, 100.}	inf	1270.	659.714	{636.233, 680.508}	48.054	0.284604	newly	447.	327.76	{308., 348.}	26.6756	0.135204	Reff	0.449708	0.457803	{0.453774, 0.461976}	1.80019	ar	8870.	8034.17	{7972., 8086.}	9.42311
12	R0	{2.14974, 11.8619, 0.1}	{2.02235, 10.6804, 0.}	{{2.02235, 10.6804, 0.}, {2.02235, 10.6804, 0.}}	{5.92562, 9.96043, 100.}	inf	994.	759.596	{738.114, 782.022}	23.5819	0.116928	newly	494.	422.28	{399., 443.}	14.5182	0.0684759	Reff	0.487776	0.506277	{0.501594, 0.510486}	3.79295	ar	8414.	8105.46	{8041., 8157.}	3.66698
13	R0	{2.20385, 14.4767, 0.1}	{2.46477, 18.9032, 1.}	{{2.46477, 18.9032, 1.}, {2.46477, 18.9032, 1.}}	{11.8393, 30.5765, 900.}	inf	760.	918.276	{896.374, 938.633}	20.8258	0.0820968	newly	319.	308.97	{286., 331.}	5.15674	0.0230338	Reff	0.478235	0.495844	{0.490788, 0.500996}	3.68197	ar	8347.	8620.3	{8563., 8675.}	3.27423
14	R0	{2.35671, 13.8357, 1.}	{2.38049, 15.9578, 4.}	{{2.28604, 14.5901, 4.}, {2.41197, 16.4137, 4.}}	{2.50817, 15.3378, 300.}	inf	848.	929.431	{869.118, 964.521}	9.61439	0.0395519	newly	361.	355.57	{332., 376.}	4.01385	0.0177561	Reff	0.411717	0.396312	{0.391615, 0.401769}	3.74166	ar	8719.	8810.13	{8710., 8875.}	1.11263
15	R0	{1.91368, 10.1082, 1.}	{1.77826, 9.06807, 1.}	{{1.77826, 9.06807, 1.}, {1.77826, 9.06807, 1.}}	{7.07659, 10.29, 0.}	inf	436.	279.442	{257.348, 301.309}	35.9077	0.194082	newly	261.	182.47	{164., 201.}	30.0881	0.156892	Reff	0.581759	0.56843	{0.564541, 0.572352}	2.29111	ar	7486.	7134.43	{7069., 7186.}	4.69637
16	R0	{2.48727, 14.882, 0.1}	{2.29107, 12.1545, 2.}	{{2.2885, 12.1239, 2.}, {2.2885, 12.1239, 2.}}	{7.9352, 18.3684, 1900.}	inf	1044.	811.664	{786.882, 828.62}	22.4144	0.110345	newly	425.	393.73	{371., 420.}	7.70118	0.0351486	Reff	0.402192	0.423615	{0.418916, 0.428856}	5.38525	ar	8894.	8619.29	{8579., 8649.}	3.10715
17	R0	{2.46795, 13.1915, 0.1}	{2.38259, 13.1011, 3.9}	{{2.15962, 10.7214, 4.}, {2.42221, 13.5694, 4.}}	{3.45883, 5.67009, 3800.}	inf	950.	967.717	{567.299, 1051.54}	12.7695	0.0612522	newly	457.	435.86	{337., 476.}	6.83589	0.0331255	Reff	0.428189	0.407495	{0.395518, 0.445401}	6.25661	ar	8796.	8783.87	{8286., 8898.}	1.50011
18	R0	{2.96603, 16.7543, 0.1}	{2.5684, 13.7324, 1.}	{{2.5684, 13.7324, 1.}, {2.5684, 13.7324, 1.}}	{13.4061, 18.0364, 900.}	inf	1079.	834.324	{822.193, 847.108}	22.6762	0.111723	newly	402.	357.44	{336., 375.}	11.0846	0.0513809	Reff	0.284146	0.308895	{0.305594, 0.311978}	8.71022	ar	9394.	9104.21	{9070., 9136.}	3.08484
19	R0	{2.25216, 9.90465, 0.1}	{2.22287, 9.34395, 0.}	{{2.22376, 9.35432, 0.}, {2.22376, 9.35432, 0.}}	{1.30058, 5.66095, 100.}	inf	643.	546.124	{522.544, 566.625}	15.0663	0.0711331	newly	379.	329.73	{306., 350.}	13.	0.0610155	Reff	0.403812	0.420089	{0.41566, 0.42458}	4.03069	ar	8631.	8445.31	{8404., 8497.}	2.15143
20	R0	{2.70837, 18.1923, 0.1}	{2.39458, 15.0578, 1.98}	{{2.40085, 15.146, 2.}, {2.40085, 15.146, 2.}}	{11.5858, 17.23, 1880.}	inf	1399.	994.376	{975.805, 1021.68}	28.9224	0.148728	newly	468.	400.91	{377., 422.}	14.3355	0.0675883	Reff	0.365088	0.419553	{0.413867, 0.424796}	14.9182	ar	9183.	8780.73	{8733., 8830.}	4.38059
21	R0	{2.30288, 15.5877, 1.}	{2.11292, 12.9719, 0.}	{{2.11292, 12.9719, 0.}, {2.11292, 12.9719, 0.}}	{8.24883, 16.7811, 100.}	inf	1029.	491.094	{474.004, 509.982}	52.2746	0.321423	newly	405.	228.98	{210., 243.}	43.4617	0.248491	Reff	0.441923	0.519124	{0.515072, 0.522557}	17.4695	ar	8674.	7985.17	{7922., 8042.}	7.94132
22	R0	{2.82208, 10.7544, 0.1}	{2.70795, 10.355, 0.}	{{2.70795, 10.355, 0.}, {2.70795, 10.355, 0.}}	{4.04401, 3.71421, 100.}	inf	586.	447.718	{431.469, 461.513}	23.5976	0.11703	newly	278.	238.44	{222., 253.}	14.2302	0.0673257	Reff	0.244392	0.271955	{0.267397, 0.275916}	11.2781	ar	9333.	9145.53	{9112., 9173.}	2.00868
23	R0	{1.69923, 10.1366, 1.}	{2.18067, 16.87, 4.}	{{2.18067, 16.87, 4.}, {2.18067, 16.87, 4.}}	{28.333, 66.4271, 300.}	inf	558.	1118.48	{1091.45, 1142.67}	100.444	0.301919	newly	330.	431.58	{406., 453.}	30.7818	0.116157	Reff	0.599318	0.646901	{0.641514, 0.652692}	7.93951	ar	7055.	8189.89	{8115., 8264.}	16.0863
24	R0	{2.64944, 19.054, 1.}	{2.76795, 19.6041, 1.}	{{2.76795, 19.6041, 1.}, {2.76795, 19.6041, 1.}}	{4.47298, 2.88729, 0.}	inf	1099.	1271.07	{1251.5, 1286.31}	15.6573	0.0631483	newly	382.	402.05	{382., 422.}	5.58377	0.0233692	Reff	0.35317	0.321338	{0.316389, 0.326046}	9.0132	ar	9147.	9276.69	{9246., 9316.}	1.41784
25	R0	{2.53191, 10.9214, 0.1}	{2.2983, 8.81055, 0.}	{{2.2983, 8.81055, 0.}, {2.2983, 8.81055, 0.}}	{9.22682, 19.3276, 100.}	inf	571.	304.257	{286.558, 321.298}	46.715	0.273777	newly	309.	186.87	{169., 201.}	39.5243	0.219326	Reff	0.310159	0.342847	{0.338926, 0.34691}	10.539	ar	9009.	8655.27	{8615., 8692.}	3.92641
26	R0	{1.89155, 6.76944, 0.1}	{1.78724, 6.63253, 2.}	{{1.78724, 6.63253, 2.}, {1.78724, 6.63253, 2.}}	{5.51464, 2.02251, 1900.}	inf	234.	188.88	{172.53, 205.351}	19.282	0.0941588	newly	188.	154.61	{135., 172.}	17.8777	0.0873795	Reff	0.540416	0.518678	{0.515434, 0.521587}	4.02246	ar	7348.	7289.	{7236., 7329.}	0.824442
27	R0	{2.63149, 15.6979, 0.1}	{2.39501, 13.4525, 2.}	{{2.2885, 12.1239, 2.}, {2.5458, 15.1849, 2.}}	{10.7959, 17.5652, 1900.}	inf	1228.	912.676	{826.875, 1151.51}	29.5034	0.150689	newly	463.	400.34	{361., 445.}	14.6652	0.0695975	Reff	0.367882	0.407216	{0.373618, 0.424112}	12.6956	ar	9104.	8762.51	{8649., 9024.}	4.23034
28	R0	{2.85141, 12.6992, 1.}	{2.78012, 12.0284, 1.91}	{{2.79531, 12.1249, 2.}, {2.79531, 12.1249, 2.}}	{2.5, 5.28212, 91.}	inf	776.	714.834	{705.235, 746.269}	7.8822	0.0372593	newly	347.	336.48	{315., 360.}	4.75504	0.0217844	Reff	0.251494	0.261945	{0.254854, 0.266259}	4.15526	ar	9317.	9269.94	{9245., 9315.}	0.521412
29	R0	{2.54244, 12.9085, 0.1}	{2.32891, 10.9956, 0.}	{{2.32891, 10.9956, 0.}, {2.32891, 10.9956, 0.}}	{8.39874, 14.8192, 100.}	inf	779.	657.397	{638.831, 675.146}	15.6101	0.0738158	newly	343.	338.87	{321., 357.}	3.4723	0.0152821	Reff	0.329754	0.330379	{0.326074, 0.334657}	0.819794	ar	9034.	8861.3	{8824., 8891.}	1.91167
30	R0	{2.10982, 11.7636, 1.}	{1.96658, 10.2409, 1.}	{{1.96658, 10.2409, 1.}, {1.96658, 10.2409, 1.}}	{6.78903, 12.9444, 0.}	inf	703.	518.463	{495.181, 540.701}	26.2499	0.13244	newly	366.	296.14	{273., 314.}	19.0874	0.0926119	Reff	0.486735	0.489982	{0.485582, 0.494495}	0.76511	ar	8224.	7924.58	{7873., 7976.}	3.64081
31	R0	{2.76312, 15.5396, 0.1}	{2.62448, 14.9972, 2.91}	{{2.62879, 15.1183, 3.}, {2.62879, 15.1183, 3.}}	{5.01741, 3.49013, 2810.}	inf	1113.	1079.79	{1052.94, 1101.17}	3.03599	0.0135482	newly	439.	428.23	{405., 450.}	3.71982	0.0165631	Reff	0.324667	0.317058	{0.311547, 0.323915}	2.80556	ar	9220.	9178.35	{9128., 9219.}	0.477115
32	R0	{2.21727, 15.5439, 0.1}	{2.08951, 15.9744, 0.}	{{2.08951, 15.9744, 0.}, {2.08951, 15.9744, 0.}}	{5.7622, 2.76988, 100.}	inf	936.	648.306	{628.334, 666.593}	30.7366	0.159606	newly	399.	254.62	{234., 275.}	36.1855	0.195832	Reff	0.506203	0.537148	{0.532871, 0.541245}	6.11325	ar	8359.	8016.67	{7956., 8069.}	4.09535
33	R0	{2.72912, 10.8159, 0.1}	{2.5684, 9.75224, 1.}	{{2.5684, 9.75224, 1.}, {2.5684, 9.75224, 1.}}	{5.88896, 9.83426, 900.}	inf	395.	279.231	{268.052, 290.563}	29.3086	0.150858	newly	210.	154.96	{142., 171.}	26.2095	0.13307	Reff	0.252989	0.267825	{0.264754, 0.270626}	5.86414	ar	9196.	9053.9	{9030., 9077.}	1.54524
34	R0	{2.20928, 10.1308, 1.}	{2.10405, 8.58066, 3.}	{{2.10405, 8.58066, 3.}, {2.10405, 8.58066, 3.}}	{4.76324, 15.3012, 200.}	inf	446.	271.098	{250.418, 290.73}	39.2157	0.216861	newly	238.	174.73	{158., 193.}	26.584	0.135481	Reff	0.406066	0.423524	{0.419348, 0.427608}	4.2994	ar	8388.	8167.34	{8132., 8213.}	2.63066
35	R0	{2.10103, 15.2673, 2.}	{2.03831, 13.1693, 3.}	{{2.03831, 13.1693, 3.}, {2.03831, 13.1693, 3.}}	{2.98541, 13.7421, 50.}	inf	986.	790.292	{769., 811.219}	19.8487	0.0961995	newly	382.	369.13	{350., 386.}	4.13351	0.0185196	Reff	0.508869	0.533569	{0.529204, 0.53784}	4.85378	ar	8264.	8059.67	{7999., 8134.}	2.47253
36	R0	{2.74914, 19.2775, 1.}	{2.11292, 12.9719, 0.}	{{2.11292, 12.9719, 0.}, {2.11292, 12.9719, 0.}}	{23.1425, 32.7096, 100.}	inf	1441.	528.936	{508.217, 546.847}	63.2938	0.435421	newly	436.	247.14	{229., 265.}	43.3165	0.247362	Reff	0.366735	0.474575	{0.470783, 0.478853}	29.4054	ar	9301.	8148.51	{8094., 8195.}	12.391
37	R0	{2.78161, 16.0136, 0.1}	{2.7499, 15.3124, 0.}	{{2.7499, 15.3124, 0.}, {2.7499, 15.3124, 0.}}	{1.14008, 4.3787, 100.}	inf	1033.	873.354	{851.475, 890.387}	15.4546	0.072972	newly	388.	342.3	{322., 363.}	11.7784	0.0549213	Reff	0.3146	0.354914	{0.35022, 0.360872}	12.8145	ar	9240.	9086.07	{9047., 9126.}	1.66591
38	R0	{2.4454, 8.33501, 0.1}	{2.41784, 8.02695, 4.}	{{2.41784, 8.02695, 4.}, {2.41784, 8.02695, 4.}}	{1.12696, 3.696, 3900.}	inf	243.	215.861	{202.338, 229.303}	11.1685	0.0519583	newly	147.	141.67	{132., 154.}	5.77551	0.0260015	Reff	0.306653	0.309759	{0.306434, 0.312801}	1.08079	ar	8853.	8812.66	{8785., 8841.}	0.460409
39	R0	{2.9154, 12.3861, 0.1}	{2.80569, 11.6897, 1.}	{{2.80569, 11.6897, 1.}, {2.80569, 11.6897, 1.}}	{3.76316, 5.62267, 900.}	inf	596.	472.846	{458.713, 487.462}	20.6635	0.100645	newly	284.	227.48	{207., 244.}	19.9014	0.0971328	Reff	0.225069	0.251152	{0.246868, 0.25504}	11.5892	ar	9371.	9248.46	{9223., 9277.}	1.30765
40	R0	{2.40923, 15.1963, 0.1}	{2.13049, 12.3671, 2.9}	{{2.04871, 11.6322, 2.}, {2.04871, 11.6322, 3.}}	{15.3661, 23.5991, 2800.}	inf	1025.	668.582	{560.378, 614.429}	43.4079	0.238417	newly	421.	313.63	{271., 348.}	28.0831	0.143966	Reff	0.424988	0.445219	{0.387185, 0.456519}	6.93398	ar	8735.	8301.42	{8149., 8265.}	6.19989
41	R0	{2.63137, 15.238, 1.}	{2.86501, 18.4249, 1.}	{{2.86501, 18.4249, 1.}, {2.86501, 18.4249, 1.}}	{8.87891, 20.9144, 0.}	inf	1020.	1361.91	{1346.45, 1378.71}	33.5209	0.12553	newly	409.	448.05	{422., 469.}	9.56235	0.0393367	Reff	0.336026	0.267903	{0.262929, 0.272298}	20.2731	ar	9150.	9429.5	{9403., 9454.}	3.05464
42	R0	{2.48769, 19.4593, 0.1}	{2.01705, 13.609, 0.}	{{2.01705, 13.609, 0.}, {2.01705, 13.609, 0.}}	{18.9188, 30.0645, 100.}	inf	1184.	780.187	{763.085, 801.28}	34.1058	0.181228	newly	394.	345.99	{326., 367.}	12.1853	0.0568617	Reff	0.413205	0.416483	{0.412228, 0.419893}	0.892399	ar	8932.	8389.79	{8343., 8431.}	6.07042
43	R0	{2.61199, 10.214, 0.1}	{2.73023, 10.6213, 0.06}	{{2.74056, 10.6768, 0.}, {2.74056, 10.6768, 0.}}	{4.72702, 4.53002, 148.}	inf	551.	659.686	{646.667, 687.435}	21.3625	0.0844508	newly	296.	345.37	{317., 371.}	17.7601	0.0706329	Reff	0.299073	0.282747	{0.276226, 0.286724}	6.03314	ar	9061.	9183.42	{9152., 9229.}	1.48505
44	R0	{2.50994, 18.3382, 0.1}	{2.01705, 13.609, 0.}	{{2.01705, 13.609, 0.}, {2.01705, 13.609, 0.}}	{19.6376, 25.7891, 100.}	inf	1257.	695.91	{673.092, 716.948}	44.6372	0.256906	newly	414.	314.24	{292., 338.}	24.0966	0.120356	Reff	0.47714	0.496615	{0.492343, 0.501174}	4.08178	ar	8827.	8079.08	{8023., 8137.}	8.47309
45	R0	{2.44283, 16.8338, 0.1}	{2.29887, 14.662, 2.}	{{2.29887, 14.662, 2.}, {2.29887, 14.662, 2.}}	{5.89311, 12.9013, 1900.}	inf	1098.	949.131	{927.372, 972.741}	13.5582	0.0633429	newly	418.	393.81	{375., 414.}	6.08852	0.0275317	Reff	0.427251	0.436296	{0.430817, 0.441202}	2.13126	ar	8878.	8659.31	{8608., 8709.}	2.46328
46	R0	{2.51815, 18.1462, 5.}	{2.11292, 12.9719, 0.}	{{2.11292, 12.9719, 0.}, {2.11292, 12.9719, 0.}}	{16.0924, 28.5144, 100.}	inf	1305.	953.774	{928.871, 978.712}	26.9139	0.136257	newly	464.	442.24	{418., 466.}	5.40086	0.0242821	Reff	0.458051	0.458551	{0.452702, 0.463512}	0.720995	ar	8978.	8436.64	{8380., 8486.}	6.02985
47	R0	{2.88885, 16.8987, 0.1}	{2.96386, 18.8386, 0.}	{{2.96978, 18.8857, 0.}, {2.96978, 18.8857, 0.}}	{2.61418, 11.4797, 100.}	inf	1290.	1351.28	{1332.91, 1374.93}	5.02308	0.0212654	newly	468.	441.12	{417., 463.}	5.88034	0.0266216	Reff	0.329618	0.319962	{0.31326, 0.324845}	3.21944	ar	9337.	9365.79	{9339., 9398.}	0.386741
48	R0	{2.58709, 13.8786, 0.1}	{2.45575, 12.339, 1.01}	{{2.4534, 12.3139, 1.}, {2.4534, 12.3139, 1.}}	{5.15481, 11.2302, 910.}	inf	1037.	821.683	{798.169, 838.366}	20.8957	0.101934	newly	435.	389.47	{367., 406.}	10.5172	0.0486341	Reff	0.330113	0.366163	{0.361711, 0.371596}	10.9544	ar	9146.	8889.96	{8849., 8932.}	2.79948
49	R0	{2.42959, 10.7132, 0.1}	{2.3598, 10.089, 2.}	{{2.33405, 9.83948, 2.}, {2.36298, 10.1198, 2.}}	{2.87245, 5.82647, 1900.}	inf	676.	617.006	{597.061, 640.53}	8.72697	0.0398099	newly	359.	345.	{326., 366.}	4.76323	0.0213948	Reff	0.358122	0.361752	{0.356555, 0.366058}	1.19636	ar	8863.	8766.49	{8733., 8801.}	1.08891
50	R0	{2.60513, 13.2796, 0.1}	{2.7499, 15.3124, 0.}	{{2.7499, 15.3124, 0.}, {2.7499, 15.3124, 0.}}	{5.55701, 15.3078, 100.}	inf	1051.	1025.7	{1002.97, 1045.03}	2.45127	0.0108225	newly	465.	406.94	{387., 426.}	12.486	0.0582695	Reff	0.354037	0.380668	{0.375284, 0.3867}	7.52214	ar	9095.	9078.48	{9041., 9104.}	0.251787
