# Supplementary material for: A Likelihood Approach for Real-Time Calibration of Stochastic Compartmental Epidemic Models
Source: PLoS Comput Biol. 2017 Jan 17;13(1):e1005257. doi: 10.1371/journal.pcbi.1005257 (PMC5240920; doi:10.1371/journal.pcbi.1005257)
Supplement: S1 File — (TAR.GZ) [file pcbi.1005257.s014.tar.gz › HSPH_Online-SI-Revision/MSS10/n10-extreme/n10-extreme_NEW2_table-4.pdf]

1	newly 3 weeks spec	1350.	1214.17	{1034., 1368.}	11.2037	0.05411	newly 3 weeks cumul	2770.	2658.12	{2254., 3044.}	9.47076	0.0433666	newly 5 weeks spec	1264.	1022.1	{785., 1215.}	19.1566	0.0997894	newly 5 weeks cumul	5455.	4922.91	{4250., 5396.}	10.0145	0.0480365	newly 62 weeks cumul	8628.	6978.57	{5509., 8494.}	19.1496	0.0979525
2	newly 3 weeks spec	604.	610.7	{372., 734.}	19.5166	0.0917039	newly 3 weeks cumul	1548.	1597.42	{1159., 1835.}	15.7041	0.0713478	newly 5 weeks spec	678.	629.15	{301., 796.}	23.2552	0.126705	newly 5 weeks cumul	2970.	2867.09	{1910., 3383.}	16.6684	0.0818905	newly 58 weeks cumul	6502.	5726.87	{2711., 7191.}	23.8221	0.13755
3	newly 3 weeks spec	1586.	1928.77	{1796., 2035.}	21.6122	0.0843956	newly 3 weeks cumul	3187.	3945.29	{3584., 4258.}	23.8422	0.091764	newly 5 weeks spec	1325.	1258.16	{1107., 1368.}	6.57962	0.0306466	newly 5 weeks cumul	6258.	6957.35	{6673., 7189.}	11.2095	0.0459496	newly 64 weeks cumul	8818.	8921.05	{8136., 9128.}	3.59163	0.015763
4	newly 3 weeks spec	888.	889.69	{829., 985.}	6.25113	0.0289571	newly 3 weeks cumul	2367.	2362.01	{2212., 2567.}	5.36586	0.0240028	newly 5 weeks spec	862.	768.22	{710., 835.}	11.0325	0.0574934	newly 5 weeks cumul	4124.	3993.41	{3807., 4311.}	5.266	0.025037	newly 58 weeks cumul	7204.	6642.96	{6176., 6961.}	7.7879	0.0387526
5	newly 3 weeks spec	1149.	1129.9	{1075., 1198.}	4.34117	0.0197464	newly 3 weeks cumul	2806.	2836.45	{2697., 2997.}	3.81433	0.0167101	newly 5 weeks spec	1043.	959.43	{893., 1038.}	8.24832	0.0421069	newly 5 weeks cumul	5000.	4903.01	{4797., 5099.}	3.2414	0.015153	newly 61 weeks cumul	8544.	7911.57	{6884., 8470.}	7.40204	0.03749
6	newly 3 weeks spec	1137.	1024.93	{798., 1227.}	13.343	0.0652155	newly 3 weeks cumul	2504.	2460.27	{1887., 3067.}	13.9069	0.0633335	newly 5 weeks spec	1175.	749.46	{587., 1020.}	36.2162	0.204104	newly 5 weeks cumul	4971.	4168.12	{3488., 4813.}	16.2836	0.0802855	newly 62 weeks cumul	8740.	5489.02	{4786., 6996.}	37.1966	0.207535
7	newly 3 weeks spec	962.	1103.08	{611., 1305.}	29.5198	0.130435	newly 3 weeks cumul	2111.	2490.22	{1708., 2862.}	29.2411	0.12542	newly 5 weeks spec	1027.	1048.76	{372., 1261.}	27.8812	0.159654	newly 5 weeks cumul	4213.	4694.27	{2687., 5417.}	26.4398	0.11932	newly 63 weeks cumul	7880.	7722.85	{3311., 8954.}	21.5138	0.115339
8	newly 3 weeks spec	963.	1010.86	{932., 1100.}	10.0415	0.0446432	newly 3 weeks cumul	2334.	2417.53	{2241., 2576.}	6.57541	0.0283075	newly 5 weeks spec	978.	960.45	{605., 1083.}	11.4591	0.0620276	newly 5 weeks cumul	4380.	4428.26	{3975., 4752.}	7.08402	0.0326539	newly 60 weeks cumul	8557.	7688.65	{4908., 8400.}	10.2292	0.05748
9	newly 3 weeks spec	987.	1000.16	{938., 1063.}	4.85917	0.0221428	newly 3 weeks cumul	2373.	2467.28	{2316., 2621.}	6.16772	0.0269715	newly 5 weeks spec	932.	961.47	{925., 1006.}	4.647	0.0208888	newly 5 weeks cumul	4333.	4466.4	{4269., 4661.}	4.6859	0.0209099	newly 60 weeks cumul	8041.	8290.09	{8226., 8445.}	4.64009	0.0213036
10	newly 3 weeks spec	784.	571.52	{314., 832.}	30.0434	0.181866	newly 3 weeks cumul	1916.	1563.	{882., 2093.}	23.381	0.135128	newly 5 weeks spec	979.	472.74	{226., 897.}	51.7753	0.373512	newly 5 weeks cumul	3879.	2573.6	{1551., 3769.}	34.0608	0.207678	newly 60 weeks cumul	8180.	4104.28	{2389., 7735.}	49.8254	0.343596
11	newly 3 weeks spec	908.	447.01	{330., 580.}	50.7698	0.326498	newly 3 weeks cumul	2169.	1312.19	{919., 1718.}	39.5025	0.237619	newly 5 weeks spec	841.	322.86	{221., 424.}	61.61	0.431788	newly 5 weeks cumul	3950.	2024.6	{1521., 2496.}	48.7443	0.304174	newly 55 weeks cumul	7949.	2857.26	{2367., 3268.}	64.0551	0.451125
12	newly 3 weeks spec	811.	436.42	{120., 746.}	46.1874	0.380788	newly 3 weeks cumul	1803.	1079.59	{398., 1693.}	40.3722	0.290432	newly 5 weeks spec	1021.	431.79	{108., 910.}	57.7659	0.549059	newly 5 weeks cumul	3891.	1969.99	{643., 3458.}	49.3706	0.376026	newly 60 weeks cumul	7914.	3557.21	{1404., 7741.}	55.8723	0.448434
13	newly 3 weeks spec	841.	440.27	{295., 562.}	47.7515	0.301011	newly 3 weeks cumul	2317.	1408.01	{915., 1895.}	39.2607	0.236574	newly 5 weeks spec	848.	265.85	{151., 340.}	68.6498	0.535865	newly 5 weeks cumul	4080.	2037.88	{1354., 2548.}	50.052	0.319089	newly 56 weeks cumul	7098.	2686.	{1888., 3080.}	62.7937	0.445811
14	newly 3 weeks spec	971.	974.45	{648., 1178.}	17.7291	0.0822304	newly 3 weeks cumul	2497.	2577.1	{1771., 2953.}	14.9884	0.066847	newly 5 weeks spec	946.	728.33	{414., 1029.}	28.982	0.175187	newly 5 weeks cumul	4507.	4199.63	{2781., 5047.}	16.9716	0.0833416	newly 61 weeks cumul	7771.	6198.35	{3135., 8447.}	27.8799	0.158734
15	newly 3 weeks spec	832.	863.95	{815., 910.}	4.83053	0.0203155	newly 3 weeks cumul	2173.	2221.58	{2116., 2311.}	3.29498	0.0139959	newly 5 weeks spec	838.	846.22	{794., 887.}	3.69928	0.0160003	newly 5 weeks cumul	3908.	3962.88	{3840., 4081.}	2.36592	0.0101368	newly 58 weeks cumul	6416.	7593.7	{7553., 7743.}	18.7609	0.0745705
16	newly 3 weeks spec	1062.	1109.98	{1006., 1227.}	7.3484	0.0304612	newly 3 weeks cumul	2353.	2449.21	{2210., 2743.}	7.19465	0.0297844	newly 5 weeks spec	1082.	1151.54	{1101., 1201.}	6.48429	0.0270765	newly 5 weeks cumul	4527.	4821.68	{4472., 5177.}	6.99845	0.0288435	newly 61 weeks cumul	8363.	8612.7	{8307., 8858.}	3.40787	0.01448
17	newly 3 weeks spec	1033.	1274.58	{1193., 1375.}	23.4327	0.0905657	newly 3 weeks cumul	2323.	2788.33	{2544., 3095.}	20.1907	0.078724	newly 5 weeks spec	1116.	1160.85	{981., 1252.}	9.95789	0.0441978	newly 5 weeks cumul	4627.	5279.67	{5047., 5580.}	14.5215	0.0586399	newly 63 weeks cumul	8302.	8389.77	{6613., 8914.}	8.44748	0.0384473
18	newly 3 weeks spec	1151.	1084.36	{978., 1191.}	7.45265	0.0340656	newly 3 weeks cumul	2756.	2563.2	{2334., 2834.}	8.5225	0.0391799	newly 5 weeks spec	1119.	1018.97	{952., 1080.}	8.94996	0.041245	newly 5 weeks cumul	5058.	4711.83	{4449., 5087.}	7.57829	0.0344832	newly 61 weeks cumul	8663.	7999.46	{7039., 8680.}	7.78552	0.0365885
19	newly 3 weeks spec	1098.	1353.76	{1106., 1579.}	25.5501	0.0978589	newly 3 weeks cumul	2182.	2598.79	{2080., 3013.}	22.61	0.0880888	newly 5 weeks spec	1350.	1324.02	{1156., 1447.}	6.27704	0.0286946	newly 5 weeks cumul	4959.	5458.67	{4758., 5986.}	13.0819	0.0539203	newly 63 weeks cumul	8373.	8000.36	{6852., 8837.}	6.29285	0.0296109
20	newly 3 weeks spec	902.	1046.43	{984., 1109.}	17.0521	0.069031	newly 3 weeks cumul	2235.	2455.86	{2301., 2614.}	11.081	0.0465154	newly 5 weeks spec	986.	1044.85	{991., 1096.}	6.83874	0.0288318	newly 5 weeks cumul	4273.	4611.79	{4434., 4802.}	8.88111	0.0375925	newly 60 weeks cumul	8476.	8312.36	{7928., 8648.}	3.12765	0.0146514
21	newly 3 weeks spec	966.	883.	{829., 942.}	8.80952	0.0404935	newly 3 weeks cumul	2273.	2218.54	{2126., 2319.}	3.64452	0.0161371	newly 5 weeks spec	836.	860.4	{825., 893.}	3.58852	0.0152162	newly 5 weeks cumul	4121.	3997.31	{3867., 4150.}	3.48435	0.0154806	newly 57 weeks cumul	7668.	7237.25	{7131., 7323.}	5.7174	0.0255829
22	newly 3 weeks spec	1428.	1259.71	{1042., 1517.}	14.9629	0.074415	newly 3 weeks cumul	2568.	2311.67	{1897., 2772.}	14.4155	0.0701303	newly 5 weeks spec	1622.	1413.22	{1253., 1542.}	12.8718	0.0641668	newly 5 weeks cumul	6049.	5267.34	{4609., 5963.}	13.2455	0.0653148	newly 64 weeks cumul	9138.	8293.41	{6935., 9222.}	9.62629	0.0476443
23	newly 3 weeks spec	671.	411.58	{272., 534.}	38.6617	0.229633	newly 3 weeks cumul	1760.	1275.26	{728., 1772.}	29.1318	0.16783	newly 5 weeks spec	667.	273.13	{168., 366.}	59.051	0.405206	newly 5 weeks cumul	3172.	1898.58	{1336., 2412.}	40.1456	0.236648	newly 59 weeks cumul	5972.	2523.43	{2117., 2983.}	57.7456	0.379636
24	newly 3 weeks spec	922.	942.1	{905., 981.}	3.15835	0.0134557	newly 3 weeks cumul	2515.	2606.39	{2539., 2669.}	3.80398	0.0161436	newly 5 weeks spec	778.	807.73	{767., 844.}	4.991	0.0212132	newly 5 weeks cumul	4256.	4316.64	{4228., 4414.}	1.93609	0.00833546	newly 57 weeks cumul	7579.	7580.31	{7558., 7690.}	1.45257	0.0065851
25	newly 3 weeks spec	1376.	1414.93	{1059., 1696.}	15.3859	0.0664458	newly 3 weeks cumul	2783.	2983.68	{2323., 3642.}	16.345	0.0680904	newly 5 weeks spec	1327.	1038.26	{736., 1206.}	21.7589	0.113346	newly 5 weeks cumul	5644.	5407.11	{4334., 6264.}	11.0633	0.051308	newly 60 weeks cumul	8660.	6891.35	{5559., 7648.}	20.4232	0.102625
26	newly 3 weeks spec	1193.	1046.35	{604., 1307.}	18.8039	0.104567	newly 3 weeks cumul	2728.	2433.31	{1604., 3021.}	16.5891	0.0901318	newly 5 weeks spec	913.	852.42	{326., 1236.}	34.5257	0.180898	newly 5 weeks cumul	4845.	4312.06	{2682., 5436.}	19.7428	0.106057	newly 63 weeks cumul	6836.	6274.09	{3066., 8866.}	31.7348	0.159817
27	newly 3 weeks spec	1034.	1200.38	{1100., 1294.}	16.1489	0.0643065	newly 3 weeks cumul	2307.	2658.36	{2469., 2838.}	15.2302	0.0609591	newly 5 weeks spec	1086.	1159.92	{1109., 1209.}	7.1326	0.0297498	newly 5 weeks cumul	4510.	5103.24	{4862., 5323.}	13.1539	0.053403	newly 61 weeks cumul	8570.	8365.43	{8037., 8833.}	3.67316	0.016366
28	newly 3 weeks spec	1424.	1619.15	{1403., 1794.}	15.0063	0.0596132	newly 3 weeks cumul	2886.	3352.74	{2798., 3791.}	18.3119	0.0713367	newly 5 weeks spec	1362.	1196.1	{987., 1361.}	12.6021	0.0696924	newly 5 weeks cumul	5761.	6120.8	{5715., 6605.}	7.47058	0.0311445	newly 64 weeks cumul	8981.	8107.63	{7458., 8896.}	9.72754	0.046149
29	newly 3 weeks spec	1269.	1118.46	{551., 1529.}	27.015	0.150567	newly 3 weeks cumul	2814.	2687.09	{1490., 3475.}	22.8149	0.115415	newly 5 weeks spec	1154.	796.44	{302., 1140.}	31.2236	0.221511	newly 5 weeks cumul	5251.	4502.41	{2422., 5934.}	24.0867	0.136349	newly 59 weeks cumul	8475.	5961.97	{2983., 7809.}	29.6523	0.187626
30	newly 3 weeks spec	975.	850.21	{475., 998.}	14.3272	0.0844641	newly 3 weeks cumul	2275.	2102.09	{1334., 2377.}	10.7758	0.0609184	newly 5 weeks spec	882.	804.88	{268., 1013.}	21.7438	0.137937	newly 5 weeks cumul	4197.	3777.6	{2051., 4348.}	12.3841	0.0736819	newly 59 weeks cumul	7393.	6704.47	{2680., 8272.}	20.0134	0.120883
31	newly 3 weeks spec	1130.	1075.22	{891., 1264.}	11.2	0.051957	newly 3 weeks cumul	2628.	2814.57	{2329., 3384.}	13.4334	0.0556491	newly 5 weeks spec	1066.	623.33	{463., 839.}	41.9128	0.25154	newly 5 weeks cumul	4862.	4330.41	{3680., 5170.}	13.0315	0.0623848	newly 62 weeks cumul	8496.	5332.51	{4776., 6382.}	37.3601	0.209956
32	newly 3 weeks spec	826.	745.75	{702., 791.}	9.75182	0.0450013	newly 3 weeks cumul	2183.	1930.63	{1862., 2005.}	11.5607	0.0535397	newly 5 weeks spec	784.	770.56	{732., 815.}	3.71429	0.0163879	newly 5 weeks cumul	3849.	3489.47	{3404., 3583.}	9.34087	0.0426786	newly 54 weeks cumul	7242.	7645.96	{7569., 7722.}	5.57802	0.0235604
33	newly 3 weeks spec	1646.	1374.44	{1281., 1447.}	16.4982	0.0788937	newly 3 weeks cumul	3764.	2972.8	{2748., 3163.}	21.0202	0.103181	newly 5 weeks spec	1179.	1233.15	{1168., 1293.}	5.54114	0.0233738	newly 5 weeks cumul	6592.	5633.01	{5394., 5819.}	14.5478	0.0685553	newly 62 weeks cumul	8743.	8628.97	{8076., 8867.}	2.43383	0.0110214
34	newly 3 weeks spec	1346.	940.84	{552., 1341.}	31.7578	0.192878	newly 3 weeks cumul	2950.	2529.67	{1474., 3805.}	27.7502	0.150164	newly 5 weeks spec	1080.	486.77	{186., 803.}	55.6583	0.420252	newly 5 weeks cumul	5397.	3759.91	{2552., 4788.}	31.567	0.183186	newly 64 weeks cumul	7875.	4461.83	{3084., 5596.}	44.2815	0.266999
35	newly 3 weeks spec	787.	930.24	{894., 1010.}	24.6201	0.103509	newly 3 weeks cumul	2046.	2402.64	{2307., 2567.}	22.2053	0.0917762	newly 5 weeks spec	782.	849.95	{817., 942.}	18.2826	0.0903738	newly 5 weeks cumul	3686.	4178.41	{4053., 4464.}	19.837	0.0858298	newly 59 weeks cumul	7161.	7588.78	{6552., 8238.}	16.1679	0.0799272
36	newly 3 weeks spec	900.	876.35	{731., 1009.}	10.5722	0.0503352	newly 3 weeks cumul	2289.	2338.1	{1865., 2785.}	11.8104	0.0511146	newly 5 weeks spec	946.	602.09	{437., 804.}	36.4133	0.214171	newly 5 weeks cumul	4201.	3720.35	{3118., 4210.}	12.0969	0.0598931	newly 57 weeks cumul	8382.	4943.32	{4135., 5956.}	41.0246	0.237274
37	newly 3 weeks spec	1140.	1018.9	{472., 1480.}	32.7965	0.187951	newly 3 weeks cumul	2692.	2560.13	{1176., 3492.}	27.9721	0.152264	newly 5 weeks spec	1097.	693.36	{241., 1074.}	36.9353	0.272332	newly 5 weeks cumul	4963.	4163.96	{2027., 5810.}	28.9313	0.171396	newly 60 weeks cumul	8552.	5495.54	{2637., 7830.}	35.7397	0.23306
38	newly 3 weeks spec	1819.	1370.24	{1001., 1922.}	27.2633	0.150448	newly 3 weeks cumul	3439.	2541.49	{1844., 3653.}	29.4856	0.163804	newly 5 weeks spec	1286.	1337.98	{1045., 1512.}	13.3639	0.0607016	newly 5 weeks cumul	6558.	5450.26	{4554., 6421.}	17.5297	0.0884211	newly 63 weeks cumul	8641.	8074.71	{6673., 9024.}	8.88219	0.0423923
39	newly 3 weeks spec	1549.	1707.99	{1586., 1820.}	11.7398	0.0483374	newly 3 weeks cumul	3391.	3743.71	{3362., 4070.}	11.8688	0.0484022	newly 5 weeks spec	1272.	1166.52	{1090., 1249.}	8.46698	0.0401073	newly 5 weeks cumul	6224.	6477.98	{6166., 6824.}	5.97622	0.0256763	newly 63 weeks cumul	8953.	8489.3	{7689., 8856.}	5.17927	0.0242865
40	newly 3 weeks spec	954.	489.06	{335., 627.}	48.7358	0.304011	newly 3 weeks cumul	2231.	1507.73	{999., 1914.}	33.0224	0.186196	newly 5 weeks spec	887.	303.14	{182., 425.}	65.8241	0.48532	newly 5 weeks cumul	4133.	2211.1	{1652., 2695.}	46.5013	0.282047	newly 60 weeks cumul	7710.	2858.97	{2266., 3467.}	62.9187	0.438177
41	newly 3 weeks spec	1055.	1093.65	{978., 1214.}	11.6502	0.0670798	newly 3 weeks cumul	2675.	2743.73	{2392., 2982.}	10.206	0.0598919	newly 5 weeks spec	964.	935.76	{884., 1022.}	7.39004	0.0465121	newly 5 weeks cumul	4723.	4746.05	{4243., 5103.}	8.50413	0.0510667	newly 59 weeks cumul	8244.	7619.96	{6883., 8200.}	7.59316	0.0449559
42	newly 3 weeks spec	812.	919.17	{884., 962.}	13.7401	0.0559594	newly 3 weeks cumul	2241.	2571.63	{2507., 2645.}	15.0303	0.0608069	newly 5 weeks spec	727.	782.52	{748., 829.}	9.48556	0.0408013	newly 5 weeks cumul	3835.	4230.39	{4153., 4332.}	11.0099	0.0456405	newly 54 weeks cumul	7356.	7485.9	{7496., 7610.}	3.66096	0.0172278
43	newly 3 weeks spec	1345.	1434.45	{1177., 1678.}	12.6342	0.0528178	newly 3 weeks cumul	2361.	2515.72	{1990., 3035.}	14.155	0.0592124	newly 5 weeks spec	1603.	1554.07	{1454., 1644.}	4.44105	0.0201228	newly 5 weeks cumul	5687.	5839.51	{5239., 6371.}	7.02989	0.0302708	newly 64 weeks cumul	8914.	8821.81	{7764., 9179.}	4.03018	0.0183543
44	newly 3 weeks spec	783.	773.08	{649., 937.}	9.83397	0.0430364	newly 3 weeks cumul	2116.	2042.22	{1753., 2420.}	9.60586	0.0429547	newly 5 weeks spec	810.	684.65	{591., 829.}	17.2531	0.0836398	newly 5 weeks cumul	3802.	3496.34	{3054., 4197.}	11.6802	0.0538223	newly 55 weeks cumul	7747.	5726.93	{5057., 8045.}	27.4131	0.142897
45	newly 3 weeks spec	892.	846.17	{504., 972.}	13.0168	0.0735461	newly 3 weeks cumul	2335.	2209.43	{1652., 2483.}	9.29036	0.0505269	newly 5 weeks spec	873.	774.91	{266., 923.}	15.3482	0.106552	newly 5 weeks cumul	4172.	3830.83	{2247., 4326.}	10.564	0.0628204	newly 59 weeks cumul	7795.	6657.06	{2563., 7597.}	14.5983	0.0961793
46	newly 3 weeks spec	811.	889.78	{836., 928.}	9.83724	0.0403994	newly 3 weeks cumul	2110.	2252.99	{2159., 2333.}	6.83555	0.0285251	newly 5 weeks spec	861.	862.01	{813., 899.}	3.18351	0.0138404	newly 5 weeks cumul	3857.	4028.61	{3902., 4130.}	4.50791	0.0190382	newly 57 weeks cumul	7935.	7492.05	{7114., 7905.}	5.61449	0.0255411
47	newly 3 weeks spec	1061.	1041.95	{814., 1297.}	14.2988	0.0659534	newly 3 weeks cumul	2374.	2466.36	{1837., 2984.}	15.8231	0.0698788	newly 5 weeks spec	1104.	757.34	{597., 987.}	31.4004	0.174065	newly 5 weeks cumul	4682.	4212.45	{3456., 5057.}	13.3505	0.0647177	newly 59 weeks cumul	8780.	5589.25	{4765., 6596.}	36.3411	0.202618
48	newly 3 weeks spec	1085.	1118.38	{983., 1231.}	8.42765	0.0357619	newly 3 weeks cumul	2381.	2445.14	{2097., 2724.}	8.55019	0.0364246	newly 5 weeks spec	1180.	1122.69	{974., 1222.}	6.62458	0.0326116	newly 5 weeks cumul	4857.	4790.33	{4316., 5142.}	5.63208	0.0250281	newly 62 weeks cumul	8650.	8067.1	{6609., 8878.}	7.9896	0.0391874
49	newly 3 weeks spec	1202.	1225.17	{1062., 1376.}	9.11065	0.0405628	newly 3 weeks cumul	2426.	2457.05	{2021., 2847.}	9.33924	0.040744	newly 5 weeks spec	1386.	1293.98	{1210., 1380.}	6.93362	0.0352063	newly 5 weeks cumul	5269.	5159.86	{4616., 5647.}	5.96812	0.0280995	newly 63 weeks cumul	8545.	8395.72	{7913., 8896.}	6.25044	0.0307094
50	newly 3 weeks spec	1062.	873.59	{793., 974.}	17.8164	0.0867726	newly 3 weeks cumul	2104.	1820.5	{1639., 2036.}	14.0323	0.0668159	newly 5 weeks spec	1231.	1128.12	{1066., 1194.}	8.62226	0.0395467	newly 5 weeks cumul	4630.	4023.3	{3730., 4399.}	13.1927	0.0622538	newly 61 weeks cumul	8721.	8623.93	{7975., 8969.}	2.96216	0.0132367
