# Supplementary material for: A Likelihood Approach for Real-Time Calibration of Stochastic Compartmental Epidemic Models
Source: PLoS Comput Biol. 2017 Jan 17;13(1):e1005257. doi: 10.1371/journal.pcbi.1005257 (PMC5240920; doi:10.1371/journal.pcbi.1005257)
Supplement: S1 File — (TAR.GZ) [file pcbi.1005257.s014.tar.gz › HSPH_Online-SI-Revision/MSS10/n10-extreme/n10-extreme_NEW2_table4.pdf]

1	newly	3 weeks	spec	135.	125.66	{109., 141.}	9.57037	0.0442376	newly	3 weeks	cumul	629.	614.03	{588., 632.}	2.87599	0.0127499	newly	5 weeks	spec	61.	50.87	{41., 60.}	17.8852	0.0890595	newly	5 weeks	cumul	793.	744.29	{715., 764.}	6.1425	0.0276611	newly	54 weeks	cumul	895.	833.77	{805., 861.}	6.84134	0.0309403
2	newly	3 weeks	spec	274.	182.71	{158., 209.}	33.3175	0.17793	newly	3 weeks	cumul	1010.	746.63	{707., 790.}	26.0762	0.131693	newly	5 weeks	spec	172.	99.36	{80., 120.}	42.2326	0.243042	newly	5 weeks	cumul	1394.	982.74	{925., 1051.}	29.5022	0.152393	newly	50 weeks	cumul	1905.	1236.56	{1133., 1335.}	35.0887	0.188506
3	newly	3 weeks	spec	87.	71.15	{57., 95.}	21.7126	0.108501	newly	3 weeks	cumul	473.	409.04	{372., 503.}	16.3214	0.0772096	newly	5 weeks	spec	29.	23.69	{16., 32.}	25.5517	0.131662	newly	5 weeks	cumul	554.	474.36	{427., 603.}	17.4152	0.0828334	newly	56 weeks	cumul	592.	507.16	{455., 638.}	17.8716	0.084978
4	newly	3 weeks	spec	199.	202.95	{185., 218.}	5.87437	0.0250448	newly	3 weeks	cumul	783.	910.83	{878., 942.}	16.3257	0.0655364	newly	5 weeks	spec	95.	94.89	{81., 110.}	9.98947	0.0437026	newly	5 weeks	cumul	1007.	1143.74	{1107., 1186.}	13.5789	0.0551635	newly	50 weeks	cumul	1308.	1339.11	{1298., 1379.}	2.92125	0.0124571
5	newly	3 weeks	spec	213.	207.77	{187., 228.}	6.52113	0.0293134	newly	3 weeks	cumul	916.	866.07	{802., 905.}	5.45961	0.0247846	newly	5 weeks	spec	132.	110.29	{97., 123.}	16.6591	0.0807716	newly	5 weeks	cumul	1203.	1129.36	{1049., 1171.}	6.12136	0.0277661	newly	53 weeks	cumul	1516.	1420.68	{1318., 1467.}	6.2876	0.028518
6	newly	3 weeks	spec	232.	185.86	{167., 201.}	19.8879	0.0974522	newly	3 weeks	cumul	932.	813.87	{786., 840.}	12.6749	0.0590037	newly	5 weeks	spec	93.	90.45	{80., 104.}	8.93548	0.0399698	newly	5 weeks	cumul	1166.	1034.94	{1006., 1064.}	11.2401	0.0518886	newly	54 weeks	cumul	1415.	1245.12	{1216., 1276.}	12.0057	0.0556388
7	newly	3 weeks	spec	196.	133.54	{114., 153.}	31.8673	0.16944	newly	3 weeks	cumul	905.	659.33	{617., 702.}	27.1459	0.138207	newly	5 weeks	spec	97.	54.91	{42., 69.}	43.3918	0.254303	newly	5 weeks	cumul	1165.	801.08	{755., 851.}	31.2378	0.16323	newly	55 weeks	cumul	1418.	895.76	{838., 960.}	36.8293	0.200172
8	newly	3 weeks	spec	285.	245.02	{224., 270.}	14.0702	0.0669816	newly	3 weeks	cumul	1128.	1058.72	{1026., 1085.}	6.1578	0.0277031	newly	5 weeks	spec	172.	121.76	{107., 135.}	29.2093	0.151772	newly	5 weeks	cumul	1506.	1355.35	{1327., 1392.}	10.0033	0.045856	newly	52 weeks	cumul	1911.	1636.3	{1595., 1675.}	14.3747	0.0674796
9	newly	3 weeks	spec	224.	252.18	{218., 291.}	13.7321	0.0541389	newly	3 weeks	cumul	973.	1017.91	{932., 1124.}	7.50154	0.0309574	newly	5 weeks	spec	131.	140.33	{117., 166.}	12.5573	0.0511422	newly	5 weeks	cumul	1278.	1348.35	{1229., 1496.}	7.88811	0.0322444	newly	52 weeks	cumul	1632.	1742.66	{1566., 1949.}	8.86642	0.0358132
10	newly	3 weeks	spec	311.	259.25	{239., 280.}	16.6399	0.0798454	newly	3 weeks	cumul	1154.	997.48	{970., 1029.}	13.5633	0.0634229	newly	5 weeks	spec	185.	161.52	{144., 177.}	12.9297	0.061504	newly	5 weeks	cumul	1603.	1364.43	{1322., 1397.}	14.8827	0.0700776	newly	52 weeks	cumul	2165.	1890.13	{1842., 1933.}	12.6961	0.0590514
11	newly	3 weeks	spec	280.	169.07	{152., 186.}	39.6179	0.220386	newly	3 weeks	cumul	1095.	734.68	{701., 765.}	32.9059	0.17355	newly	5 weeks	spec	155.	81.31	{67., 96.}	47.5419	0.284431	newly	5 weeks	cumul	1475.	933.17	{889., 977.}	36.7342	0.199112	newly	47 weeks	cumul	1957.	1121.17	{1059., 1173.}	42.7098	0.242275
12	newly	3 weeks	spec	241.	207.49	{186., 229.}	13.9627	0.0665366	newly	3 weeks	cumul	1116.	931.07	{894., 968.}	16.5708	0.0788856	newly	5 weeks	spec	113.	96.46	{79., 113.}	15.1327	0.0741013	newly	5 weeks	cumul	1420.	1169.46	{1122., 1213.}	17.6437	0.0845117	newly	52 weeks	cumul	1677.	1368.46	{1304., 1420.}	18.3983	0.0885507
13	newly	3 weeks	spec	180.	206.11	{187., 228.}	14.6944	0.0584478	newly	3 weeks	cumul	742.	770.06	{742., 794.}	4.04852	0.0171374	newly	5 weeks	spec	114.	130.9	{114., 145.}	15.6667	0.0619985	newly	5 weeks	cumul	985.	1065.93	{1036., 1098.}	8.21624	0.0341578	newly	48 weeks	cumul	1277.	1550.3	{1493., 1605.}	21.4017	0.0840626
14	newly	3 weeks	spec	185.	203.48	{183., 225.}	10.6378	0.0430919	newly	3 weeks	cumul	825.	829.27	{798., 857.}	2.14182	0.00927216	newly	5 weeks	spec	104.	111.46	{94., 129.}	12.0192	0.0495126	newly	5 weeks	cumul	1066.	1092.03	{1059., 1130.}	2.94841	0.0125643	newly	53 weeks	cumul	1314.	1405.13	{1305., 1470.}	7.3828	0.0306969
15	newly	3 weeks	spec	149.	89.25	{75., 103.}	40.1007	0.226043	newly	3 weeks	cumul	603.	400.31	{361., 436.}	33.6136	0.179069	newly	5 weeks	spec	76.	45.28	{34., 58.}	40.4211	0.236628	newly	5 weeks	cumul	785.	509.38	{462., 557.}	35.1108	0.189099	newly	50 weeks	cumul	962.	610.43	{545., 662.}	36.5457	0.198849
16	newly	3 weeks	spec	236.	192.93	{174., 211.}	18.25	0.0887177	newly	3 weeks	cumul	954.	868.14	{841., 897.}	9.10273	0.0415663	newly	5 weeks	spec	131.	89.52	{76., 101.}	31.6641	0.168663	newly	5 weeks	cumul	1261.	1091.35	{1058., 1121.}	13.525	0.0632237	newly	53 weeks	cumul	1555.	1280.29	{1240., 1310.}	17.7717	0.0851271
17	newly	3 weeks	spec	218.	219.75	{145., 247.}	11.1239	0.052642	newly	3 weeks	cumul	974.	970.4	{692., 1040.}	7.65708	0.0363554	newly	5 weeks	spec	107.	108.63	{72., 128.}	14.9439	0.0724482	newly	5 weeks	cumul	1233.	1232.74	{856., 1323.}	8.62125	0.0410646	newly	55 weeks	cumul	1481.	1468.87	{971., 1583.}	8.90952	0.0436475
18	newly	3 weeks	spec	202.	170.33	{154., 188.}	15.7673	0.0759377	newly	3 weeks	cumul	887.	775.99	{752., 797.}	12.5152	0.0581789	newly	5 weeks	spec	107.	78.87	{67., 92.}	26.2897	0.135491	newly	5 weeks	cumul	1132.	971.65	{946., 1001.}	14.1652	0.0664401	newly	53 weeks	cumul	1431.	1141.21	{1107., 1173.}	20.2509	0.0984025
19	newly	3 weeks	spec	155.	132.21	{115., 151.}	14.7677	0.0717248	newly	3 weeks	cumul	791.	673.2	{636., 708.}	14.8925	0.0703644	newly	5 weeks	spec	62.	50.73	{40., 62.}	19.3065	0.0986549	newly	5 weeks	cumul	957.	803.91	{761., 851.}	15.9969	0.0760892	newly	55 weeks	cumul	1067.	881.31	{840., 933.}	17.403	0.0834681
20	newly	3 weeks	spec	277.	223.98	{203., 247.}	19.1408	0.0936225	newly	3 weeks	cumul	1115.	929.87	{900., 961.}	16.6036	0.0790652	newly	5 weeks	spec	154.	119.93	{102., 133.}	22.1234	0.111517	newly	5 weeks	cumul	1485.	1216.	{1184., 1255.}	18.1145	0.0870528	newly	52 weeks	cumul	1930.	1527.73	{1480., 1577.}	20.843	0.101921
21	newly	3 weeks	spec	230.	135.28	{121., 151.}	41.1826	0.232292	newly	3 weeks	cumul	931.	542.12	{520., 568.}	41.7701	0.235146	newly	5 weeks	spec	137.	74.93	{60., 89.}	45.3066	0.266912	newly	5 weeks	cumul	1250.	719.55	{685., 754.}	42.436	0.240116	newly	49 weeks	cumul	1622.	933.17	{870., 990.}	42.4679	0.240575
22	newly	3 weeks	spec	126.	84.92	{71., 98.}	32.6032	0.174546	newly	3 weeks	cumul	596.	467.81	{448., 491.}	21.5084	0.105455	newly	5 weeks	spec	39.	32.34	{24., 40.}	19.8974	0.100909	newly	5 weeks	cumul	702.	551.66	{525., 577.}	21.416	0.104954	newly	56 weeks	cumul	785.	597.53	{564., 625.}	23.8815	0.118836
23	newly	3 weeks	spec	183.	299.68	{274., 326.}	63.7596	0.213326	newly	3 weeks	cumul	736.	1094.37	{1062., 1130.}	48.6916	0.172159	newly	5 weeks	spec	84.	196.38	{181., 217.}	133.786	0.367575	newly	5 weeks	cumul	938.	1534.67	{1484., 1580.}	63.6109	0.213683	newly	51 weeks	cumul	1140.	2274.89	{2200., 2349.}	99.5518	0.299915
24	newly	3 weeks	spec	218.	241.53	{223., 262.}	11.1697	0.0452875	newly	3 weeks	cumul	857.	954.2	{931., 976.}	11.3419	0.0465808	newly	5 weeks	spec	150.	140.5	{127., 154.}	8.14667	0.0375093	newly	5 weeks	cumul	1186.	1278.81	{1250., 1306.}	7.82546	0.0326665	newly	49 weeks	cumul	1579.	1708.69	{1678., 1748.}	8.21343	0.034231
25	newly	3 weeks	spec	123.	63.78	{51., 74.}	48.1463	0.289993	newly	3 weeks	cumul	613.	362.29	{336., 386.}	40.8989	0.229043	newly	5 weeks	spec	53.	22.37	{14., 28.}	57.7925	0.388228	newly	5 weeks	cumul	719.	421.87	{387., 453.}	41.3255	0.232283	newly	52 weeks	cumul	805.	451.27	{411., 488.}	43.9416	0.252248
26	newly	3 weeks	spec	82.	54.53	{43., 66.}	33.5	0.183296	newly	3 weeks	cumul	370.	303.39	{272., 334.}	18.0027	0.0875265	newly	5 weeks	spec	10.	18.98	{11., 28.}	92.2	0.264688	newly	5 weeks	cumul	409.	354.04	{312., 389.}	13.7066	0.0653477	newly	55 weeks	cumul	439.	380.	{327., 420.}	13.7995	0.0661977
27	newly	3 weeks	spec	253.	209.62	{180., 247.}	20.0395	0.0973699	newly	3 weeks	cumul	1055.	903.05	{842., 1023.}	16.1697	0.0766479	newly	5 weeks	spec	136.	103.24	{83., 127.}	26.9853	0.138785	newly	5 weeks	cumul	1391.	1149.52	{1067., 1329.}	19.1402	0.0924351	newly	53 weeks	cumul	1730.	1388.51	{1275., 1650.}	22.2618	0.109143
28	newly	3 weeks	spec	143.	139.42	{120., 156.}	7.42657	0.0336601	newly	3 weeks	cumul	714.	691.58	{664., 726.}	4.12325	0.0191138	newly	5 weeks	spec	59.	55.31	{46., 67.}	12.8644	0.0609994	newly	5 weeks	cumul	874.	833.33	{818., 874.}	4.77002	0.0223212	newly	56 weeks	cumul	975.	927.94	{903., 973.}	4.98256	0.0234213
29	newly	3 weeks	spec	161.	142.19	{127., 158.}	12.1677	0.0574862	newly	3 weeks	cumul	747.	700.06	{679., 725.}	6.2838	0.0283186	newly	5 weeks	spec	83.	55.71	{46., 64.}	32.8795	0.176739	newly	5 weeks	cumul	974.	843.55	{817., 870.}	13.3932	0.0625775	newly	51 weeks	cumul	1110.	937.3	{900., 967.}	15.5586	0.0736062
30	newly	3 weeks	spec	187.	142.8	{125., 159.}	23.6364	0.119013	newly	3 weeks	cumul	802.	646.64	{606., 678.}	19.3716	0.0938641	newly	5 weeks	spec	78.	63.89	{52., 75.}	19.0641	0.0957441	newly	5 weeks	cumul	1008.	804.2	{758., 847.}	20.2183	0.0984553	newly	51 weeks	cumul	1234.	934.58	{883., 986.}	24.2642	0.121078
31	newly	3 weeks	spec	252.	217.11	{198., 235.}	13.8849	0.0658842	newly	3 weeks	cumul	969.	954.65	{926., 977.}	1.96388	0.00864901	newly	5 weeks	spec	111.	109.25	{96., 121.}	7.45045	0.0332436	newly	5 weeks	cumul	1242.	1218.31	{1182., 1250.}	2.26006	0.00998314	newly	54 weeks	cumul	1508.	1466.35	{1416., 1507.}	2.91711	0.0129935
32	newly	3 weeks	spec	205.	166.16	{152., 183.}	18.9463	0.0923466	newly	3 weeks	cumul	891.	626.5	{598., 649.}	29.6857	0.153237	newly	5 weeks	spec	111.	105.99	{92., 120.}	8.20721	0.0376173	newly	5 weeks	cumul	1156.	863.78	{825., 898.}	25.2785	0.126782	newly	46 weeks	cumul	1578.	1235.64	{1175., 1288.}	21.6958	0.106493
33	newly	3 weeks	spec	80.	55.24	{45., 64.}	30.95	0.165125	newly	3 weeks	cumul	415.	300.61	{283., 320.}	27.5639	0.140478	newly	5 weeks	spec	25.	18.16	{11., 25.}	30.56	0.171856	newly	5 weeks	cumul	481.	350.19	{331., 370.}	27.1954	0.138237	newly	54 weeks	cumul	518.	375.9	{352., 399.}	27.4324	0.139728
34	newly	3 weeks	spec	107.	66.7	{54., 81.}	37.6636	0.210529	newly	3 weeks	cumul	518.	350.87	{326., 378.}	32.2645	0.170013	newly	5 weeks	spec	37.	24.43	{17., 31.}	34.8378	0.196792	newly	5 weeks	cumul	629.	415.01	{383., 449.}	34.0207	0.181539	newly	56 weeks	cumul	672.	451.34	{416., 497.}	32.8363	0.173994
35	newly	3 weeks	spec	219.	213.27	{195., 236.}	6.15068	0.0273455	newly	3 weeks	cumul	914.	866.96	{838., 891.}	5.15098	0.0231052	newly	5 weeks	spec	143.	118.96	{102., 137.}	17.021	0.0836497	newly	5 weeks	cumul	1246.	1147.25	{1108., 1187.}	7.92536	0.0360238	newly	51 weeks	cumul	1672.	1467.67	{1407., 1542.}	12.2207	0.0568276
36	newly	3 weeks	spec	304.	135.53	{121., 152.}	55.4178	0.352614	newly	3 weeks	cumul	1147.	566.16	{540., 589.}	50.6399	0.306897	newly	5 weeks	spec	161.	70.92	{56., 83.}	55.9503	0.360589	newly	5 weeks	cumul	1520.	736.67	{701., 767.}	51.5349	0.314828	newly	49 weeks	cumul	2076.	923.51	{869., 970.}	55.5149	0.352215
37	newly	3 weeks	spec	209.	184.84	{168., 206.}	11.7129	0.0551344	newly	3 weeks	cumul	875.	781.08	{755., 804.}	10.7337	0.0494394	newly	5 weeks	spec	117.	95.83	{83., 108.}	18.265	0.089671	newly	5 weeks	cumul	1151.	1011.96	{981., 1037.}	12.0799	0.0560054	newly	52 weeks	cumul	1404.	1250.07	{1211., 1290.}	10.9637	0.0505706
38	newly	3 weeks	spec	55.	41.49	{31., 52.}	24.7818	0.13066	newly	3 weeks	cumul	284.	259.49	{240., 279.}	8.76408	0.0405482	newly	5 weeks	spec	18.	12.75	{8., 19.}	33.9444	0.19526	newly	5 weeks	cumul	331.	295.44	{271., 319.}	10.7734	0.0503584	newly	55 weeks	cumul	350.	309.66	{282., 338.}	11.6457	0.0547788
39	newly	3 weeks	spec	107.	90.18	{80., 102.}	16.0748	0.0781223	newly	3 weeks	cumul	564.	462.07	{442., 479.}	18.0727	0.0867772	newly	5 weeks	spec	42.	36.82	{29., 45.}	16.1429	0.0790893	newly	5 weeks	cumul	669.	555.75	{531., 577.}	16.9283	0.0807539	newly	55 weeks	cumul	739.	616.46	{591., 645.}	16.5819	0.0789776
40	newly	3 weeks	spec	222.	162.2	{130., 177.}	33.8288	0.175675	newly	3 weeks	cumul	944.	704.61	{627., 709.}	29.7807	0.151843	newly	5 weeks	spec	115.	80.36	{59., 92.}	39.0783	0.208879	newly	5 weeks	cumul	1200.	897.81	{793., 886.}	30.8058	0.156592	newly	52 weeks	cumul	1524.	1090.42	{938., 1054.}	35.5354	0.185129
41	newly	3 weeks	spec	212.	250.04	{229., 271.}	17.9434	0.0707323	newly	3 weeks	cumul	904.	1037.14	{1014., 1063.}	14.7279	0.0595958	newly	5 weeks	spec	89.	136.34	{122., 150.}	53.191	0.184009	newly	5 weeks	cumul	1155.	1360.56	{1332., 1386.}	17.7974	0.0710902	newly	51 weeks	cumul	1447.	1726.5	{1700., 1751.}	19.3158	0.0766681
42	newly	3 weeks	spec	235.	182.98	{166., 201.}	22.1362	0.109997	newly	3 weeks	cumul	936.	780.68	{747., 813.}	16.594	0.0790138	newly	5 weeks	spec	165.	94.12	{82., 106.}	42.9576	0.246115	newly	5 weeks	cumul	1287.	1006.08	{972., 1038.}	21.8275	0.107084	newly	46 weeks	cumul	1777.	1234.79	{1188., 1276.}	30.5127	0.158252
43	newly	3 weeks	spec	116.	128.57	{112., 146.}	13.2328	0.0532468	newly	3 weeks	cumul	596.	683.67	{658., 720.}	16.2164	0.0655142	newly	5 weeks	spec	40.	47.62	{36., 59.}	23.5	0.0887918	newly	5 weeks	cumul	704.	809.25	{790., 845.}	16.5412	0.0668259	newly	56 weeks	cumul	757.	879.42	{848., 925.}	17.7754	0.0713689
44	newly	3 weeks	spec	281.	177.63	{158., 195.}	36.7865	0.200736	newly	3 weeks	cumul	1071.	728.58	{702., 763.}	31.972	0.167544	newly	5 weeks	spec	173.	99.27	{86., 113.}	42.6185	0.243974	newly	5 weeks	cumul	1453.	962.	{923., 998.}	33.7922	0.179279	newly	47 weeks	cumul	1985.	1237.08	{1181., 1295.}	37.6786	0.205596
45	newly	3 weeks	spec	250.	222.68	{207., 240.}	10.976	0.0511836	newly	3 weeks	cumul	978.	914.39	{884., 943.}	6.50409	0.0293287	newly	5 weeks	spec	142.	120.57	{105., 134.}	15.2465	0.0734969	newly	5 weeks	cumul	1328.	1198.07	{1167., 1234.}	9.78389	0.0448166	newly	51 weeks	cumul	1725.	1506.31	{1455., 1556.}	12.6777	0.0590091
46	newly	3 weeks	spec	267.	235.35	{216., 254.}	11.9213	0.0560262	newly	3 weeks	cumul	1078.	1002.81	{972., 1033.}	6.97495	0.0315249	newly	5 weeks	spec	173.	118.4	{102., 131.}	31.5607	0.16709	newly	5 weeks	cumul	1493.	1289.29	{1250., 1324.}	13.6443	0.0638146	newly	49 weeks	cumul	2102.	1560.64	{1504., 1610.}	25.7545	0.129481
47	newly	3 weeks	spec	255.	256.33	{237., 274.}	4.66275	0.0201512	newly	3 weeks	cumul	1074.	1036.52	{1008., 1060.}	3.5419	0.0157508	newly	5 weeks	spec	129.	145.49	{127., 161.}	13.8062	0.0552344	newly	5 weeks	cumul	1366.	1376.86	{1348., 1406.}	1.57394	0.00680204	newly	51 weeks	cumul	1768.	1796.79	{1770., 1829.}	2.04242	0.00877709
48	newly	3 weeks	spec	231.	181.78	{163., 201.}	21.3074	0.105634	newly	3 weeks	cumul	980.	839.06	{809., 861.}	14.3816	0.0676346	newly	5 weeks	spec	109.	81.42	{69., 96.}	25.3394	0.13035	newly	5 weeks	cumul	1224.	1043.51	{1006., 1081.}	14.7459	0.0695015	newly	54 weeks	cumul	1459.	1202.96	{1162., 1245.}	17.549	0.084062
49	newly	3 weeks	spec	157.	136.99	{120., 152.}	13.3439	0.0634122	newly	3 weeks	cumul	774.	698.7	{671., 725.}	9.72868	0.0446522	newly	5 weeks	spec	60.	51.13	{42., 61.}	16.55	0.0818824	newly	5 weeks	cumul	908.	834.77	{807., 865.}	8.06498	0.0367053	newly	55 weeks	cumul	1013.	916.49	{883., 951.}	9.52715	0.0437245
50	newly	3 weeks	spec	221.	219.43	{202., 235.}	4.8552	0.021309	newly	3 weeks	cumul	1020.	926.11	{902., 949.}	9.2049	0.0420303	newly	5 weeks	spec	108.	116.	{99., 129.}	10.7963	0.0447259	newly	5 weeks	cumul	1290.	1202.73	{1175., 1230.}	6.76512	0.0304894	newly	53 weeks	cumul	1505.	1488.48	{1451., 1514.}	1.52159	0.00668172
