# Supplementary material for: A Likelihood Approach for Real-Time Calibration of Stochastic Compartmental Epidemic Models
Source: PLoS Comput Biol. 2017 Jan 17;13(1):e1005257. doi: 10.1371/journal.pcbi.1005257 (PMC5240920; doi:10.1371/journal.pcbi.1005257)
Supplement: S1 File — (TAR.GZ) [file pcbi.1005257.s014.tar.gz › HSPH_Online-SI-Revision/MSS10/n10-extreme/n10-extreme_NEW_table8.pdf]

1	R0	{2.68692, 12.5884, 1.}	{2.53075, 11.6597, 0.}	{{2.53075, 11.6597, 0.}, {2.53075, 11.6597, 0.}}	{5.81226, 7.37731, 100.}	inf	138.	70.8799	{65.4144, 75.7575}	48.6377	0.290181	newly	61.	33.92	{27., 41.}	44.3934	0.26016	Reff	0.251496	0.253865	{0.25229, 0.255218}	0.947003	ar	9089.	9020.85	{9008., 9031.}	0.749807
2	R0	{1.79802, 12.5412, 1.}	{1.71608, 9.75208, 0.}	{{1.71608, 9.75208, 0.}, {1.71608, 9.75208, 0.}}	{4.55731, 22.2397, 100.}	inf	348.	302.065	{285.327, 317.575}	13.1998	0.0617969	newly	172.	181.66	{166., 199.}	7.66279	0.0318261	Reff	0.525561	0.509493	{0.506793, 0.512343}	3.05742	ar	7412.	7314.29	{7257., 7373.}	1.33662
3	R0	{2.6174, 10.1105, 0.1}	{2.64283, 10.0587, 3.87}	{{2.60471, 9.67274, 3.}, {2.64852, 10.1164, 4.}}	{1.09745, 0.613802, 3770.}	inf	48.	69.9545	{61.5808, 75.728}	45.7385	0.161993	newly	29.	37.33	{31., 44.}	29.2759	0.107934	Reff	0.238183	0.234688	{0.233423, 0.235964}	1.46742	ar	9109.	9134.83	{9121., 9149.}	0.284224
4	R0	{2.27532, 14.3149, 0.1}	{2.03251, 13.7179, 3.}	{{2.03251, 13.7179, 3.}, {2.03251, 13.7179, 3.}}	{10.6714, 4.17058, 2900.}	inf	238.	190.703	{182.337, 199.382}	19.8727	0.0964534	newly	95.	83.07	{71., 94.}	13.3158	0.0638619	Reff	0.382254	0.351075	{0.349241, 0.35273}	8.1565	ar	8478.	8370.35	{8346., 8393.}	1.26976
5	R0	{2.96649, 18.0674, 2.}	{2.96398, 17.7348, 2.}	{{2.96398, 17.7348, 2.}, {2.96398, 17.7348, 2.}}	{0.0846464, 1.84086, 0.}	inf	369.	294.197	{288.353, 299.652}	20.2719	0.09844	newly	132.	100.32	{91., 108.}	24.	0.120281	Reff	0.177989	0.20001	{0.198313, 0.201655}	12.372	ar	9476.	9393.55	{9378., 9410.}	0.870093
6	R0	{2.94778, 16.0002, 0.1}	{2.96947, 15.963, 0.}	{{2.96947, 15.963, 0.}, {2.96947, 15.963, 0.}}	{0.735864, 0.232734, 100.}	inf	276.	250.597	{243.896, 258.502}	9.20415	0.042063	newly	93.	93.26	{83., 103.}	6.90323	0.029897	Reff	0.21961	0.228769	{0.226101, 0.230733}	4.17082	ar	9321.	9299.7	{9284., 9319.}	0.234739
7	R0	{2.17562, 11.7184, 1.}	{2.22048, 13.4379, 5.}	{{2.21449, 13.2892, 5.}, {2.22518, 13.5548, 5.}}	{2.06188, 14.6737, 400.}	inf	199.	333.664	{318.716, 352.574}	67.6702	0.224164	newly	97.	147.28	{130., 160.}	51.8351	0.179948	Reff	0.392917	0.371121	{0.365844, 0.375035}	5.54731	ar	8345.	8512.2	{8476., 8543.}	2.00359
8	R0	{2.7904, 17.8966, 2.}	{2.60465, 15.5928, 1.}	{{2.60465, 15.5928, 1.}, {2.60465, 15.5928, 1.}}	{6.65672, 12.8729, 50.}	inf	456.	402.855	{392.858, 412.066}	11.6545	0.0538969	newly	172.	152.85	{138., 165.}	11.2616	0.0527682	Reff	0.233277	0.231591	{0.229103, 0.234118}	0.942815	ar	9285.	9223.27	{9202., 9245.}	0.665051
9	R0	{2.51574, 16.376, 1.}	{2.53741, 16.1789, 2.}	{{2.53741, 16.1789, 2.}, {2.53741, 16.1789, 2.}}	{0.861236, 1.20329, 100.}	inf	368.	322.502	{310.563, 331.369}	12.3636	0.0574333	newly	131.	119.9	{107., 131.}	9.40458	0.0437657	Reff	0.296857	0.310959	{0.308514, 0.313703}	4.75021	ar	8937.	8905.9	{8883., 8926.}	0.349782
10	R0	{2.56405, 18.1787, 2.}	{2.41419, 18.743, 4.}	{{2.41419, 18.743, 4.}, {2.41419, 18.743, 4.}}	{5.84452, 3.10436, 100.}	inf	542.	507.668	{494.988, 523.162}	6.33425	0.0285048	newly	185.	165.73	{151., 180.}	10.6973	0.0499489	Reff	0.307686	0.297992	{0.294219, 0.300844}	3.15076	ar	9005.	8957.85	{8931., 8981.}	0.525597
11	R0	{2.475, 17.1709, 1.}	{1.81391, 11.3493, 1.}	{{1.81391, 11.3493, 1.}, {1.81391, 11.3493, 1.}}	{26.7106, 33.9041, 0.}	inf	441.	77.5555	{68.0767, 85.9495}	82.4137	0.756701	newly	155.	38.96	{31., 46.}	74.8645	0.605258	Reff	0.328185	0.30645	{0.304886, 0.308071}	6.62267	ar	8870.	8344.85	{8323., 8364.}	5.92052
12	R0	{2.14974, 11.8619, 0.1}	{2.73517, 19.6025, 2.}	{{2.73517, 19.6025, 2.}, {2.73517, 19.6025, 2.}}	{27.2326, 65.2561, 1900.}	inf	247.	694.222	{683.723, 705.183}	181.062	0.448766	newly	113.	220.37	{205., 236.}	95.0177	0.289488	Reff	0.367391	0.345118	{0.342022, 0.3479}	6.06245	ar	8414.	9036.32	{9004., 9068.}	7.39624
13	R0	{2.20385, 14.4767, 0.1}	{2.46477, 18.9032, 1.}	{{2.46477, 18.9032, 1.}, {2.46477, 18.9032, 1.}}	{11.8393, 30.5765, 900.}	inf	257.	462.291	{449.449, 474.227}	79.8799	0.254897	newly	114.	152.95	{138., 165.}	34.1667	0.126703	Reff	0.397134	0.393552	{0.390588, 0.396573}	0.92304	ar	8347.	8665.96	{8621., 8709.}	3.82125
14	R0	{2.35671, 13.8357, 1.}	{2.30241, 14.8271, 4.}	{{2.28604, 14.5901, 4.}, {2.41197, 16.4137, 4.}}	{2.91361, 7.16571, 300.}	inf	237.	254.969	{234.188, 327.542}	8.19857	0.0317841	newly	104.	102.75	{90., 115.}	7.54808	0.0331847	Reff	0.328997	0.317155	{0.314423, 0.319746}	3.59942	ar	8719.	8733.41	{8694., 8825.}	0.333869
15	R0	{1.91368, 10.1082, 1.}	{1.75445, 7.55154, 3.}	{{1.75445, 7.55154, 3.}, {1.75445, 7.55154, 3.}}	{8.32052, 25.293, 200.}	inf	135.	49.7493	{39.1342, 60.255}	63.1487	0.440596	newly	76.	36.44	{26., 46.}	52.0526	0.329083	Reff	0.503681	0.476729	{0.474885, 0.478588}	5.35101	ar	7486.	7328.38	{7303., 7354.}	2.10553
16	R0	{2.48727, 14.882, 0.1}	{2.5458, 15.1849, 2.}	{{2.5458, 15.1849, 2.}, {2.5458, 15.1849, 2.}}	{2.35316, 2.03504, 1900.}	inf	327.	446.71	{436.377, 456.356}	36.6086	0.135412	newly	131.	174.94	{160., 188.}	33.542	0.12477	Reff	0.299467	0.276038	{0.273556, 0.27865}	7.82351	ar	8894.	9067.88	{9046., 9091.}	1.95503
17	R0	{2.46795, 13.1915, 0.1}	{2.42221, 13.5694, 4.}	{{2.42221, 13.5694, 4.}, {2.42221, 13.5694, 4.}}	{1.85351, 2.86487, 3900.}	inf	235.	320.129	{309.311, 330.968}	36.2251	0.134118	newly	107.	138.75	{127., 153.}	29.6729	0.11172	Reff	0.326757	0.30008	{0.297323, 0.302622}	8.16402	ar	8796.	8888.01	{8868., 8908.}	1.04604
18	R0	{2.96603, 16.7543, 0.1}	{2.8833, 17.1847, 2.}	{{2.8833, 17.1847, 2.}, {2.8833, 17.1847, 2.}}	{2.78915, 2.56909, 1900.}	inf	304.	379.174	{374.024, 384.602}	24.7284	0.0959337	newly	107.	131.57	{118., 144.}	22.9626	0.0884804	Reff	0.209995	0.182463	{0.180889, 0.183932}	13.1108	ar	9394.	9444.89	{9432., 9456.}	0.541729
19	R0	{2.25216, 9.90465, 0.1}	{2.44675, 11.6528, 3.}	{{2.44675, 11.6528, 3.}, {2.44675, 11.6528, 3.}}	{8.63997, 17.6496, 2900.}	inf	114.	290.523	{279.63, 299.775}	154.845	0.406139	newly	62.	142.93	{131., 155.}	130.532	0.361746	Reff	0.321383	0.30596	{0.303674, 0.308259}	4.79903	ar	8631.	8865.04	{8841., 8891.}	2.71162
20	R0	{2.70837, 18.1923, 0.1}	{2.47514, 14.5291, 1.}	{{2.47514, 14.5291, 1.}, {2.47514, 14.5291, 1.}}	{8.6115, 20.1359, 900.}	inf	470.	368.775	{358.302, 378.516}	21.5373	0.105433	newly	154.	148.54	{138., 161.}	5.94805	0.0266791	Reff	0.256212	0.259203	{0.256762, 0.261781}	1.18607	ar	9183.	9072.07	{9049., 9094.}	1.20799
21	R0	{2.30288, 15.5877, 1.}	{2.11132, 12.644, 0.}	{{2.11132, 12.644, 0.}, {2.11132, 12.644, 0.}}	{8.31807, 18.8845, 100.}	inf	338.	412.684	{402.827, 423.577}	22.0957	0.0866191	newly	137.	188.61	{176., 200.}	37.6715	0.138185	Reff	0.344741	0.300297	{0.297962, 0.302275}	12.892	ar	8674.	8742.47	{8717., 8768.}	0.789371
22	R0	{2.82208, 10.7544, 0.1}	{2.80387, 10.6536, 0.}	{{2.80387, 10.6536, 0.}, {2.80387, 10.6536, 0.}}	{0.645389, 0.937217, 100.}	inf	91.	100.547	{94.6996, 105.51}	10.4907	0.0429742	newly	39.	51.49	{44., 60.}	32.0769	0.117667	Reff	0.196981	0.193033	{0.191335, 0.194509}	2.00426	ar	9333.	9336.44	{9326., 9349.}	0.0777885
23	R0	{1.69923, 10.1366, 1.}	{2.23964, 17.6755, 4.}	{{2.23964, 17.6755, 4.}, {2.23964, 17.6755, 4.}}	{31.8034, 74.3734, 300.}	inf	136.	812.702	{794.001, 829.444}	497.575	0.776325	newly	84.	292.14	{270., 310.}	247.786	0.540654	Reff	0.525912	0.541612	{0.537784, 0.545727}	2.98539	ar	7055.	8282.32	{8221., 8354.}	17.3965
24	R0	{2.64944, 19.054, 1.}	{2.76795, 19.6041, 1.}	{{2.76795, 19.6041, 1.}, {2.76795, 19.6041, 1.}}	{4.47298, 2.88729, 0.}	inf	411.	561.1	{550.181, 569.604}	36.5207	0.135159	newly	150.	175.36	{159., 187.}	16.9067	0.0670453	Reff	0.26097	0.231096	{0.228335, 0.233723}	11.4472	ar	9147.	9313.29	{9293., 9333.}	1.81797
25	R0	{2.53191, 10.9214, 0.1}	{2.37615, 9.11994, 4.}	{{2.37615, 9.11994, 4.}, {2.37615, 9.11994, 4.}}	{6.15175, 16.4948, 3900.}	inf	104.	43.4663	{38.0464, 48.6844}	58.2055	0.380654	newly	53.	25.29	{19., 31.}	52.283	0.328216	Reff	0.259774	0.258177	{0.256936, 0.259463}	0.625447	ar	9009.	8928.36	{8918., 8939.}	0.895105
26	R0	{1.89155, 6.76944, 0.1}	{1.83544, 5.79911, 5.}	{{1.83544, 5.78475, 5.}, {1.83544, 5.78475, 5.}}	{2.96629, 14.4674, 4900.}	inf	18.	7.86657	{2.75531, 11.0542}	68.1773	0.492689	newly	10.	6.86	{2., 10.}	52.6	0.325654	Reff	0.5058	0.492657	{0.492039, 0.49356}	2.59858	ar	7348.	7323.17	{7312., 7330.}	0.399156
27	R0	{2.63149, 15.6979, 0.1}	{2.5458, 15.1849, 2.}	{{2.5458, 15.1849, 2.}, {2.5458, 15.1849, 2.}}	{3.25635, 3.26824, 1900.}	inf	374.	307.744	{298.594, 315.692}	17.7155	0.0847919	newly	136.	119.5	{106., 131.}	12.4559	0.0590414	Reff	0.26236	0.270684	{0.268464, 0.272966}	3.17278	ar	9104.	9043.83	{9019., 9064.}	0.660918
28	R0	{2.85141, 12.6992, 1.}	{2.79531, 12.1249, 2.}	{{2.79531, 12.1249, 2.}, {2.79531, 12.1249, 2.}}	{1.9675, 4.52223, 100.}	inf	136.	101.525	{95.4912, 107.026}	25.3493	0.127443	newly	59.	47.71	{40., 56.}	19.339	0.0958689	Reff	0.201595	0.207265	{0.205726, 0.208898}	2.81281	ar	9317.	9285.61	{9273., 9297.}	0.336911
29	R0	{2.54244, 12.9085, 0.1}	{2.96797, 18.4042, 0.}	{{2.96797, 18.4042, 0.}, {2.96797, 18.4042, 0.}}	{16.7372, 42.5743, 100.}	inf	177.	472.934	{464.288, 481.2}	167.194	0.426783	newly	83.	155.54	{142., 166.}	87.3976	0.27193	Reff	0.256278	0.211339	{0.208784, 0.213878}	17.5352	ar	9034.	9403.19	{9385., 9421.}	4.08667
30	R0	{2.10982, 11.7636, 1.}	{2.02235, 10.6804, 0.}	{{2.02235, 10.6804, 0.}, {2.02235, 10.6804, 0.}}	{4.14563, 9.20804, 100.}	inf	182.	155.88	{144.946, 166.322}	14.3519	0.0677777	newly	78.	84.41	{75., 94.}	10.4231	0.0426187	Reff	0.400444	0.389125	{0.387001, 0.391305}	2.82648	ar	8224.	8172.98	{8143., 8202.}	0.620379
31	R0	{2.76312, 15.5396, 0.1}	{2.62879, 15.1183, 3.}	{{2.62879, 15.1183, 3.}, {2.62879, 15.1183, 3.}}	{4.86149, 2.71136, 2900.}	inf	308.	261.55	{253.874, 268.111}	15.0811	0.0711108	newly	111.	101.02	{91., 114.}	10.1261	0.0474654	Reff	0.234589	0.235395	{0.233572, 0.23737}	0.577347	ar	9220.	9183.24	{9161., 9200.}	0.398698
32	R0	{2.21727, 15.5439, 0.1}	{1.87294, 19.0269, 3.}	{{1.87294, 19.0269, 3.}, {1.87294, 19.0269, 3.}}	{15.5297, 22.4076, 2900.}	inf	307.	532.141	{521.705, 541.697}	73.336	0.238844	newly	111.	173.26	{160., 188.}	56.0901	0.192505	Reff	0.413964	0.30751	{0.305506, 0.309429}	25.7159	ar	8359.	8573.91	{8542., 8605.}	2.571
33	R0	{2.72912, 10.8159, 0.1}	{2.8186, 12.0293, 1.}	{{2.8186, 12.0293, 1.}, {2.8186, 12.0293, 1.}}	{3.27881, 11.2183, 900.}	inf	49.	110.806	{106.233, 115.442}	126.136	0.35413	newly	25.	51.32	{44., 59.}	105.28	0.309244	Reff	0.222969	0.212859	{0.21148, 0.214116}	4.53428	ar	9196.	9275.74	{9265., 9288.}	0.867116
34	R0	{2.20928, 10.1308, 1.}	{2.12684, 8.23415, 1.}	{{2.12684, 8.23415, 1.}, {2.12684, 8.23415, 1.}}	{3.73149, 18.7216, 0.}	inf	59.	96.9442	{87.3378, 106.064}	64.3123	0.214567	newly	37.	62.7	{54., 72.}	69.4595	0.226634	Reff	0.360775	0.339243	{0.337282, 0.341161}	5.96837	ar	8388.	8455.6	{8436., 8477.}	0.805913
35	R0	{2.10103, 15.2673, 2.}	{2.03831, 13.1693, 3.}	{{2.03831, 13.1693, 3.}, {2.03831, 13.1693, 3.}}	{2.98541, 13.7421, 50.}	inf	355.	170.331	{160.14, 180.348}	52.0194	0.319482	newly	143.	77.95	{67., 86.}	45.4895	0.265555	Reff	0.409701	0.435111	{0.433022, 0.437155}	6.20207	ar	8264.	7992.7	{7959., 8029.}	3.28291
36	R0	{2.74914, 19.2775, 1.}	{2.56757, 17.005, 0.}	{{2.56757, 17.005, 0.}, {2.56757, 17.005, 0.}}	{6.60448, 11.7885, 100.}	inf	508.	648.161	{638.127, 656.836}	27.5908	0.105794	newly	161.	225.9	{209., 241.}	40.3106	0.146463	Reff	0.249622	0.197148	{0.194916, 0.199666}	21.0212	ar	9301.	9371.68	{9353., 9392.}	0.759918
37	R0	{2.78161, 16.0136, 0.1}	{2.7499, 15.3124, 0.}	{{2.7499, 15.3124, 0.}, {2.7499, 15.3124, 0.}}	{1.14008, 4.3787, 100.}	inf	295.	111.83	{103.331, 118.446}	62.0916	0.421839	newly	117.	43.77	{36., 51.}	62.5897	0.431295	Reff	0.232264	0.279986	{0.278163, 0.282301}	20.5464	ar	9240.	9022.68	{9005., 9036.}	2.35195
38	R0	{2.4454, 8.33501, 0.1}	{2.41784, 8.02695, 4.}	{{2.41784, 8.02695, 4.}, {2.41784, 8.02695, 4.}}	{1.12696, 3.696, 3900.}	inf	27.	2.39661	{1.18539, 3.48485}	91.1237	1.08461	newly	18.	1.53	{0., 3.}	91.5	1.25464	Reff	0.282933	0.285693	{0.285399, 0.285984}	0.975561	ar	8853.	8819.48	{8817., 8822.}	0.378629
39	R0	{2.9154, 12.3861, 0.1}	{2.9884, 12.8326, 0.}	{{2.9884, 12.8326, 0.}, {2.9884, 12.8326, 0.}}	{2.50382, 3.60482, 100.}	inf	88.	35.7955	{31.654, 40.0789}	59.3233	0.392448	newly	42.	15.25	{11., 19.}	63.6905	0.450335	Reff	0.190376	0.210743	{0.209406, 0.211956}	10.6985	ar	9371.	9304.52	{9297., 9312.}	0.709423
40	R0	{2.40923, 15.1963, 0.1}	{2.16755, 14.8261, 4.}	{{2.16755, 14.8261, 4.}, {2.16755, 14.8261, 4.}}	{10.0312, 2.43606, 3900.}	inf	294.	198.472	{190.445, 206.276}	32.4926	0.170855	newly	115.	79.48	{69., 90.}	30.887	0.162743	Reff	0.339701	0.326331	{0.324596, 0.328045}	3.93581	ar	8735.	8586.63	{8564., 8609.}	1.69857
41	R0	{2.63137, 15.238, 1.}	{2.46794, 14.1192, 0.}	{{2.46794, 14.1192, 0.}, {2.46794, 14.1192, 0.}}	{6.21102, 7.34188, 100.}	inf	281.	240.084	{232.24, 248.609}	14.5608	0.0685052	newly	89.	99.46	{89., 111.}	12.5169	0.0500938	Reff	0.24998	0.244552	{0.242378, 0.246473}	2.1716	ar	9150.	9084.22	{9066., 9101.}	0.718907
42	R0	{2.48769, 19.4593, 0.1}	{2.01238, 15.0173, 1.}	{{2.01238, 15.0173, 1.}, {2.01238, 15.0173, 1.}}	{19.1063, 22.827, 900.}	inf	467.	307.384	{299.012, 317.709}	34.1791	0.181752	newly	165.	121.16	{110., 132.}	26.5697	0.135238	Reff	0.312454	0.284876	{0.282798, 0.286542}	8.82613	ar	8932.	8697.78	{8675., 8720.}	2.62226
43	R0	{2.61199, 10.214, 0.1}	{2.52681, 9.219, 1.}	{{2.52681, 9.219, 1.}, {2.52681, 9.219, 1.}}	{3.26125, 9.7415, 900.}	inf	68.	17.8058	{12.611, 22.3772}	73.815	0.591643	newly	40.	10.38	{6., 15.}	74.05	0.608343	Reff	0.251796	0.256267	{0.255067, 0.257429}	1.77581	ar	9061.	8992.15	{8983., 9000.}	0.75985
44	R0	{2.50994, 18.3382, 0.1}	{2.01238, 15.0173, 1.}	{{2.01238, 15.0173, 1.}, {2.01238, 15.0173, 1.}}	{19.8234, 18.109, 900.}	inf	483.	346.209	{335.064, 356.486}	28.3211	0.144727	newly	173.	137.13	{124., 148.}	20.7341	0.102052	Reff	0.350137	0.308255	{0.306173, 0.310476}	11.9615	ar	8827.	8612.15	{8583., 8640.}	2.43401
45	R0	{2.44283, 16.8338, 0.1}	{2.29887, 14.662, 2.}	{{2.29887, 14.662, 2.}, {2.29887, 14.662, 2.}}	{5.89311, 12.9013, 1900.}	inf	399.	168.666	{158.798, 179.373}	57.7279	0.374378	newly	142.	68.83	{59., 78.}	51.5282	0.316863	Reff	0.308285	0.343068	{0.340584, 0.345294}	11.2828	ar	8878.	8593.1	{8569., 8618.}	3.20906
46	R0	{2.51815, 18.1462, 5.}	{2.49092, 17.9391, 1.}	{{2.49092, 17.9391, 1.}, {2.49092, 17.9391, 1.}}	{1.08135, 1.14119, 80.}	inf	537.	733.435	{720.499, 746.39}	36.5801	0.135345	newly	173.	247.33	{228., 265.}	42.9653	0.154583	Reff	0.31905	0.266669	{0.263087, 0.269707}	16.4177	ar	8978.	9156.27	{9127., 9183.}	1.98563
47	R0	{2.88885, 16.8987, 0.1}	{2.96978, 18.8857, 0.}	{{2.96978, 18.8857, 0.}, {2.96978, 18.8857, 0.}}	{2.80151, 11.7585, 100.}	inf	414.	443.624	{435.406, 452.071}	7.15559	0.0299729	newly	129.	142.02	{127., 154.}	10.9767	0.0446128	Reff	0.22533	0.222845	{0.220248, 0.22525}	1.15971	ar	9337.	9365.3	{9348., 9381.}	0.305666
48	R0	{2.58709, 13.8786, 0.1}	{2.688, 14.8279, 2.}	{{2.688, 14.8279, 2.}, {2.688, 14.8279, 2.}}	{3.90048, 6.84033, 1900.}	inf	256.	294.996	{286.424, 302.691}	15.2328	0.0614561	newly	109.	116.27	{105., 129.}	8.65138	0.0355845	Reff	0.243704	0.242727	{0.240531, 0.245007}	0.667959	ar	9146.	9188.48	{9171., 9211.}	0.465121
49	R0	{2.42959, 10.7132, 0.1}	{2.61341, 12.5436, 2.}	{{2.61341, 12.5436, 2.}, {2.61341, 12.5436, 2.}}	{7.56586, 17.0858, 1900.}	inf	116.	207.433	{199.344, 216.743}	78.8215	0.252224	newly	60.	95.18	{85., 105.}	58.6333	0.199127	Reff	0.288149	0.286055	{0.283565, 0.287911}	0.771945	ar	8863.	8982.05	{8961., 9000.}	1.34322
50	R0	{2.60513, 13.2796, 0.1}	{2.98558, 18.1089, 1.}	{{2.98558, 18.1089, 1.}, {2.98558, 18.1089, 1.}}	{14.6039, 36.3667, 900.}	inf	243.	429.961	{419.979, 438.449}	76.9386	0.247768	newly	108.	144.53	{133., 157.}	33.8241	0.125607	Reff	0.256605	0.238261	{0.235694, 0.241137}	7.14882	ar	9095.	9323.71	{9300., 9347.}	2.51468
